# Research on the impact of ESG performance on carbon emissions from the perspective of green credit

**DOI:** 10.1038/s41598-024-61353-3

**Published:** 2024-05-07

**Authors:** Xiangrong Kong, Zhezhou Li, Xiao Lei

**Affiliations:** 1 School of Finance, Fuzhou University of International Studies and Trade, Fuzhou, 350202 China; 2 International Business School, Fuzhou University of International Studies and Trade, Fuzhou, 350202 China; 3 School of Information Management, Beijing Information Science and Technology University, Beijing, 100192 China

**Keywords:** ESG performance, Carbon emissions, Green credit, Threshold effect, Environmental impact, Sustainability

## Abstract

Utilizing panel data from 30 Chinese provinces, this research examines the non-linear relationship between regional environmental, social, and governance (ESG) performance and carbon emissions (CE) from the viewpoint of green credit. The study reveals a single threshold effect between ESG performance and CE, with green credit acting as the threshold variable. When the amount of green credit in a region exceeds the threshold, the growth rate of CE in that region begins to decline with higher ESG scores. Furthermore, green credit acts as a catalyst, playing a negative moderating role between ESG performance and CE, validated by both threshold regression and fixed effects models on panel data. Green credit indirectly influences carbon emissions by supporting green innovation, thus facilitating the transition to a greener economic development framework. Lastly, regional disparities are found in the moderating influence of green credit between ESG performance and CE. In regions with high ESG performance, the moderating impact of green credit is smaller, while in regions with low ESG performance, the effect is more significant. The research findings offer theoretical backing for policymakers regarding the efficacy of ESG in achieving carbon neutrality objectives, and offer valuable strategic recommendations for the diversified formulation of green credit strategies on both national and provincial scales. Regional heterogeneity test results provide valuable support for formulating policies that encourage green credit in provinces with low ESG performance.

## Introduction

According to the United Nations definition^1^, carbon neutrality refers to achieving a balance between carbon dioxide emissions and absorption from human activities on a global scale over a certain period of time. By the end of 2020, over 100 countries worldwide had set carbon neutrality goals. In September 2020, China made a commitment "Peak carbon dioxide emissions before 2030 and reach carbon neutrality before 2060". Given that China presently stands as the world's foremost carbon emitter, the capacity to significantly curtail carbon emissions (CE) stands as a paramount variable in realizing carbon neutrality objectives^2^. The notion of ESG was initially introduced in 2004 by organizations such as the UN Global Compact (UNGC). The ESG concept encourages investors to consider nonfinancial factors such as environmental management effectiveness and eco-friendly development strategies^3,4^. ESG is regarded as an important lever for implementing carbon neutrality goals^5^. ESG framework bring fresh prospects for promoting the sustainable growth of environmentally-friendly economy^6^.

The relationship between CE and ESG effectiveness has garnered widespread attention^7^. ESG performance is essential to drive CE reduction and achieve eco-friendly, low-emission, and sustainable growth^8,9^. Scholars have developed ESG evaluation metrics from various perspectives or by considering industry-specific characteristics^10,11^. Standardized ESG reports using the Global Reporting Initiative (GRI) standards have played a soft substitution role in advancing the vision of carbon neutrality, especially in institutional environments lacking a strong awareness of global warming^12,13^. Moreover, green credit plays a coordinating role between regional ESG performance and regional CE. Cong, et al.^14^ indicate that a 1% rise in ESG investment results in a 0.246% reduction in CO_2_ emissions and a 0.558% decrease in carbon emission intensity. While green credit is considered a means to provide funding for sustainable development, the current implementation of reforms requires further improvements to enhance its effectiveness^15^. In this regard, China has implemented a range of eco-friendly financial measures, such as environmental taxes, green insurance, and green credits^16^.

The beneficial effect of ESG performance on CE reduction has been widely recognized, but the specific nature of their relationship warrants further investigation. Yang et al.^17^ found a U-shaped connection between ESG performance and green innovation, and ESG performance determines whether government subsidies for green innovation exert a beneficial or detrimental influence. Due to the negative correlation between green innovation and CE^18^, there theoretically exists a non-linear relationship similar to a reverse U-shaped relationship between CE and ESG performance. The following are the marginal contributions of this article. First, we confirmed the non-linear relationship between ESG performance and CE using a threshold regression model, with green credit acting as the threshold variable. Second, green credit acts as a catalyst, exerting a negative moderating influence on the relationship between ESG performance and CE. Green credit indirectly influences carbon emissions by supporting green innovation. Lastly, we observed regional disparities in the moderating effect of green credit between ESG performance and CE, providing theoretical support for green credit policies.

The arrangement of this paper is outlined below: "Literature review" provides an overview of the literature, and "Mechanism analysis" suggests a theoretical hypothesis. "Study design" provides a description of the research design, including the model construction, choice of variables, data origins, and statistical summary. The empirical analysis is depicted in "Empirical analysis", and the results, restrictions, and directions for further study are outlined in "Conclusions".

## Literature review

Investigation into the connection between ESG performance and CE primarily includes the following aspects. First, empirical studies directly demonstrate the positive effect of ESG performance on CE reduction^19^. Second, analyzing contribution of ESG performance to stimulating green innovation, and the inhibitory influence of green innovation on CE, indirectly validates the promotion effect of ESG performance on CE reduction^17,18^. Third, from the perspectives of monetary regulation, green investment, green credit, bank stability, etc., analyzing the inhibitory influence of ESG on carbon emissions^14,20^.

### ESG’s impact on CE reduction

Research on the influence of ESG performance on CE reduction primarily focuses on the corporate level, with only a small number of studies at the provincial and regional levels. Cong, et al.^14^, based on empirical data from Chinese listed firms, found that the eastern region's environmental investments greatly increased carbon productivity, while the western and central areas saw large reductions in CE, albeit with a minor impact on carbon productivity. Guo et al.^21^ suggests that the impact of green credit on CE is more noticeable in Chinese coastal regions compared to inland areas, while green credit serves as a significant catalyst for regional ESG performance. ESG performance has a notable suppressive impact on CE, promoting CE reduction by easing the financial restrictions on corporations and solving agency issues^8^. In companies operating under strict environmental regulations, the effect of ESG performance on CE reduction is more significant^22^. Furthermore, a significant portion of ESG criteria is directly related to the environment and emissions, enabling to plan for carbon neutrality targets^23^.

### ESG’s promotion of green innovation

Scholars have pointed out that green innovation supports the "dual carbon" goals^24,25^. Differences in ESG ratings have a favorable influence on corporate eco-innovation, more pronounced in companies with higher independent director resources and greater media attention^26^. The enhancement of the country's ESG performance considerably stimulates green invention, especially in nations with less robust green innovation potential^27^. Third-party ESG assessments can effectively stimulate corporate green innovation, with corporations rated by ESG agencies showing a notable 3.9% surge in green invention output^28^. Some research has verified that green innovation suppresses carbon emission intensity, with more significant effects observed in regions with elevated CE intensity via "targeted accountability" and "reverse compulsory systems"^18^.

### The role of green credit

Currently, research on the role of green credit in relate with ESG mainly focuses on the effect of CE reduction. Wu, et al.^20^ indicate a nonlinear beneficial correlation between the level of money supply and CE. Green credit, as a significant component of money supply, should exert some form of nonlinear impact on carbon emissions. The government has implemented the Green Credit Guidelines to steer corporate behavior towards more environmentally friendly practices^29,30^. Several scholars have examined the influence of China's policies on CE by manually identifying regional green credit measures enacted by different municipalities^31,32^. Some scholars investigated the connection between ESG activities and banking value and found a non-linear relationship between them^33^. It becomes apparent that green credit policies play a crucial role in shaping corporate behavior towards more sustainable practices, ultimately adding to the broader goal of reducing CE and promoting environmental sustainability.

In general, there is a scarcity of studies on the nonlinear correlation between ESG performance and CE, with most current studies not specifying the specific form of interaction between the two. Existing research often focuses solely on the individual analysis of external factors such as green credit in promoting CE reduction, without considering green credit as a threshold variable to moderate the effects of ESG performance on CE. This paper contributes to existing studies by demonstrating the establishment of a non-linear association between ESG performance and CE. Green credit not only influences the growth rate of CE as a threshold variable but also acts as a detrimental moderator between ESG performance and CE, expanding the scope of prior research.

## Mechanism analysis

The Porter hypothesis suggests that achieving a win–win situation for both economic and ecological concerns is contingent upon a continual rise in Total Factor Productivity^34^. In a growing economy, fast-paced economic growth often comes to the detriment of the environment in the short term^35^. Whether it was the industrialization period in Europe and the United States over a century ago, or the past three decades in China, the rapid development of industrial economies has led to an increase in pollutant emissions and a significant rise in carbon emissions (CE) during specific periods. At the enterprise level, carbon emissions also increase rapidly as companies experience rapid growth^36^. When ESG evaluation criteria are introduced, companies strive to improve their ESG performance by reducing emissions of pollutants such as nitrogen oxides and greenhouse gases. In the long run, this action will lead to a decline in a company's own CE and subsequently impact the regional carbon emissions^37^. However, this process is not linear^38^. ESG performance consists of three dimensions: Environmental, Social, and Governance. Improvements in the Social and Governance dimensions can enhance the overall ESG score but may not immediately reduce a company's carbon emissions. As the Environmental dimension improves and the overall emission reduction effect of ESG increases, a company's CE will experience a decrease in growth rate. The total CE will initially increase and then decrease until reaching carbon neutrality. Similarly, as more representative companies in a region implement ESG standards, the total regional carbon emissions will also undergo a non-linear decline.

**Figure 1:**
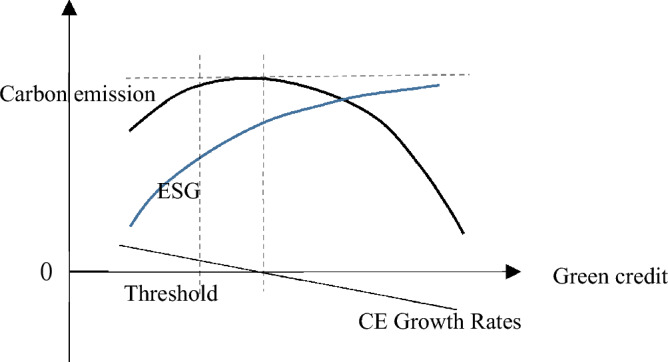
The non-linear correlation between Green credit, ESG and CE.

Green credit is a key initiative to enable the conversion and development of finance resources towards the green industry, aiming to incentivize green innovation and stimulate the economy's transition to low-carbon development^39^. The advancement of green finance synergizes with the improvement of ESG performance^40^. Green credit measures optimizes financial resource distribution, encourages green innovation activities by enterprises, enhances their ESG performance, and simultaneously influences the regional carbon emission level. As the level of green credit increases, ESG performance exhibits a similar trend, and the total carbon emissions also show an initial increase followed by a decrease, eventually leading to a trend towards carbon neutrality. Due to the nonlinear characteristic of the decline in carbon emissions, there may exist a threshold for green credit level, which triggers a transformative change in the influence of ESG performance on CE, Fig. 1 illustrates this trend.

Meanwhile, the three dimensions of ESG performance have different impacts on CE. The Environmental dimension (E dimension) requires companies to improve energy efficiency, reduce energy consumption, develop clean energy sources, and implement proper waste disposal and recycling. The influence of the E dimension on CE is direct and significant. The Social dimension (S dimension) emphasizes the company's social responsibility towards employees, who are important stakeholders. Fulfilling social responsibilities towards employees, such as ensuring their health and safety, can contribute to better social performance for the company. The Governance dimension (G dimension) focuses on optimizing internal governance structures and establishing a "green culture" that integrates energy-saving and emission reduction practices into various departments and project management, thus affecting CE^41^. The impact of the S and G dimensions on carbon emissions is indirect and lagging.

Hypothesis 1: There exists a non-linear correlation between overall ESG performance and CE. When the green credit level in a region exceeds the threshold, a rise in ESG scores result in a decline in the growth rate of CE within the region. The E, S, and G sub-indicators have different effects on CE.

ESG performance driven by green innovation has stronger environmental externalities and requires higher investment^42^. It is necessary for green companies to acquire stable and long-term capital. The core of green credit is to allocate credit resources based on the assessment of a company's ESG performance, aiming to channel more funds into green industries and non-polluting companies to promote their green innovation^43^.On one hand, regions with higher levels of green credit and better development of green finance impose more environmental and social performance constraints on companies when applying for loans. Therefore, companies with good ESG performance receive benefits and obtain more loan support, allowing them to allocate more funds to green innovation activities, further enhancing their ESG performance, establishing their technological competitive advantage^44^. On the other hand, companies with poor ESG performance face difficult situations and realize that they need to improve their ESG performance in order to alleviate loan constraints and obtain more loan support, which supports their long-term development and independent innovation. In both cases, green credit acts as a catalyst by providing more loan support to companies with outstanding ESG performance, thereby enhancing regional green innovation level. In other words, green credit strengthens the influence of ESG performance on green innovation levels, leading to a reduction in regional CE and playing a moderating role between regional ESG performance and CE in a negative direction.

Additionally, there might be a positive moderating influence of green credit between ESG performance and CE. The phenomenon of "green washing" refers to companies exaggerating their ESG levels through various means to mislead investors and gain more profits^45^. This phenomenon is becoming increasingly prevalent in ESG reports and is a significant issue that cannot be ignored. Companies rely on inflated ESG performance to obtain loan support from banks. However, in pursuit of profits, these companies may not invest significant costs in green innovation research for long-term development. Instead, they use the loans to expand their industrial scale or develop new products that yield quick returns^46^. In such cases, green credit fails to allocate credit resources effectively, and it weakens the influence of regional ESG performance on reducing CE. The moderating effect of green credit between regional ESG performance and CE depends on the reliability of ESG performance. It relies on whether the ESG evaluation criteria are appropriate. The accuracy and relevance of data, valuation models, and methods will affect the ability to identify and avoid "green washing" risks, and rationally allocate credit resources.

Hypothesis 2: The association between ESG performance and CE is moderated by green credit.

Hypothesis 2a: Green credit has a positive moderating effect between ESG performance and CE.

Hypothesis 2b: Green credit has a negative moderating effect between ESG performance and CE.

Green credit plays a moderating role between ESG performance and CE, but its effectiveness vary due to regional development disparities. Regions with better ESG performance are typically more economically developed, with higher profitability, making it easier to access abundant green finance resources^47^. However, due to the non-linear relationship between ESG performance and carbon emissions and the negative moderating effect of green credit, as regions with high ESG performance start to experience a decline in CE, a significant influx of green credit may not proportionally reduce regional carbon emissions. Conversely, the utility of green credit resources for CE mainly comes from the E dimension. In economically developed regions, there is already a strong capacity for green technological innovation^48^, green credit support cannot have a greater impact because the high performance in the S and G dimensions contributes to the overall ESG performance. In contrast, in regions with low ESG performance, which are often characterized by lower levels of economic development and greater environmental damage during the economic development, green credit resources can have a greater moderating effect. In these regions, the E dimension has a stronger impact on CE, and the availability of green credit resources can directly drive improvements in E dimension.

Hypothesis 3: Regional disparities exist in the moderating influence of green credit between corporate ESG performance and CE. In regions exhibiting high ESG performance, the moderating influence of green credit is small. In regions exhibiting low ESG performance, the moderating influence of green credit is more pronounced.

## Study design

The non-linear relationship between variables is better captured by the threshold regression model. The relationship between ESG performance and CE may go through several stages or have significant influencing factors that a linear model is unable to adequately capture. To more precisely identify this non-linear link, a threshold regression model is constructed following.

### Selection of variables

#### Explained variables

This article chooses the data source of CE from 30 Chinese provinces during the time frame of 2011–2020 as explained variables. The CE coefficients issued by the Intergovernmental Panel on Climate Change (IPCC) in 2006 are used in conjunction with the consumption of the top ten fossil fuels, comprising natural gas, liquefied petroleum gas, coal, coke, and more, to estimate CE^49^. The calculation formula is as follows:

1 $$ CE = \sum {_{i = 1}^{8} } E_{i} \times LH_{i} \times CH_{i} \times COR_{i} $$

where ${\text{CE}}$ represents the total carbon emissions of each province, ${\text{E}}_{i}$ represents the total consumption of fossil fuels *i*, $LH_{i}$ denotes the average low heating value of fuel *i*, $CH_{i}$ stands for the carbon content per unit of heat of fuel *i*, and $COR_{i}$ represents the carbon oxidization rate of fuel *i*.

#### Explanatory variables

The corporate ESG performance. The ESG scores of A-share listed companies from 2011 to 2020 were obtained from the Bloomberg ESG database^50^, including overall score, E-score, S-score, and G-score. Data were collected for companies with valid ESG scores (excluding records with missing scores). In 2011, there were over 800 included companies, while the number increased to over 1300 from 2020 onwards. Considering the availability of actual ESG scores, the ESG performance of listed companies was aggregated at the provincial level to investigate its impact on provincial CE intensity. The computation formula is given below:

2 $$ ESG_{total} = \sum {_{i = 1}^{n} } ESG_{i} \times \delta _{i} $$

where $\delta _{i}$ symbolizes the ratio of the annual output of company *i* to the total annual output of all listed companies in the province in that year, $ESG_{i}$ represents the ESG score of company *i*, *n* stands for the quantity of all listed companies in the province in that year, and $ESG_{total}$ represents the ESG performance of the province.

#### Threshold variable

Green credit (Gcredit) is the threshold variable. In this study, a panel data threshold effect framework is utilized to examine the nonlinear influence of ESG on CE under different levels of green credit. Currently, research on ESG and green credit is mainly focused on the banking industry, so we use the ratio of eco-friendly loans provided by banks to represent the level of green credit. The ratio of interest costs of the six largest, most energy-intensive industries to the total industrial interest costs is calculated^51^. The six industries include chemicals, petroleum, electricity and heat power, non-metallic minerals, ferrous metals, and non-ferrous metals, and the data comes from the "China Industrial Statistics Yearbook." At the same time, green credit serves as the moderating variable between ESG performance and CE.

#### Control variables

Choosing appropriate control variables can enhance the accuracy and reliability of research findings. Referring to Zhang, et al.^52^and Liu, et al.^53^ studies, this article chooses the proportion of GDP spending on pollution prevention to indicate environmental regulation (Regu). Government financial strength (Gov)^54^is a crucial metric to show the degree of economic development in a province, expressed as the ratio of total state budget expenditure to GDP. Per capita GDP (Pgdp)^55^ is also a significant variable affecting the level of regional economic development. In areas with high per capita GDP, the per capita consumption of resources is higher, which can easily result in CE increase. Population density (Popu)^56^ is also a dimension affecting carbon emissions. In areas with high population density, the consumption of environment and resources is greater. The overall electricity usage (Ele)^57^of the entire community, representing the total amount of electricity consumed by a province in a year, is an important indicator of industrial economic development and has an indirect influence on CE.

### Model construction

This paper primarily examines the non-linear relationship between ESG performance and CE, and investigates the varied influence of the three sub-dimensions of ESG on CE. Moreover, the moderating influence of green credit is analyzed using a threshold effect model and a fixed effects model, considering regional variations within the moderating impact. Following the threshold effect model proposed by Hansen^58^, we construct a single threshold effect model for corporate ESG and carbon emissions as follows:

3 $$ \begin{gathered} {\text{lnCE}}_{it} = \ln {\text{A}}_{i0} + a_{i1} \ln {\text{ESG}}_{it}^{} (\ln Gcredit_{it}^{} \le \theta_{1} ) + a_{i2} \ln {\text{ESG}}_{it}^{} (\theta_{1} \le Gcredit_{it}^{} \le \theta_{2} ) + \cdots \hfill \\ + a_{i3} \ln {\text{ESG}}_{it}^{} (\ln Gcredit_{it}^{} \ge \theta_{q} ) + \beta_{i} \ln Gov_{it}^{} + \gamma_{i} \ln {\text{Regu}}_{it}^{} + \delta_{i} \ln Pgdp_{it}^{} + \phi_{i} \ln {\text{Popu}}_{it}^{} + \lambda_{i} \ln {\text{Ele}}_{it}^{} + \varepsilon_{it} {, } \hfill \\ \end{gathered} $$

where ${\text{CE}}_{it}$ represents the explained variable of carbon emissions, *i* stands for province, t stands for year. ${\text{ESG}}_{it}^{}$ stands for the explanatory variable, the overall ESG performance of province *i* in year t, which can be further decomposed into three dimensions: E, S, and G. $Gcredit_{it}^{}$ is the threshold variable, reflecting the non-linear correlation, also serves as the moderating variable, $\theta_{1} , \cdots \theta_{q}$ is the threshold value of the model. $Gov$, ${\text{Regu}}$, $Pgdp$, ${\text{Popu}}$, ${\text{Ele}}$ are control variables. ${\text{lnA}}_{i0}$ denotes the model intercept, $a_{i}$,$\beta_{i}$,$\gamma_{i}$,$\delta_{i}$,$\phi_{i}$ and $\lambda_{i}$ are the coefficients, and $\varepsilon_{it}$ denotes the error term.

To examine the moderating effect of green credit, we formulated the following fixed effects model:

4 $$ {\text{lnCE}}_{it} = \alpha_{i} + \lambda_{i} \ln {\text{ESG}}_{it}^{} + \sum {control_{it} + \mu_{it} } $$

5 $$ {\text{lnCE}}_{it} = \alpha_{i} + \rho_{i} \ln {\text{Gcredit}}_{it}^{} + \sum {control_{it} + \mu_{it} } $$

6 $$ {\text{lnCE}}_{it} = \alpha_{i} + \lambda_{i} \ln {\text{ESG}}_{it}^{} + \rho_{i} \ln {\text{Gcredit}}_{it}^{} + \sum {control_{it} + \mu_{it} } $$

7 $$ {\text{lnCE}}_{it} = \alpha_{i} + \lambda_{i} \ln {\text{ESG}}_{it}^{} + \rho_{i} \ln {\text{Gcredit}}_{it}^{} + \omega_{i} \ln {\text{ESG}}_{it}^{} *\ln {\text{Gcredit}}_{it}^{} + \sum {control_{it} + \mu_{it} } $$

Among them, ${\text{lnESG}}_{it}^{} *\ln {\text{Gcredit}}_{it}^{}$ represents the interaction term. Equation (4) analyzes the impact of ESG performance on CE. Equation (5) examines the effect of green credit on CE. Equation (6) analyzes the impact of ESG performance and green credit on CE simultaneously. Equation (7) adds the interaction term between ESG performance and green credit, which is the pivotal aspect of the test. If the interaction term and the main effect of ESG performance on CE are both significant, it indicates that green credit plays a moderating role. If the signs of both variables are the same, the moderating effect is positive; otherwise, it is negative.

Similarly, we can verify the impact of the interaction term using the threshold effect model, as shown in the following equation:

8 $$ \begin{gathered} {\text{lnCE}}_{it} = \ln {\text{A}}_{i0} + a_{i1} \ln {\text{ESG}}_{it}^{} (\ln {\text{ESG}}_{it}^{} *\ln {\text{Gcredit}}_{it}^{} \le \theta_{1} ) + a_{i2} \ln {\text{ESG}}_{it}^{} (\theta_{1} \le \ln {\text{ESG}}_{it}^{} *\ln {\text{Gcredit}}_{it}^{} \le \theta_{2} ) + \cdots \hfill \\ + a_{i3} \ln {\text{ESG}}_{it}^{} (\ln {\text{ESG}}_{it}^{} *\ln {\text{Gcredit}}_{it}^{} \ge \theta_{q} ) + \beta_{i} \ln {\text{Gcredit}}_{it}^{} + \sum {control_{it} } + \varepsilon_{it} { ,} \hfill \\ \end{gathered} $$

We also consider the effects of interaction terms for the social, environmental, and governance dimensions separately by setting the threshold variables as $\ln {\text{E}}_{it}^{} *\ln {\text{Gcredit}}_{it}^{}$, $\ln {\text{S}}_{it}^{} *\ln {\text{Gcredit}}_{it}^{}$ and $\ln {\text{G}}_{it}^{} *\ln {\text{Gcredit}}_{it}^{}$, for comprehensive comparative analysis.

### Sources of data and descriptive statistics

Using samples from 30 Chinese provinces or regions (excluding Tibet, Hong Kong, Macao, and Taiwan) spanning from 2011 to 2020, we analyze the impact of ESG performance on CE from the perspective of green credit. The CE data originate from the "China Energy Statistical Yearbook", including the consumption of nine industries: coal, coke, crude oil, gasoline, kerosene, diesel, fuel, liquefied petroleum gas, and natural gas, converted into total carbon dioxide emissions. The ESG performance data come from Bloomberg's ESG database of listed enterprises on the A-share market of China, the environmental regulation data originate from the "China Environmental Statistical Yearbook", and the rest of the data come from the China Statistical Yearbook. The main characteristics of descriptive statistics are shown in Table 1.

**Table 1:** Variables' descriptive statistics.

Variable	N	Mean	SD	Min	Max
CE	300	43,202	30,599	4880	151,500
ESG	300	29.18	7.256	15.76	48.28
Gcredit	300	0.480	0.153	0.094	0.808
Gov	300	0.264	0.115	0.120	0.758
Regu	300	0.0152	0.0105	0.0001	0.0936
Pgdp	300	53,837	27,036	16,024	164,200
Popu	300	472.8	704.3	8	3925
Ele	300	2008	1438	185.3	6940
ESG	300	29.18	7.256	15.76	48.28

N represents the sample size, collected from 30 provinces over a continuous period of 10 years.

## Empirical analysis

### Regression results of ESG performance and carbon emissions

The outcomes of the threshold regression test indicate a single threshold effect between corporate ESG performance and CE, with a significance level of 1.43%. However, the double and triple threshold regressions are not statistically significant. This suggests that the critical change between corporate ESG performance and regional carbon emissions is stable and does not exhibit multiple reversals. The threshold variable, green credit (lnGcredit), has a threshold value of -1.2324, calculated value of Gcredit is 0.2923, indicating that when the fraction of green credit exceeds 0.2923 in a province, the correlation between corporate ESG performance and CE in the region undergoes a significant change. Table 2 displays the test results.

**Table 2:** Threshold effect test and threshold value of ESG and CE.

Threshold quantity	F value	P value	Threshold value
Single	27.59	0.0143	-1.2324
Double	3.78	0.8571	-0.8358
Triple	7.18	0.5514	-0.7667

Based on the baseline regression model's findings, as displayed in Table 3, the regression coefficient between ESG performance and CE is generally positive, showing that the advancement in corporate ESG does not immediately play a crucial role in CE reduction. Despite the improvement in ESG performance, the total CE in the region continue to rise. This result is not contradictory to the conclusion of Li and Xu^8^, which suggests that ESG ratings exert a pronounced inhibiting effect on CE. This paper extends the analysis of ESG to the regional level and also takes into account the lagged effect of ESG improvement on most high-emission companies. At the regional level, the improvement in ESG performance may coexist with an increase in CE in the short term, followed by an accelerated decline process, resembling an inverted U-shaped pattern. This corresponds with the conclusions of Yang, et al.^17^ regarding the U-shaped correlation between ESG performance and green innovation, as green innovation has a negative relationship with CE^18^, reversing the U-shaped curve.

**Table 3:** The threshold regression results of ESG and CE.

Variable	Regression coefficient	Standard error
lnESG (lnGcredit $\le \theta_{1}$)	0.0857**	0.0397
lnESG (lnGcredit $> \theta_{1}$)	0.0439	0.0408
lnRegu	0.0158*	0.0085
lnGov	−0.1144**	0.0545
lnPgdp	−0.3364***	0.0489
lnPopu	0.2319	0.1627
lnEle	0.6657***	0.0524
_cons	7.6188***	0.8156
R^2^	0.537	
F	43.5166	

Asterisk notation denotes P < 0.1 for *, 0.05 for **, and 0.01 for ***.

Within the selected sample period from 2011 to 2020, China's economic evolution pattern still relies on the consumption of resources and environmental damage. The rapid growth of the industrial has led to a rise in pollutant emissions, and CE have continued to rise during specific periods. It is encouraging to find that there is a non-linear relationship between corporate ESG performance and CE. The regression results indicate that when the level of regional green credit is high and exceeds the threshold value, the impact of provincial ESG performance on CE is significantly enhanced, resulting in a significant decrease in the growth rate of CE in the region. This finding confirms Hypothesis 1.

The coefficient between government environmental Regu and CE is positive, indicating that in regions with higher carbon emissions, the government is more proactive in implementing environmental regulations and allocating resources to environmental control. The coefficients of Gov and Pgdp are negative, suggesting that provinces with higher government fiscal income and stronger per capita economic strength have lower levels of CE. It can be inferred that regions with strong per capita economic capacity, such as Beijing and Shanghai, have achieved economic development without relying on environmental degradation to some extent. The coefficients of Popu and Ele are positive, suggesting that regions with higher population density and higher electricity consumption levels have higher carbon emissions.

Furthermore, this study conducted threshold regression analyses between the three sub-dimensions of ESG and CE. The regression outcomes, as depicted in Table 4, indicate that all three dimensions exhibit significant first-order threshold effects on CE, but their coefficients differ. Regarding the Environmental dimension, when the level of green credit represented by Gcredit is below the threshold value, the E dimension is positively related to CE with a coefficient of 0.0295, at the 1.25% significance. However, when Gcredit surpasses the threshold value, the relationship between the E dimension and CE reverses and becomes negative, with a coefficient of -0.0256, at the 1.19% significance. This indicates that in regions where the green credit level exceeds the threshold, a rise in the E dimension score leads to a decline in CE, providing strong theoretical support for local governments to develop green credit and promote the attainment of carbon neutrality goals.

**Table 4:** The threshold regression results of environmental, social, governance and CE.

Variable	lnCE	lnCE	lnCE
lnE (lnGcredit $\le \theta_{1}$)	0.0295** (0.0125)		
lnE (lnGcredit $> \theta_{1}$)	−0.0256** (0.0119)		
lnS (lnGcredit $\le \theta_{1}$)		0.0649** (0.0286)	
lnS (lnGcredit $> \theta_{1}$)		0.0053 (0.0292)	
lnG (lnGcredit $\le \theta_{1}$)			0.0881** (0.0353)
lnG (lnGcredit $> \theta_{1}$)			0.0559 (0.0360)
lnRegu	0.0070 (0.0082)	0.0128 (0.0081)	0.0157* (0.0083)
lnGov	−0.0906* (0.0529)	−0.0996* (0.0525)	−0.1232** (0.0543)
lnPgdp	−0.2982*** (0.0430)	−0.3055*** (0.0413)	−0.3356*** (0.0448)
lnPopu	0.2361 (0.1632)	0.2125 (0.1625)	0.2421 (0.1628)
lnEle	0.7119*** (0.0534)	0.6677*** (0.0524)	0.6609*** (0.0525)
_cons	−2.1641*** (0.6818)	−0.5639 (0.4829)	−1.5939*** (0.5978)
N	300	300	300
R^2^	0.529	0.536	0.536
F	42.2091	43.4599	43.4424

Asterisk notation denotes P < 0.1 for *, 0.05 for **, and 0.01 for ***

Conversely, the Social and Governance dimensions do not exhibit such pronounced reversal effects. The regression coefficients remain positive, but when the S and G dimension scores surpass the threshold value, their coefficients decrease in absolute value, consistent with Hypothesis 1. This suggests that when the level of green credit in an area is higher and the proportion of green credit exceeds the threshold value, higher S and G dimension scores correspond to a decrease in the growth rate of CE. Therefore, it can be observed that the influence of the E dimension on CE is direct and significant. This is because an increase in the E dimension score requires companies to directly improve energy efficiency and achieve a direct reduction in CE. However, the effects of the S and G dimensions on CE are indirect and lagged. This finding is consistent with our analysis and inference in Hypothesis 1.

In terms of control variables, the coefficients of Regu, Popu, and Ele are positive, while the regression coefficients of Gov and Pgdp are negative. These outcomes align with the coefficients noted in the baseline regression, thus validating the stability of the model.

### The moderating effect of green credit

This study employed both fixed effects models and threshold effects models to verify the moderating influence of green credit. The test outcomes of the fixed effects regression are presented in Table 5. Column (1) examines the correlation between ESG performance and CE, revealing a positive relationship, a coefficient of 0.0895, and significant level below 5%. This suggests that the advancement in ESG performance does not immediately impact CE, and short-term economic development still involves sacrificing a portion of the environment and resources. Column (2) examines the relationship between Gcredit and CE, demonstrating a negative relationship with a coefficient of -0.0275. This implies that the growth of green credit contributes to the reduction of CE, in line with the results of Jiang, et al.^32^ and Hu, et al.^31^. However, this paper goes further to discuss the function of green credit by extensively examining the interaction between green credit and ESG.

**Table 5:** The fixed effects regression results of the moderating effect.

Variable	(1)	(2)	(3)	(4)
	lnCE	lnCE	lnCE	lnCE
lnESG	0.0895** (0.0415)		0.0868** (0.0424)	−0.0870 (0.0618)
lnGcredit		−0.0275 (0.0376)	−0.0126 (0.0381)	0.5832*** (0.1620)
lnESG_Gcredit				−0.1742*** (0.0461)
lnRegu	0.0156* (0.0089)	0.0092 (0.0085)	0.0152* (0.0090)	0.0115 (0.0088)
lnGov	−0.1365** (0.0567)	−0.0975* (0.0561)	−0.1323** (0.0582)	−0.0649 (0.0596)
lnPgdp	−0.3705*** (0.0506)	−0.2956*** (0.0419)	−0.3647*** (0.0536)	−0.3739*** (0.0524)
lnPopu	0.2937* (0.1694)	0.2731 (0.1705)	0.2958* (0.1698)	0.3407** (0.1661)
lnEle	0.6713*** (0.0547)	0.6727*** (0.0564)	0.6673*** (0.0562)	0.7029*** (0.0556)
_cons	7.4418*** (0.8511)	7.0405*** (0.8486)	7.4006*** (0.8616)	7.6667*** (0.8436)
N	300	300	300	300
R^2^	0.493	0.485	0.493	0.519
F	42.7122	41.3852	36.5025	35.3393

Asterisk notation denotes P < 0.1 for *, 0.05 for **, and 0.01 for ***.

Column (3) includes both ESG performance and Gcredit in the model, and the results align with columns (1) and (2), suggesting the stability of the previous outcomes. In column (4), the interaction term is introduced, aiming to examine whether green credit considerably influences the connection between ESG performance and CE. The outcomes reveal that the coefficient of the interaction term is negative, contrary to ESG performance in column (1). This indicates that green credit exerts a negative moderating role in the correlation between ESG performance and CE, suggesting that it facilitates the reduction of CE based on ESG performance. Hypothesis 2b is confirmed.

The findings of the threshold effects model are presented in Table 6. Column (1) examines the relationship between the interaction term and CE, revealing a negative relationship, at a significant level below 1%. This aligns with the results of column (4) in the fixed effects model in Table 4. Columns (2), (3), and (4) represent the relationships between the interaction terms and CE under the E, S, and G dimensions, respectively, all demonstrating significant negative relationships. This also confirms Hypothesis 2b. Notably, column (2) exhibits a noticeable "U-shaped" turning point in the relationship between the E dimension's interaction term and CE, similar to the relationship between ESG and CE discussed earlier. When green credit exceeds a certain threshold, an increase in the E dimension score may lead to an increase in CE, thereby showing the "green washing" effect. The precision and efficiency of the data will influence the bank's identification and mitigation of "green washing" risks, thereby affecting the rational allocation of credit resources and having adverse effects on carbon CE.

**Table 6:** The findings of the moderating effect.

Variable	(1)	(2)	(3)	(4)
	lnCE	lnCE	lnCE	lnCE
lnESG_Gcredit (lnGcredit $\le \theta_{1}$)	−0.1175*** (0.0300)			
lnESG _Gcredit (lnGcredit $> \theta_{1}$)	−0.0891*** (0.0311)			
lnE_Gcredit (lnGcredit $\le \theta_{1}$)		−0.0163** (0.0076)		
lnE _Gcredit (lnGcredit $> \theta_{1}$)		0.0296** (0.0114)		
lnS_Gcredit (lnGcredit $\le \theta_{1}$)			−0.1049*** (0.0252)	
lnS _Gcredit (lnGcredit $> \theta_{1}$)			−0.0654** (0.0266)	
lnG_Gcredit (lnGcredit $\le \theta_{1}$)				−0.1061*** (0.0292)
lnG _Gcredit (lnGcredit $> \theta_{1}$)				−0.0837*** (0.0300)
lnGcredit	0.3479*** (0.1146)	−0.0626 (0.0388)	0.1885** (0.0739)	0.4051*** (0.1372)
lnRegu	0.0169** (0.0082)	0.0076 (0.0083)	0.0129 (0.0081)	0.0159* (0.0082)
lnGov	−0.1057** (0.0528)	−0.0976* (0.0544)	−0.1004* (0.0529)	−0.1137** (0.0531)
lnPgdp	−0.3880*** (0.0470)	−0.3004*** (0.0422)	−0.3457*** (0.0419)	−0.3727*** (0.0456)
lnPopu	0.2759* (0.1619)	0.2103 (0.1661)	0.2292 (0.1611)	0.2714* (0.1625)
lnEle	0.6952*** (0.0533)	0.7096*** (0.0555)	0.6763*** (0.0532)	0.6913*** (0.0536)
_cons	7.9223*** (0.8176)	7.1528*** (0.8251)	7.8293*** (0.8167)	7.7961*** (0.8165)
N	300	300	300	300
R^2^	0.547	0.523	0.545	0.543
F	39.5003	35.9079	39.2752	38.8366

Asterisk notation denotes P < 0.1 for *, 0.05 for **, and 0.01 for ***.

In terms of control variables, the coefficients of the fixed effects model in Table 5 and the threshold effects model in Table 6 exhibit similar characteristics to the baseline regression coefficients. The variables of environmental Regu, Popu, and Ele have positive coefficients, while government Gov and Pgdp have negative coefficients in relation to CE. This further confirms the stability of the model.

### Endogeneity and robustness

Panel data regressions commonly suffer from endogeneity issues, primarily stemming from three sources: measurement errors, omitted explanatory variables, and reverse causality. The empirical model in this paper faces these challenges as well. For instance, regional CE levels may, in turn, affect the implementation of green credit policies. Consequently, the estimation results of the baseline model may be biased. There are several methods to address endogeneity problems. Fixed effects models for panel data can partially address estimation errors caused by individual effects. Additionally, the most effective method is to use the Heckman two-stage estimation method with instrumental variables. Drawing on the research of Zhao, et al.^59^ , Zhang and Kong^60^, this paper constructs instrumental variables using the ratio of provincial GDP to financial institution deposits.

Table 7 reports the results of instrumental variable regression. The weak identification test results show that the mean of the Cragg-Donald Wald F statistic exceeds the 10% critical value of the Stock-Yogo test, passing the weak instrumental variable test. The LM statistic of the underidentification test rejects the null hypothesis at the 1% level, satisfying the instrument's identifiability. After considering endogeneity issues, the coefficients of the key explanatory variables, threshold variables, and moderating terms are consistent with the baseline regression coefficients, indicating a certain degree of stability in the estimation results of the baseline model.

**Table 7:** The instrumental variable and Heckman two-stage estimation results.

Variable	IV (2SLS)	FE
	lnCE	lnCE
lnESG	−0.3746* (0.2086)	−0.0870 (0.0618)
lnGcredit	1.5387** (0.6311)	0.5832*** (0.1620)
lnESG_Gcredit	−0.4398** (0.1737)	−0.1742*** (0.0461)
Cragg–Donald Wald F statistic	19.344 (16.380)	
Anderson canon. corr. LM statistic	19.038 (0.000)	
R^2^	0.467	0.519
N	300	300

Asterisk notation denotes P < 0.1 for *, 0.05 for **, and 0.01 for ***.

### Regional heterogeneity

To validate the regional heterogeneity of the moderating effect of green credit, this study employed two grouping methods: categorized by ESG level and by green credit level. Before conducting grouped regressions, we utilized two heterogeneity testing methods: Seemingly Unrelated Estimation (Suest) and Fisher's Combination Test. The test results yielded similar conclusions, indicating that the regional disparities induced by ESG level were more pronounced. The test results are presented in Table 8.

**Table 8:** Heterogeneity test results.

Test	Variable	Group by ESG	Group by Gcredit
		P value	P value
Suest	lnGcredit	0.048	0.197
	lnESG_Gcredit	0.026	0.079
Fisher's Combination	lnGcredit	0.090	0.440
	lnESG_Gcredit	0.050	0.460

The regression results for the full sample, High ESG group, and Low ESG group are presented in Table 9 for comparison. The findings indicate that in regions with higher ESG performance, as in column (2), the coefficient cannot be tested for significance at the chosen level. In contrast, in regions with lower ESG performance, presented in column (3), the coefficient of the interaction term is −0.3596, with its absolute value even larger than the full sample regression coefficient −0.1742. These results indicate significant differences between the two groups, supporting Hypothesis 3.

**Table 9:** The moderating effect in provinces with different ESG levels.

Variable	(1)All	(2)High ESG	(3)Low ESG
	lnCE	lnCE	lnCE
lnESG	−0.0870 (0.0618)	−0.1092 (0.0923)	−0.2589 (0.0920)
lnRegu	0.0115 (0.0088)	0.0124 (0.0102)	0.0014 (0.0148)
lnGov	−0.0649 (0.0596)	0.0311 (0.0866)	0.0023 (0.0844)
lnPgdp	−0.3739*** (0.0524)	−0.1713*** (0.0711)	−0.4884*** (0.0832)
lnPopu	0.3407** (0.1661)	−0.4310 (0.3074)	0.4810*** (0.2064)
lnEle	0.7029*** (0.0556)	0.7062*** (0.0596)	0.8078*** (0.1162)
lnGcredit	0.5832*** (0.1620)	0.0919 (0.2799)	1.0537*** (0.2162)
lnESG_Gcredit	−0.1742*** (0.0461)	−0.0173 (0.773)	−0.3596*** (0.0638)
_cons	7.6667*** (0.8436)	10.1686*** (1.7046)	8.0284*** (0.9897)
N	300	150	150
R^2^	0.5190	0.6841	0.4652
F	35.34	34.37	13.81

Asterisk notation denotes P < 0.1 for *, 0.05 for **, and 0.01 for ***

Guo, et al.^21^ suggests that the influence of green credit on CE in Chinese coastal areas is more significant than that in inland areas, thus indicating spatial variations in the influence of green credit on CE. The conclusion is not contradictory to the findings of this study. Coastal areas do not necessarily represent high ESG performance. In China, coastal provinces such as Hebei, Liaoning and Hainan have ESG performance lower than most inland provinces. This difference arises from that in regions with higher ESG performance, the overall economic development level is higher and there is no shortage of credit funds. Therefore, the promoting effect of green credit may not effectively impact CE reduction and may even lead to negative effects due to the potential "green washing" phenomenon. On the other hand, in regions with lower ESG performance, the overall economic development level is lower and there is a scarcity of credit funds. Thus, the support from green credit can drive companies to improve green technologies, enhance resource recycling, and promote carbon reduction activities more effectively.

Furthermore, we divided the sample data into High Gcredit group and Low Gcredit group, as illustrated in Table 10, but the outcomes did not exhibit significant differences. It is evident that the level of credit allocation in the regions does not affect the conclusions significantly, indicating that Gcredit is suitable as a threshold analysis variable. As for the control variables, the regression coefficients for the High ESG group and Low ESG group remained consistent with the baseline regression, further confirming the stability of the model.

**Table 10:** The moderating effect in provinces with different green credit levels.

Variable	(1)All	(2)High Gcredit	(3)Low Gcredit
	lnCE	lnCE	lnCE
lnESG	−0.0870 (0.0618)	−0.1189 (0.1376)	−0.0711 (0.0920)
lnRegu	0.0115 (0.0088)	0.0173 (0.0109)	−0.0044 (0.0163)
lnGov	−0.0649 (0.0596)	0.0399 (0.0768)	−0.1792* (0.0960)
lnPgdp	−0.3739*** (0.0524)	−0.3905*** (0.0897)	−0.3943*** (0.0739)
lnPopu	0.3407** (0.1661)	0.1476 (0.2095)	0.5806* (0.2955)
lnEle	0.7029*** (0.0556)	0.8868*** (0.1209)	0.6759*** (0.0703)
lnGcredit	0.5832*** (0.1620)	0.2296 (0.7081)	0.5599** (0.2384)
lnESG_Gcredit	−0.1742*** (0.0461)	−0.0646 (0.2024)	−0.1737** (0.0691)
_cons	7.6667*** (0.8436)	7.8101*** (1.1474)	6.8313*** (1.3065)
N	300	150	150
R^2^	0.519	0.386	0.615
F	35.3393	9.9818	25.3486

Asterisk notation denotes P < 0.1 for *, 0.05 for **, and 0.01 for ***.

## Conclusions

From the perspective of green credit, this research looks into the connection between company ESG performance and CE. Using sample data from 30 Chinese provinces, spanning from 2011 to 2020, a threshold model is constructed to verify the non-linear correlation between corporate ESG performance and CE. A fixed-effects model and threshold regression model are employed to compare the moderating effect of green credit on the correlation between ESG and CE, further discussing the regional heterogeneity of the moderating effect.

The study's findings lead to the following conclusions: Firstly, there is a non-linear correlation between ESG performance and CE. When the ESG performance of companies in a region exceeds a certain threshold, an increase in ESG scores leads to a downturn in the growth rate of regional CE. While all three dimensions—Environmental, Social, and Governance—exhibit single-threshold effects on CE, the impact of the E dimension is direct and significant, while the impacts of the S and G dimensions are indirect and lagging. Secondly, green credit exerts a negative moderating role between corporate ESG performance and carbon emissions. As a catalyst, green credit provides more loan support to companies with outstanding ESG performance, enabling them to invest significant funds in green innovation research for long-term development. This, in turn, enhances regional green innovation and contributes to a decline in CE. Lastly, the moderating effect of green credit between ESG performance and CE varies across regions. In regions with low ESG performance, green credit has a greater moderating effect.

### Theoretical implication

This paper has several theoretical implications. Firstly, it contributes to existing studies by demonstrating the establishment of a non-linear correlation between ESG performance and CE. Yang, et al.^17^ found a U-shaped correlation between ESG performance and green innovation, although some studies have suggested a negative correlation between green innovation and CE, there has not been an in-depth analysis of the specific functional form of ESG performance on CE. This paper empirically confirms the non-linear correlation between ESG performance and CE, contributing a new perspective to regional ESG performance studies. Secondly, we re-evaluate the role of green credit. Several academics have examined the facilitating effect of green credit on CE reduction, and Wu, et al.^20^ also pointed out the non-linear relationship between green credit and CE. Our research findings reveal that green credit not only influences CE growth rate as a threshold variable but also plays a negative moderating role between ESG performance and CE, expanding the scope of prior research. Thirdly, this paper concludes on the regional heterogeneity of the moderating effect of green credit, different from the findings of Guo, et al.^21^ regarding the differences in coastal areas. We believe that the moderating effect of green credit is greater in low ESG performance regions, exploring the moderating effect of green credit from diverse angles.

### Policy implications

Drawing from the empirical conclusions, the following policy recommendations are suggested by this study. Firstly, the government can transfer the responsibility of achieving carbon neutrality to enterprises through ESG performance evaluations. Each region needs to propose policies tailored to its own developmental characteristics based on the scores in the E, S, and G dimensions, focusing more on policies that directly optimize the environment and enhance administrative efficiency to reduce carbon emissions. The impact of the E dimension on CE is direct and rapid. For provinces with stringent environmental protection tasks, such as Hebei and Shanxi, which are heavily industrialized and suffer from severe air pollution, the government can impose higher requirements on enterprises from the E dimension to expedite and effectively promote carbon reduction.

Secondly, there is a need to vigorously develop green credit and leverage its role in promoting green innovation and its moderating effect on corporate ESG performance and carbon emissions. Local governments should establish reasonable ESG evaluation criteria to avoid the risk of "green washing" and enable commercial banks to allocate resources adequately for green credit. Only when green credit resources are directed towards companies with clear objectives in green technology and strong innovation capabilities can their intended effects be realized. Local governments and commercial banks should collaborate to formulate differentiated credit policies to ensure a more scientific allocation of green credit funds.

Thirdly, the government should pay more attention to providing green credit support in regions with low ESG performance, especially in economically underdeveloped areas, to channel financial resources to areas in greater need of funding. Economically underdeveloped regions often exhibit lower ESG performance, as their economic development heavily relies on environmental degradation and resource exploitation. By increasing green credit support in these regions, improvements in the E dimension of enterprises can be accelerated. Even in coastal areas, there are provinces with low ESG performance, such as Hebei, Liaoning and Hainan, which still require additional green credit resources to overcome the limitations of not meeting threshold values.

### Restrictions and upcoming studies

This study has various restrictions. Firstly, the sample is sourced from provinces in China, which are components of developing markets characterized by their distinct features differing from those of developed countries. Secondly, there are variations in existing ESG assessments. This study relies on unofficial ESG assessments institution, and despite conducting various robustness tests in the analysis, the outcomes could still be impacted. Additionally, not all listed companies participated in ESG disclosure, and listed companies only represent a portion of the provincial economy. Therefore, using the ESG performance of listed companies as a proxy for ESG performance in each province may introduce some errors.

Many suggestions for future research directions are made in light of the limitations of this study. First, in order to examine the stability of the experimental outcomes and draw more broadly applicable conclusions, we may broaden the sample of nations to include major economies like the US and Europe. Second, it would be possible to lessen errors related to dependent variable selection by combining assessment results from several ESG rating agencies and using a more sensible approach for comprehensive ESG evaluation. Thirdly, taking into account the use of alternative panel regression models for analysis and testing; for instance, using the Difference-in-Differences (DID) approach to compare the effects of CE before and after green credit policies are put into place (Supplementary Information).

## Supplementary Information

Supplementary Information 1.

Supplementary Information 2.

## Data Availability

Data is provided within the supplementary information files.

## References

[1] Allen, M. *et al.* Special report: Global warming of 1.5 °C. In *Intergovernmental Panel on Climate Change*. https://www.ipcc.ch/site/assets/uploads/2023/06/SR15_Citation.pdf (2018).

[2] Ding, J., Chen, W. & Fu, S. Optimal policy for remanufacturing firms with carbon options under service requirements. *J. Syst. Sci. Syst. Eng.* **31**, 34–63. 10.1007/s11518-021-5512-6 (2022).

[3] Arora, S., Sur, J. K. & Chauhan, Y. Does corporate social responsibility affect shareholder value? Evidence from the COVID-19 crisis. *Int. Rev. Financ.* **22**, 325–334. 10.1111/irfi.12353 (2022).

[4] Hepburn, C. *et al.* Towards carbon neutrality and China’s 14th Five-Year Plan: Clean energy transition, sustainable urban development, and investment priorities. *Environ. Sci. Ecotechnol.* **8**, 100130 (2021). 36156997 10.1016/j.ese.2021.100130 PMC9488078

[5] Doron, A., Cheng, S., Abraham, L. & Tarelli, A. Sustainable investing with ESG rating uncertainty. *J. Financ. Econ.*10.1016/j.jfineco.2021.09.009 (2021).

[6] Qiu, J. *2022 2nd International Conference on Economic Development and Business Culture (ICEDBC 2022).* 1827–1832. (Atlantis Press, 2022).

[7] Tuna, G., Türkay, K., Çiftyildiz, S. S. & Çelik, H. The impact of financial tools in environmental degradation management: The relationship between CO_2_ emission and ESG funds. *Environ. Dev. Sustain. *1–16 10.1007/s10668-023-03229-6 (2023). 10.1007/s10668-023-03229-6 PMC10092919 37363026

[8] Li, J. & Xu, X. Can ESG rating reduce corporate carbon emissions?—An empirical study from Chinese listed companies. *J. Clean. Prod.* **434**, 140226. 10.1016/j.jclepro.2023.140226 (2024).

[9] Lu, J. & Li, H. The impact of ESG ratings on low carbon investment: Evidence from renewable energy companies. *Renew. Energy* **223**, 119984. 10.1016/j.renene.2024.119984 (2024).

[10] Fan, M. & Ren, H. Research on corporate social responsibility of Chinese multinational enterprises based on embeddedness theory: A case study of Huawei. *Hum. Geogr.* **36**, 143–150 (2021) (**in Chinese**).

[11] Meng, B., Zhou, L. & Luo, J. Research on composite evaluation of corporate social responsibility in the transportation industry integrating difference and similarity dual constraints. *J. Syst. Eng. Theory Pract.* **33**, 3243–3258 (2020) (**in Chinese**).

[12] Luo, L. & Tang, Q. The real effects of ESG reporting and GRI standards on carbon mitigation: International evidence. *Bus. Strateg. Environ.* **32**, 2985–3000. 10.1002/bse.3281 (2023).

[13] Guo, X. & Wang, J. Outward foreign direct investment, green financial development, and green total factor productivity: evidence from China. *Environ. Sci. Pollut. Res. * 1–16. https://pubmed.ncbi.nlm.nih.gov/36746857/ (2023). 10.1007/s11356-023-25651-z 36746857

[14] Cong, Y., Zhu, C., Hou, Y., Tian, S. & Cai, X. Does ESG investment reduce carbon emissions in China?. *Front. Environ. Sci.* **10**, 977049. 10.3389/fenvs.2022.977049 (2022).

[15] Townsend, B. From SRI to ESG: The origins of socially responsible and sustainable investing. *J. Impact ESG Invest.* **1**, 10–25. 10.3905/jesg.2020.1.1.010 (2020).

[16] Aizawa, M. & Yang, C. Green credit, green stimulus, green revolution? China’s mobilization of banks for environmental cleanup. *J. Environ. Dev.* **19**, 119–144. 10.1177/1070496510371 (2010).

[17] Yang, C., Zhu, C. & Albitar, K. ESG ratings and green innovation: AU-shaped journey towards sustainable development. *Bus. Strateg. Environ.*10.1002/bse.3692 (2024).

[18] Liu, J., Duan, Y. & Zhong, S. Does green innovation suppress carbon emission intensity? New evidence from China. *Environ. Sci. Pollut. Res. ***29**, 86722–86743 10.1007/s11356-022-21621-z (2022). 10.1007/s11356-022-21621-z 35794333

[19] Ali, M., Azmi, W., Kowsalya, V. & Rizvi, S. A. R. Interlinkages between stability, carbon emissions and the ESG disclosures: Global evidence from banking industry. *Pac. Basin Financ. J.* **82**, 102154. 10.1016/j.pacfin.2023.102154 (2023).

[20] Wu, J., Yang, C. & Chen, L. Examining the non-linear effects of monetary policy on carbon emissions. *Energy Econ.* **131**, 107206. 10.1016/j.eneco.2023.107206 (2024).

[21] Guo, L., Tan, W., Xu, Y., Tang, Q. & Liu, G. Can green credit inhibit regional carbon emissions? Evidence from China. *Energy Environ*. 10.1177/0958305X231160592 (2023).

[22] Ye, J. & Xu, W. Carbon reduction effect of ESG: Empirical evidence from listed manufacturing companies in China. *Front. Ecol. Evolut. ***11**, 1311777 10.3389/fevo.2023.1311777 (2023).

[23] Senadheera, S. S. *et al.* Scoring environment pillar in environmental, social, and governance (ESG) assessment. *Environ. Manag. Conserv.* **7**, 1960097. 10.1080/27658511.2021.1960097 (2021).

[24] Zhou, J. Analysis and countermeasures of green finance development under carbon peaking and carbon neutrality goals. *Open J. Soc. Sci.* **10**, 147–154. 10.4236/jss.2022.102009 (2022).

[25] Shiyi, C. & Yu, Q. Study on medium-and long-term fiscal policies to cope with climate change under the “dual carbon” goals. *Front. Econ. China* **17** https://journal.hep.com.cn/fec/EN/abstract/article/1673-3444/34746 (2022).

[26] Zhou, J., Lei, X., Yu, J. J. B. S. & Environment, T. *ESG Rating Divergence and Corporate Green Innovation* (2023).

[27] Long, H., Feng, G. F., Gong, Q. & Chang, C. P. ESG performance and green innovation: An investigation based on quantile regression. *Bus. Strateg. Environ.* **32**, 5102–5118. 10.1002/bse.3410 (2023).

[28] Wang, J., Ma, M., Dong, T. & Zhang, Z. J. I. R. o. F. A. Do ESG ratings promote corporate green innovation? A quasi-natural experiment based on SynTao green finance's ESG ratings. *Int. Rev. Financ. Anal*. **87**, 102623 (2023).

[29] Lv, C., Fan, J. & Lee, C. C. J. J. o. C. P. Can green credit policies improve corporate green production efficiency? (2023).

[30] Su, D., Xu, S. & Tong, Z.J.P.-C.E. Credit availability and corporate risk-taking: Evidence from China’s green credit policy. *Post Commun. Econ.* **35**, 236–270 (2023).

[31] Hu, G., Wang, X. & Wang, Y. Can the green credit policy stimulate green innovation in heavily polluting enterprises? Evidence from a quasi-natural experiment in China. *Energy Econ.* **98**, 105134. 10.1016/j.eneco.2021.105134 (2021).

[32] Jiang, M., Qi, J. & Zhang, Z. Under the same roof? The green belt and road initiative and firms’ heterogeneous responses. *J. Appl. Econ.* **25**, 316–338. 10.1080/15140326.2022.2036566 (2022).

[33] Azmi, W., Hassan, M. K., Houston, R. & Karim, M. S. ESG activities and banking performance: International evidence from emerging economies. *J. Financ. Econ.* **70**, 101277. 10.1016/j.jfineco.2021.09.009 (2021).

[34] Rubashkina, Y., Galeotti, M. & Verdolini, E. Environmental regulation and competitiveness: Empirical evidence on the Porter hypothesis from European manufacturing sectors. *Energy Policy* **83**, 288–300. 10.1016/j.enpol.2015.02.014 (2015).

[35] Hart, S. L. J. Beyond greening: Strategies for a sustainable world. *Harvard Bus. Rev.* **75**, 66–77 https://go.gale.com/ps/i.do?id=GALE%7CA19129096&sid=googleScholar&v=2.1&it=r&linkaccess=abs&issn=00178012&p=AONE&sw=w&userGroupName=anon%7E60965029&aty=open+web+entry (1997).

[36] Lin, B. & Zhang, Z. Carbon emissions in China’s cement industry: A sector and policy analysis. *Sci. Total Environ.* **58**, 1387–1394. 10.1016/j.scitotenv.2020.137503 (2016).

[37] Baratta, A., Cimino, A., Longo, F., Solina, V. & Verteramo, S. The impact of ESG practices in industry with a focus on carbon emissions: Insights and future perspectives. *Sustainability* **15**, 6685. 10.3390/su15086685 (2023).

[38] Zhao, X., Xu, H., Yin, S. & Zhou, Y. Threshold effect of technological innovation on carbon emission intensity based on multi-source heterogeneous data. *Sci. Rep.* **13**, 19054. 10.1038/s41598-023-46406-3 (2023). 37925582 10.1038/s41598-023-46406-3 PMC10625548

[39] He, L., Zhang, L., Zhong, Z., Wang, D. & Wang, F. Green credit, renewable energy investment and green economy development: Empirical analysis based on 150 listed companies of China. *J. Clean. Prod.* **208**, 363–372. 10.1016/j.jclepro.2018.10.119 (2019).

[40] Zhao, X., Zeng, B., Zhao, X., Zeng, S. & Jiang, S. Impact of green finance on green energy efficiency: A pathway to sustainable development in China. *J. Clean. Product. * 141943 10.1016/j.jclepro.2024.141943 (2024).

[41] Liu, F., Jiang, J. & Zhang, S. Government environmental governance and enterprise coordinated green development under the goal of “double carbon”. *J. Environ. Public Health*10.1155/2022/6605935 (2022). 10.1155/2022/6605935 PMC9239759 35774193

[42] Huang, Z., Liao, G. & Li, Z. Loaning scale and government subsidy for promoting green innovation. *Technol. Forecast. Soc. Change* **144**, 148–156. 10.1016/j.techfore.2019.04.023 (2019).

[43] Gao, W. & Liu, Z. Green credit and corporate ESG performance: Evidence from China. *Finance Res. Lett.* **55**, 103940. 10.1016/j.frl.2023.103940 (2023).

[44] Chouaibi, S., Chouaibi, J. & Rossi, M. ESG and corporate financial performance: The mediating role of green innovation: UK common law versus Germany civil law. *EuroMed J. Bus.* **17**, 46–71. 10.1108/EMJB-09-2020-0101 (2022).

[45] Robertson, J. L., Montgomery, A. W. & Ozbilir, T. Employees’ response to corporate greenwashing. *Bus. Strategy Environ.*10.1002/bse.3351 (2023).

[46] Rajan, R. G. Has finance made the world riskier?. *Eur. Financ. Manag.* **12**, 499–533. 10.1111/j.1468-036X.2006.00330.x (2006).

[47] Sun, X., Zhou, C. & Gan, Z. Green finance policy and ESG performance: Evidence from Chinese manufacturing firms. *Sustainability* **15**, 6781. 10.3390/su15086781 (2023).

[48] Cheng, M., Wang, J., Yang, S. & Li, Q. The driving effect of technological innovation on green development: From the perspective of efficiency. *Energy Policy* **188**, 114089. 10.1016/j.enpol.2024.114089 (2024).

[49] Change, I. *2006 IPCC Guidelines for National Greenhouse Gas Inventories*. https://www.ipcc-nggip.iges.or.jp/meeting/pdfiles/Washington_Report.pdf (Institute for Global Environmental Strategies, 2006).

[50] Zhang, X. *The Relevance of Corporate Social Responsibility (CSR) and Corporate Financial Performance (CFP) and the Impact of the Chinese Government Recommending Companies to Disclose ESG Ratings Since 2016, Based on the Data of Chinese A-Share Companies* (2022).

[51] Song, M., Xie, Q. & Shen, Z. Impact of green credit on high-efficiency utilization of energy in China considering environmental constraints. *Energy Policy* **153**, 112267. 10.1016/j.enpol.2021.112267 (2021).

[52] Zhang, W., Li, G., Uddin, M. K. & Guo, S. Environmental regulation, foreign investment behavior, and carbon emissions for 30 provinces in China. *J. Clean. Prod.* **248**, 119208 (2020).

[53] Liu, Y., Lei, P., Shen, B. & He, D. Green technology advancement, energy input share and carbon emission trend studies. *Sci. Rep.* **14**, 2004. 10.1038/s41598-024-51790-5 (2024). 38263375 10.1038/s41598-024-51790-5 PMC10805776

[54] He, L., Yin, F., Zhong, Z. & Ding, Z. The impact of local government investment on the carbon emissions reduction effect: An empirical analysis of panel data from 30 provinces and municipalities in China. *Plos one* **12**, e0180946. 10.1371/journal.pone.0180946 (2017). 28727783 10.1371/journal.pone.0180946 PMC5519066

[55] Wang, Q. & Li, L. The effects of population aging, life expectancy, unemployment rate, population density, per capita GDP, urbanization on per capita carbon emissions. *Sustain. Prod. Consum.* **28**, 760–774. 10.1016/j.spc.2021.06.029 (2021).

[56] Yu, H. & Liu, H. Impact of digitization on carbon productivity: An empirical analysis of 136 countries. *Sci. Rep.* **14**, 5094. 10.1038/s41598-024-55848-2 (2024). 38429408 10.1038/s41598-024-55848-2 PMC10907719

[57] Akpan, G. E., Akpan, U. F. & Policy. Electricity consumption, carbon emissions and economic growth in Nigeria. *International Journal of Energy Economics* **2**, 292–306 (2012).

[58] Hansen, B. E. J. Threshold effects in non-dynamic panels: Estimation, testing, and inference. *J. Econ.* **93**, 345–368 10.1016/S0304-4076(99)00025-1 (1999).

[59] Zhao, J., Huang, J. & Liu, F. Green credit policy and investment-cash flow sensitivity: Evidence from a quasi-natural experiment. *Finance Res. Lett.* **52**, 103502. 10.1016/j.frl.2022.103502 (2023).

[60] Zhang, D. & Kong, Q. Credit policy, uncertainty, and firm R&D investment: A quasi-natural experiment based on the Green Credit Guidelines. *Pac.-Basin Finance J.* **73**, 101751. 10.1016/j.pacfin.2022.101751 (2022).

