# Supplementary material for: Research on the impact of ESG performance on carbon emissions from the perspective of green credit
Source: Sci Rep. 2024 May 7;14:10478. doi: 10.1038/s41598-024-61353-3 (PMC11636868; doi:10.1038/s41598-024-61353-3)
Supplement: Supplementary file 1 — Supplementary Information 1. [file 41598_2024_61353_MOESM1_ESM.pdf]

year	num	province	Ce	ESG	Gcredit	Regu	Gov	Pgdp	lnCe
2011	北京	11	12909.29	31.04633	0.476851	0.012398	0.188799	86246	9.465703
2012	北京	11	13116.49	32.57013	0.467063	0.018008	0.193712	92758	9.481626
2013	北京	11	12051.97	32.58555	0.47585	0.020511	0.19748	100569	9.396983
2014	北京	11	12487.95	34.41564	0.551638	0.027235	0.19736	106732	9.43252
2015	北京	11	12121.17	39.35825	0.729919	0.016647	0.231554	113692	9.402709
2016	北京	11	11472.5	41.13864	0.659294	0.024932	0.236926	123391	9.347708
2017	北京	11	11264.4	45.62422	0.712481	0.022267	0.228375	136172	9.329402
2018	北京	11	11540.56	48.10472	0.752882	0.01652	0.225682	150962	9.353623
2019	北京	11	11499.51	48.02316	0.808335	0.01627	0.209005	161776	9.35006
2020	北京	11	9765.136	48.28211	0.71574	0.014022	0.197983	164158	9.186574
2011	天津	12	20206.59	25.95905	0.412786	0.021559	0.221427	61458	9.913764
2012	天津	12	20352.44	28.17376	0.415228	0.017417	0.237002	66517	9.920957
2013	天津	12	21115.48	25.49965	0.456947	0.019235	0.256321	71345	9.957762
2014	天津	12	20394.04	26.15167	0.464555	0.026211	0.271103	74960	9.922998
2015	天津	12	20133.5	31.01809	0.478772	0.011618	0.297105	75868	9.91014
2016	天津	12	19009.2	35.68515	0.779496	0.004661	0.322329	79647	9.852678
2017	天津	12	18880.28	38.26561	0.691765	0.005719	0.263645	87280	9.845873
2018	天津	12	19597.91	39.96877	0.442349	0.005016	0.232222	95689	9.883178
2019	天津	12	19762.57	42.31132	0.585018	0.007665	0.252976	101557	9.891545
2020	天津	12	18986.82	43.3035	0.512037	0.009138	0.224915	101068	9.851501
2011	河北	13	91561.12	20.45949	0.440631	0.029175	0.165417	29647	11.42476
2012	河北	13	92850.74	20.38798	0.416092	0.021064	0.176771	31844	11.43875
2013	河北	13	93126.84	20.26812	0.414207	0.020198	0.181766	33346	11.44172
2014	河北	13	88574.24	20.82961	0.444327	0.018069	0.185542	34507	11.3916
2015	河北	13	92618.08	26.51481	0.452204	0.015058	0.213353	35994	11.43624
2016	河北	13	92750.14	29.67254	0.451838	0.014034	0.212457	38688	11.43766
2017	河北	13	92078.21	31.9243	0.462903	0.019771	0.216678	41451	11.43039
2018	河北	13	94441.68	34.23892	0.472708	0.014521	0.237769	43808	11.45574
2019	河北	13	94794.99	35.76945	0.462186	0.013788	0.237546	47036	11.45947
2020	河北	13	94090.33	38.63048	0.385202	0.016716	0.250508	48302	11.45201
2011	山西	14	73861.34	24.89194	0.477723	0.02281	0.216978	30534	11.20994
2012	山西	14	77128.29	24.3903	0.51053	0.028092	0.236192	32864	11.25323
2013	山西	14	79094.89	24.91475	0.575564	0.02813	0.25278	33848	11.2784
2014	山西	14	80993.41	24.47786	0.59736	0.024242	0.255094	34248	11.30212
2015	山西	14	92972.77	31.88017	0.621194	0.021763	0.28919	33593	11.44006
2016	山西	14	92016.69	31.45367	0.660005	0.044005	0.28702	33972	11.42973
2017	山西	14	96965.04	31.94807	0.656611	0.019207	0.259344	41242	11.48211
2018	山西	14	103795.3	31.87997	0.653734	0.014952	0.268447	45517	11.55018
2019	山西	14	109189	32.80567	0.619159	0.021016	0.277731	48469	11.60083
2020	山西	14	113907.7	33.82757	0.691752	0.011662	0.286559	51051	11.64314
2011	内蒙古	15	75483.64	16.98309	0.334736	0.041858	0.316048	38276	11.23167
2012	内蒙古	15	78432.42	17.61407	0.357378	0.042512	0.327217	42441	11.26999
2013	内蒙古	15	76626.69	17.03947	0.350217	0.044486	0.323595	46320	11.2467
2014	内蒙古	15	78512.77	18.20172	0.306651	0.046249	0.319125	49585	11.27102
2015	内蒙古	15	77870.64	28.77489	0.30373	0.041424	0.328439	52972	11.2628
2016	内蒙古	15	78787.98	30.10327	0.306368	0.033069	0.327262	56560	11.27452
2017	内蒙古	15	82898.92	32.55404	0.318833	0.028165	0.304061	61196	11.32538
2018	内蒙古	15	95076.16	34.41964	0.32977	0.014617	0.299332	66491	11.46243
2019	内蒙古	15	105466.6	34.66174	0.323177	0.015125	0.296349	71170	11.56615
2020	内蒙古	15	111450.1	36.24807	0.314969	0.052034	0.305262	71640	11.62133
2011	辽宁	21	71902.09	20.42814	0.44432	0.023021	0.238818	37353	11.18306
2012	辽宁	21	74514.39	21.03436	0.443039	0.038289	0.255403	40778	11.21875
2013	辽宁	21	71833.56	20.00995	0.464453	0.018096	0.270575	43956	11.18211

2014	辽宁	21	71939.39	20.80323	0.449938	0.013558	0.253699	45915	11.18358
2015	辽宁	21	69460.3	28.21447	0.449247	0.014404	0.221749	46482	11.14851
2016	辽宁	21	70356.09	29.7384	0.456528	0.00864	0.224468	47069	11.16132
2017	辽宁	21	72481.9	34.23093	0.43389	0.010105	0.224931	50221	11.19109
2018	辽宁	21	77082.24	34.89718	0.412202	0.0065	0.227036	54657	11.25263
2019	辽宁	21	83962.42	34.48671	0.410739	0.005004	0.231141	58019	11.33812
2020	辽宁	21	85718.43	34.66578	0.418289	0.006717	0.23997	58629	11.35882
2011	吉林	22	28718.64	19.55568	0.548236	0.013084	0.284661	28270	10.2653
2012	吉林	22	28380.13	19.35681	0.570996	0.011915	0.284766	32005	10.25344
2013	吉林	22	27346.67	20.73695	0.553305	0.01118	0.291137	35139	10.21635
2014	吉林	22	27124.29	22.04902	0.519034	0.009843	0.292304	37539	10.20819
2015	吉林	22	23154.83	26.62464	0.524289	0.01106	0.321132	38128	10.04996
2016	吉林	22	22830.4	26.76557	0.530255	0.008066	0.343923	40259	10.03585
2017	吉林	22	22654.03	27.92851	0.496136	0.008387	0.341121	42890	10.02809
2018	吉林	22	23438.97	28.39998	0.450353	0.007385	0.336739	44925	10.06216
2019	吉林	22	24145.83	27.05974	0.577754	0.006354	0.335421	47554	10.09187
2020	吉林	22	23500.2	34.98208	0.546234	0.013871	0.336747	50561	10.06476
2011	黑龙江	23	36760.07	19.70437	0.53704	0.01537	0.281236	26093	10.51217
2012	黑龙江	23	38506.59	19.29171	0.529513	0.019799	0.287906	29352	10.55858
2013	黑龙江	23	36540.23	18.17883	0.529474	0.025192	0.284341	32068	10.50617
2014	黑龙江	23	37035.53	18.33415	0.547808	0.014962	0.282169	33464	10.51963
2015	黑龙江	23	33896.89	24.07909	0.547442	0.013422	0.34394	32759	10.43108
2016	黑龙江	23	34181.15	24.65312	0.540732	0.014594	0.355388	34025	10.43943
2017	黑龙江	23	34205.06	27.98042	0.527485	0.010672	0.376925	35887	10.44013
2018	黑龙江	23	34887.06	28.14422	0.514305	0.008139	0.364049	38199	10.45987
2019	黑龙江	23	36624.43	29.63513	0.649224	0.007096	0.37001	41156	10.50847
2020	黑龙江	23	36692.24	31.93721	0.600715	0.019658	0.399709	42432	10.51032
2011	上海	31	27745.28	28.45211	0.613134	0.007236	0.195649	85897	10.23082
2012	上海	31	27354.02	29.51431	0.654578	0.006294	0.196381	89613	10.21662
2013	上海	31	29014.43	31.18096	0.663589	0.008085	0.195164	95746	10.27555
2014	上海	31	26419.36	31.00317	0.715495	0.009893	0.194835	102827	10.18185
2015	上海	31	26527.39	37.87473	0.695731	0.008194	0.230281	109186	10.18593
2016	上海	31	26434.04	38.48204	0.66835	0.006869	0.231503	121369	10.18241
2017	上海	31	26929.46	39.87165	0.670236	0.004872	0.229237	133489	10.20098
2018	上海	31	26511.64	40.90444	0.672074	0.005881	0.231911	145767	10.18534
2019	上海	31	27363.15	41.47189	0.666381	0.004889	0.215314	153299	10.21695
2020	上海	31	25906.97	42.65169	0.672356	0.005441	0.207942	156803	10.16227
2011	江苏	32	77430.4	21.85603	0.603467	0.01179	0.127392	61464	11.25713
2012	江苏	32	79059.51	22.38859	0.605554	0.012236	0.130864	66533	11.27796
2013	江苏	32	81293.88	22.40454	0.618932	0.014844	0.131399	72768	11.30583
2014	江苏	32	80721.12	22.69318	0.629714	0.013583	0.130686	78711	11.29876
2015	江苏	32	83601.64	28.92629	0.648877	0.013367	0.135955	85871	11.33382
2016	江苏	32	87016	29.89101	0.646122	0.009898	0.129048	92658	11.37385
2017	江苏	32	86199.77	32.20126	0.654266	0.008331	0.123688	102202	11.36442
2018	江苏	32	85430.73	33.62152	0.662884	0.007277	0.125069	110508	11.35546
2019	江苏	32	87262.49	34.74647	0.664273	0.006969	0.127447	116650	11.37668
2020	江苏	32	83850.76	37.0974	0.661672	0.006536	0.133088	121333	11.33679
2011	浙江	33	45514.02	19.62803	0.722579	0.007493	0.120628	57828	10.72578
2012	浙江	33	44156.25	19.97973	0.725123	0.010918	0.121047	61097	10.69549
2013	浙江	33	45417.89	19.68301	0.722793	0.010457	0.126705	65105	10.72366
2014	浙江	33	44897.49	19.80385	0.707667	0.011848	0.128914	68569	10.71214
2015	浙江	33	45527.35	28.05055	0.703514	0.010106	0.152754	73276	10.72607
2016	浙江	33	45187.5	28.41485	0.69734	0.013768	0.147591	78384	10.71858
2017	浙江	33	47325.61	31.10857	0.698946	0.008643	0.1437	85612	10.76481

2018	浙江	33	46417.54	33.42324	0.700638	0.008274	0.148778	93230	10.74543
2019	浙江	33	47416.69	34.34184	0.71098	0.006873	0.160946	98770	10.76673
2020	浙江	33	52831.99	35.50436	0.697864	0.009275	0.155851	100738	10.87487
2011	安徽	34	33674.33	21.19126	0.582797	0.016426	0.202825	27303	10.42449
2012	安徽	34	35194.42	22.19168	0.616424	0.018003	0.215957	30697	10.46864
2013	安徽	34	38223.74	24.18463	0.615194	0.024582	0.211314	34404	10.55121
2014	安徽	34	39491.66	23.88399	0.643932	0.019037	0.207112	37580	10.58385
2015	安徽	34	39570.02	28.73435	0.665611	0.018451	0.219838	39692	10.58583
2016	安徽	34	39503.56	32.06954	0.684667	0.018937	0.209937	43686	10.58415
2017	安徽	34	40886.62	34.56242	0.679408	0.017017	0.20905	49092	10.61856
2018	安徽	34	42451.12	36.67782	0.673956	0.011666	0.193237	56063	10.65611
2019	安徽	34	42725.48	40.05962	0.612838	0.013586	0.200627	60561	10.66255
2020	安徽	34	43789.98	40.30052	0.580427	0.012926	0.196287	62411	10.68716
2011	福建	35	25906.23	20.61668	0.558716	0.011073	0.122682	47928	10.16224
2012	福建	35	25707.56	21.31565	0.564958	0.01102	0.129144	52959	10.15454
2013	福建	35	25241.8	23.70382	0.559271	0.012571	0.136368	58255	10.13626
2014	福建	35	28798.11	23.56975	0.552437	0.007754	0.132575	63709	10.26807
2015	福建	35	27692.93	30.84838	0.540063	0.008565	0.149204	67649	10.22893
2016	福建	35	25985.42	31.95341	0.541662	0.006403	0.144393	74024	10.16529
2017	福建	35	27377.52	32.8411	0.556624	0.006631	0.138411	83758	10.21748
2018	福建	35	30298.35	33.57979	0.571205	0.008177	0.124915	94719	10.31885
2019	福建	35	32248.79	36.44145	0.550538	0.007521	0.11997	102722	10.38124
2020	福建	35	32174.74	41.91149	0.520198	0.012612	0.119578	105106	10.37894
2011	江西	36	19081.63	20.58542	0.382008	0.020821	0.218792	25928	9.856482
2012	江西	36	19184.95	19.8726	0.369426	0.02468	0.235735	28624	9.861881
2013	江西	36	20671.7	27.17082	0.410305	0.016755	0.242675	31952	9.936521
2014	江西	36	21055.2	27.81328	0.417862	0.014756	0.247814	34988	9.954903
2015	江西	36	21942.15	30.01657	0.468601	0.014034	0.262951	37436	9.996164
2016	江西	36	22231.93	34.10847	0.502005	0.017038	0.251101	40950	10.00928
2017	江西	36	22700.38	37.47918	0.510832	0.01561	0.252908	44878	10.03014
2018	江西	36	23728.62	39.14959	0.51849	0.015814	0.249489	50347	10.07444
2019	江西	36	24236.1	42.98164	0.597582	0.018076	0.258918	54640	10.0956
2020	江西	36	23700.73	44.186	0.579345	0.011714	0.258556	57065	10.07326
2011	山东	37	114469.5	22.43237	0.574536	0.01572	0.128045	40581	11.64806
2012	山东	37	120484.2	22.69001	0.596242	0.017205	0.137451	44348	11.69927
2013	山东	37	117307.8	24.09385	0.60676	0.017911	0.14128	48673	11.67256
2014	山东	37	125648.3	24.02223	0.603953	0.016225	0.141356	51933	11.74124
2015	山东	37	138350.2	31.73519	0.606039	0.012538	0.149217	56205	11.83754
2016	山东	37	145358.7	33.11166	0.627479	0.013287	0.148993	59239	11.88696
2017	山东	37	149157.4	35.44837	0.580697	0.015057	0.146931	62993	11.91276
2018	山东	37	147672.3	37.62767	0.529834	0.013878	0.151555	66284	11.90275
2019	山东	37	151523.5	39.91774	0.496531	0.009911	0.15225	69901	11.9285
2020	山东	37	148788.1	41.93057	0.448881	0.009863	0.154278	71825	11.91028
2011	河南	41	67005.12	21.3015	0.49607	0.006205	0.161437	27901	11.11252
2012	河南	41	62704.93	20.77233	0.479051	0.007234	0.172862	30497	11.0462
2013	河南	41	62232.42	20.98649	0.490286	0.009108	0.176474	33114	11.03863
2014	河南	41	62908.51	21.09486	0.501178	0.008535	0.174367	35982	11.04944
2015	河南	41	59238.5	28.80456	0.530064	0.007976	0.183349	38338	10.98933
2016	河南	41	58551.87	30.53268	0.55346	0.008939	0.185189	41326	10.97767
2017	河南	41	57306.92	34.81243	0.542209	0.014307	0.18328	45723	10.95618
2018	河南	41	57644.35	38.34784	0.530425	0.000591	0.184591	50714	10.96205
2019	河南	41	53330.08	41.83685	0.516806	0.000154	0.18921	54356	10.88426
2020	河南	41	53586.8	43.57473	0.48849	0.000111	0.191354	54691	10.88906
2011	湖北	42	41094.94	21.58183	0.388994	0.013027	0.1612	34719	10.62364

2012	湖北	42	41120.34	20.0706	0.42727	0.012638	0.166429	39149	10.62426
2013	湖北	42	35789.83	19.38245	0.473856	0.009957	0.172261	43835	10.48542
2014	湖北	42	36236.76	20.79077	0.482584	0.011207	0.174709	48635	10.49783
2015	湖北	42	34400.63	27.83845	0.495843	0.008133	0.20211	52021	10.44583
2016	湖北	42	34214.53	28.64802	0.527389	0.013933	0.192576	56844	10.44041
2017	湖北	42	35017.54	29.18044	0.544532	0.011669	0.182658	63169	10.4636
2018	湖北	42	36411.69	31.82151	0.5634	0.012568	0.172725	71097	10.50265
2019	湖北	42	38661.37	32.17008	0.604004	0.012732	0.175443	76712	10.5626
2020	湖北	42	34576.16	35.80116	0.578838	0.012464	0.196236	73687	10.45092
2011	湖南	43	32713.85	26.0205	0.440385	0.00673	0.186136	28766	10.39555
2012	湖南	43	32224.21	23.53791	0.489752	0.008973	0.194226	32203	10.38047
2013	湖南	43	31342.7	24.36724	0.48318	0.009934	0.199229	35702	10.35274
2014	湖南	43	30432.25	21.64227	0.519346	0.008257	0.193861	39181	10.32326
2015	湖南	43	30310.6	25.45086	0.532266	0.018838	0.200736	43155	10.31925
2016	湖南	43	30998.18	27.01562	0.5446	0.006495	0.20546	46606	10.34168
2017	湖南	43	31298.34	29.73167	0.570287	0.006483	0.203068	51030	10.35132
2018	湖南	43	31996.62	32.60448	0.598308	0.009962	0.205881	54763	10.37339
2019	湖南	43	31906.85	34.21757	0.581688	0.010304	0.201394	60104	10.37058
2020	湖南	43	31031.86	37.43594	0.55437	0.010399	0.202267	62537	10.34277
2011	广东	44	62830.44	28.86821	0.568451	0.006267	0.126475	50076	11.04819
2012	广东	44	61789.93	29.74172	0.590914	0.004564	0.129594	52308	11.0315
2013	广东	44	62873.12	29.42758	0.600996	0.00563	0.134569	56029	11.04887
2014	广东	44	63321.75	29.60691	0.611516	0.004446	0.134256	59909	11.05598
2015	广东	44	63915.4	35.92334	0.628584	0.003915	0.17165	64516	11.06532
2016	广东	44	66030.88	38.17993	0.650208	0.004473	0.163651	69671	11.09788
2017	广东	44	69115.01	41.11889	0.673508	0.003995	0.164077	76218	11.14353
2018	广东	44	71407.49	43.42446	0.693455	0.001529	0.157379	81625	11.17616
2019	广东	44	70888.05	44.3994	0.684704	0.001351	0.160185	86956	11.16886
2020	广东	44	71772.26	45.6003	0.678318	0.001925	0.157305	88521	11.18125
2011	广西	45	21146.83	21.21949	0.380563	0.01568	0.247117	22234	9.959245
2012	广西	45	23225.42	23.0652	0.413347	0.016853	0.264096	24181	10.053
2013	广西	45	23350.9	23.54881	0.427723	0.017496	0.257758	26416	10.05839
2014	广西	45	23179.88	24.5403	0.44464	0.014749	0.256097	28603	10.05104
2015	广西	45	21763.64	29.38811	0.445616	0.017651	0.274737	30890	9.987996
2016	广西	45	22626.27	30.92735	0.453254	0.01267	0.275598	33340	10.02687
2017	广西	45	23934.53	33.82063	0.447597	0.010348	0.275905	36441	10.08308
2018	广西	45	25149.44	33.32699	0.441629	0.015844	0.270572	39837	10.13259
2019	广西	45	26594.69	33.91641	0.416722	0.02318	0.275507	42778	10.18847
2020	广西	45	27330.22	35.40048	0.376638	0.037611	0.278263	44237	10.21575
2011	海南	46	6383.839	18.57841	0.550216	0.011369	0.316097	28014	8.761525
2012	海南	46	6653.508	18.46473	0.495627	0.016025	0.326834	30993	8.802899
2013	海南	46	6182.041	18.21056	0.511428	0.008537	0.324519	34053	8.729403
2014	海南	46	6839.469	21.58195	0.674656	0.006118	0.318858	37166	8.830465
2015	海南	46	7487.563	31.42441	0.485758	0.005945	0.331913	39704	8.920999
2016	海南	46	7267.54	31.23754	0.400467	0.007408	0.336531	43009	8.891173
2017	海南	46	7059.555	32.8041	0.403956	0.012029	0.321061	46631	8.862137
2018	海南	46	7470.716	30.07088	0.399868	0.032757	0.344411	50263	8.918746
2019	海南	46	7673.781	30.02015	0.401861	0.041064	0.348653	53929	8.945564
2020	海南	46	7552.315	30.04644	0.382302	0.069347	0.354621	55438	8.929609
2011	重庆	50	17922.84	22.59428	0.530911	0.025509	0.252947	34864	9.793831
2012	重庆	50	17689.98	20.847	0.477338	0.016118	0.262721	39180	9.780753
2013	重庆	50	15321.63	19.79028	0.511514	0.013303	0.235061	43527	9.637021
2014	重庆	50	16420.49	22.05289	0.504114	0.011515	0.22596	48311	9.706285
2015	重庆	50	14401.42	28.27187	0.528519	0.008666	0.236402	52480	9.575082

2016	重庆	50	14842.37	29.9889	0.559122	0.008001	0.222039	58327	9.605241
2017	重庆	50	15301.33	31.73391	0.570518	0.011068	0.216098	64171	9.635695
2018	重庆	50	15402.1	33.47594	0.583529	0.001739	0.210338	68460	9.642259
2019	重庆	50	15544.32	34.4274	0.571524	0.009766	0.20536	74337	9.65145
2020	重庆	50	15449.54	34.73996	0.579152	0.002716	0.195434	78294	9.645334
2011	四川	51	34858.73	21.01263	0.465796	0.006655	0.222077	26136	10.45906
2012	四川	51	36298.63	20.91847	0.495504	0.007453	0.227861	29627	10.49954
2013	四川	51	37279.69	22.54003	0.486901	0.008824	0.234592	32750	10.5262
2014	四川	51	38602.21	22.38207	0.407007	0.009975	0.235248	35563	10.56106
2015	四川	51	33061.23	27.5923	0.450918	0.007119	0.2471	37150	10.40612
2016	四川	51	32509.91	29.5484	0.446401	0.008763	0.241679	40297	10.3893
2017	四川	51	31921.65	33.34741	0.454597	0.008131	0.229382	45835	10.37104
2018	四川	51	31129.39	33.54186	0.463086	0.004124	0.226271	51658	10.34591
2019	四川	51	33418.36	34.7066	0.461468	0.004292	0.223195	55619	10.41686
2020	四川	51	32214.92	36.11779	0.454511	0.005196	0.230935	58009	10.38019
2011	贵州	52	25672.96	22.61421	0.248074	0.011557	0.400563	16024	10.15319
2012	贵州	52	28092.7	20.39071	0.262977	0.010219	0.408721	18947	10.24327
2013	贵州	52	29167.2	21.76089	0.291595	0.013759	0.386633	22089	10.2808
2014	贵州	52	28170.34	15.75835	0.303248	0.018576	0.386216	25101	10.24603
2015	贵州	52	28192.9	24.35322	0.290817	0.013044	0.373731	28547	10.24683
2016	贵州	52	29533.47	26.02723	0.314485	0.01004	0.36145	31589	10.29328
2017	贵州	52	29703.17	29.10434	0.304048	0.015928	0.339021	35988	10.29901
2018	贵州	52	27375	29.38254	0.292967	0.024272	0.327598	40271	10.21739
2019	贵州	52	28048.45	29.38267	0.28755	0.021057	0.35474	43727	10.24169
2020	贵州	52	27211.74	30.6651	0.275462	0.023292	0.320445	46355	10.2114
2011	云南	53	24723.26	20.86676	0.261155	0.012517	0.307631	20653	10.1155
2012	云南	53	25674.32	20.77823	0.223089	0.011931	0.321937	23992	10.15325
2013	云南	53	25403.47	23.03107	0.214774	0.015368	0.319404	27665	10.14264
2014	云南	53	22841.97	20.73803	0.20079	0.010825	0.316057	30217	10.03636
2015	云南	53	20683.86	31.37875	0.182523	0.009412	0.315029	32117	9.937109
2016	云南	53	20459.01	34.7836	0.195782	0.008907	0.306608	35051	9.926179
2017	云南	53	21731.59	35.67584	0.206108	0.007714	0.309043	39458	9.986522
2018	云南	53	24154.15	35.23375	0.217079	0.007447	0.290941	44446	10.09221
2019	云南	53	25263.71	35.67341	0.206882	0.011784	0.291515	49323	10.13712
2020	云南	53	26506.55	38.53829	0.202231	0.006434	0.284008	52047	10.18515
2011	陕西	61	37882.65	24.23197	0.464528	0.012591	0.240722	32467	10.54225
2012	陕西	61	43498.86	23.17183	0.47087	0.01277	0.235024	37453	10.68049
2013	陕西	61	46207.15	19.37112	0.496437	0.013939	0.230429	41906	10.74089
2014	陕西	61	48683.98	22.9899	0.498946	0.0164	0.227697	45610	10.79311
2015	陕西	61	48203.63	27.91559	0.516269	0.013431	0.244489	46654	10.78319
2016	陕西	61	49098.38	30.25098	0.477723	0.016665	0.230464	49341	10.80158
2017	陕西	61	50603.14	31.59434	0.471691	0.014646	0.225077	55216	10.83177
2018	陕西	61	49511.59	34.17314	0.466066	0.00795	0.221471	61115	10.80996
2019	陕西	61	53770.63	35.68491	0.48019	0.010223	0.221707	65506	10.89248
2020	陕西	61	55166.76	38.83425	0.433516	0.009918	0.228099	65867	10.91812
2011	甘肃	62	19883.68	20.81411	0.154562	0.012373	0.371866	18846	9.897655
2012	甘肃	62	20471.86	18.72428	0.170275	0.02251	0.381888	21141	9.926806
2013	甘肃	62	21170.5	18.7687	0.194144	0.029296	0.384009	23647	9.960363
2014	甘肃	62	21321.31	18.70109	0.205876	0.022015	0.389895	25724	9.967463
2015	甘肃	62	20622.95	27.44734	0.182509	0.018699	0.451196	25946	9.934159
2016	甘肃	62	19838.02	27.72438	0.215697	0.017024	0.456004	27396	9.895355
2017	甘肃	62	20013.21	27.52919	0.214487	0.012172	0.450399	29103	9.904148
2018	甘肃	62	20966.05	30.83466	0.213536	0.093621	0.465472	32178	9.95066
2019	甘肃	62	21227.02	31.31334	0.242235	0.014259	0.453254	34707	9.96303

2020	甘肃	62	22722.69	31.45731	0.237346	0.015368	0.462699	35848	10.03112
2011	青海	63	4879.879	19.48298	0.112446	0.019119	0.705976	24233	8.492876
2012	青海	63	5817.49	20.32865	0.137435	0.015767	0.758292	26839	8.668624
2013	青海	63	6406.414	19.9318	0.130741	0.021421	0.716775	30005	8.765055
2014	青海	63	5978.305	19.16397	0.125484	0.016236	0.729247	32218	8.695892
2015	青海	63	5518.908	24.95881	0.096026	0.017355	0.753436	34883	8.615935
2016	青海	63	6427.82	28.15936	0.093954	0.024931	0.675228	38968	8.768391
2017	青海	63	6159.567	29.15443	0.117829	0.016673	0.620843	42211	8.725761
2018	青海	63	6024.936	32.19964	0.140576	0.007805	0.599501	46854	8.703662
2019	青海	63	5955.566	36.11255	0.141699	0.009581	0.633664	49976	8.692081
2020	青海	63	5503.426	36.99139	0.181921	0.004651	0.642328	50845	8.613126
2011	宁夏	64	17321.93	20.33353	0.292702	0.029713	0.365416	30161	9.759728
2012	宁夏	64	18611.17	19.71092	0.325933	0.026138	0.405612	32609	9.831517
2013	宁夏	64	19805.06	19.68575	0.336432	0.031104	0.396305	35135	9.893693
2014	宁夏	64	20159.58	19.73117	0.360847	0.031772	0.404402	36815	9.911435
2015	宁夏	64	20931.56	26.03248	0.390987	0.03369	0.441378	37876	9.949014
2016	宁夏	64	20834.92	26.67582	0.314194	0.036385	0.451046	40339	9.944386
2017	宁夏	64	25612.94	27.96987	0.28922	0.026373	0.428954	45718	10.15085
2018	宁夏	64	28548.7	29.31344	0.26958	0.01833	0.404268	49614	10.25937
2019	宁夏	64	31028.75	44.86663	0.274068	0.020334	0.383697	52537	10.34267
2020	宁夏	64	33988.62	48.03223	0.267843	0.018704	0.374848	55021	10.43378
2011	新疆	65	32674.21	22.68461	0.419977	0.020315	0.349738	29624	10.39434
2012	新疆	65	37746.55	22.5986	0.42169	0.034418	0.366992	33103	10.53865
2013	新疆	65	43225.16	22.82547	0.392964	0.037974	0.365455	36988	10.67418
2014	新疆	65	48049.8	22.85302	0.370936	0.042355	0.358119	40193	10.77999
2015	新疆	65	49645.8	29.1048	0.328054	0.03102	0.408822	39520	10.81267
2016	新疆	65	51804.14	30.4842	0.327782	0.032479	0.429689	40020	10.85523
2017	新疆	65	55101.28	31.68532	0.299157	0.034543	0.415527	45476	10.91693
2018	新疆	65	57360.59	32.32525	0.270527	0.011721	0.39131	51238	10.95711
2019	新疆	65	61001.25	34.20379	0.799924	0.013774	0.390928	53542	11.01865
2020	新疆	65	65337.69	36.71811	0.300048	0.011159	0.395179	53606	11.08732

lnESG	lnGcredit	lnRegu	lnGov	lnPgdp	Popu	Ele	lnEle	E	S
3.435481	-0.74055	-4.39025	-1.66707	11.36496	1233	821.71	6.711388	6.716488	14.42593
3.483396	-0.76129	-4.01693	-1.64138	11.43775	1266	874.3	6.773424	6.472127	16.69515
3.483869	-0.74265	-3.88678	-1.62212	11.5186	1295	913.1	6.816845	7.121222	17.1531
3.538511	-0.59486	-3.60324	-1.62273	11.57808	1323	937.05	6.842737	9.180936	18.64476
3.672706	-0.31482	-4.09552	-1.46294	11.64125	1333	952.72	6.859321	9.356126	18.79748
3.716948	-0.41659	-3.69159	-1.44001	11.72311	1337	1020.27	6.927823	11.27914	19.10742
3.820439	-0.339	-3.80466	-1.47677	11.82167	1337	1066.89	6.972503	16.46211	20.53437
3.87338	-0.28385	-4.10317	-1.48863	11.92478	1336	1142.38	7.040869	19.51133	21.8662
3.871683	-0.21278	-4.11846	-1.5654	11.99397	1334	1166	7.061334	20.38172	21.90285
3.877061	-0.33444	-4.26712	-1.61957	12.00858	1334	1140	7.038784	21.34654	24.82709
3.25652	-0.88483	-3.83695	-1.50766	11.02611	1127	695.15	6.544127	3.794044	10.86208
3.338391	-0.87893	-4.05032	-1.43969	11.10521	1158	722.5	6.582717	4.461349	11.85495
3.238665	-0.78319	-3.95102	-1.36133	11.17528	1185	774.5	6.652218	3.171445	12.45502
3.263913	-0.76668	-3.64158	-1.30526	11.22471	1201	794.36	6.677537	4.54653	14.43494
3.434571	-0.73653	-4.45518	-1.21367	11.23675	1209	800.6	6.685361	3.979424	14.63228
3.574734	-0.24911	-5.36844	-1.13218	11.28536	1212	807.93	6.694475	5.504372	16.65828
3.644552	-0.36851	-5.16403	-1.33315	11.37688	1185	805.59	6.691575	10.33218	17.13496
3.688098	-0.81566	-5.29519	-1.46006	11.46886	1162	855.14	6.751265	12.55599	16.72902
3.745054	-0.53611	-4.87112	-1.37446	11.52838	1164	878	6.777647	15.83015	18.35194
3.768233	-0.66936	-4.69535	-1.49203	11.52355	1165	875	6.774224	18.54025	19.52654
3.018447	-0.81955	-3.53444	-1.79929	10.29712	386	2984.9	8.001322	1.922803	7.854095
3.014945	-0.87685	-3.8602	-1.7329	10.3686	388	3077.7	8.031938	2.393115	10.56298
3.009049	-0.88139	-3.90216	-1.70503	10.41469	389	3251.2	8.08678	2.611779	10.5111
3.036376	-0.8112	-4.01356	-1.68448	10.44892	391	3314.11	8.105945	3.283428	10.69749
3.277704	-0.79362	-4.19586	-1.54481	10.49111	392	3175.66	8.063271	3.676184	11.04582
3.390222	-0.79443	-4.26629	-1.54901	10.56328	394	3264.52	8.090868	6.007152	11.5648
3.463367	-0.77024	-3.92354	-1.52934	10.63227	396	3441.74	8.143732	8.963423	12.47795
3.533363	-0.74928	-4.23217	-1.43646	10.68757	397	3665.66	8.206763	14.35215	12.79329
3.577094	-0.77179	-4.28393	-1.43739	10.75867	398	3856	8.257385	17.37752	13.86218
3.654042	-0.95399	-4.0914	-1.38427	10.78523	399	3934	8.277412	20.19017	17.92653
3.214544	-0.73872	-3.78056	-1.52796	10.3266	227	1650.41	7.408779	6.448334	9.886231
3.194186	-0.67231	-3.57228	-1.44311	10.40013	226	1765.8	7.476359	8.046597	11.06822
3.21546	-0.55241	-3.57092	-1.37523	10.42964	226	1832.3	7.513327	7.531548	11.69963
3.197769	-0.51524	-3.71967	-1.36613	10.44138	225	1822.63	7.508036	8.097241	10.85985
3.461984	-0.47611	-3.82753	-1.24067	10.42207	225	1737.21	7.460036	7.837296	11.25699
3.448516	-0.41551	-3.12346	-1.2482	10.43329	224	1797.18	7.493974	7.600668	11.27965
3.464112	-0.42066	-3.95248	-1.3496	10.62721	224	1990.61	7.596196	7.723363	11.02934
3.461978	-0.42505	-4.20291	-1.3151	10.72584	223	2160.53	7.678109	8.322307	11.107
3.490601	-0.47939	-3.86247	-1.2811	10.78868	223	2262	7.724005	11.48951	11.31799
3.521276	-0.36853	-4.45141	-1.24981	10.84058	223	2342	7.75876	13.61789	12.60904
2.832218	-1.09441	-3.17347	-1.15186	10.55258	21	1864.07	7.530518	1.22346	6.048914
2.868698	-1.02896	-3.15798	-1.11713	10.65587	21	2016.8	7.609267	1.315339	8.211751
2.835533	-1.0492	-3.11259	-1.12826	10.74333	21	2181.9	7.687951	1.279284	8.270524
2.901516	-1.18205	-3.07372	-1.14217	10.81144	20	2416.74	7.790175	1.53206	9.332234
3.359503	-1.19162	-3.18389	-1.1134	10.87752	20	2542.87	7.841049	2.641243	10.43289
3.404634	-1.18297	-3.40916	-1.117	10.94306	20	2605.03	7.8652	3.102777	11.67277
3.482902	-1.14309	-3.56969	-1.19053	11.02184	20	2891.87	7.969659	6.023571	12.19406
3.538627	-1.10936	-4.22559	-1.2062	11.10482	20	3353.44	8.117742	10.96324	11.60994
3.545636	-1.12956	-4.19144	-1.21622	11.17283	20	3653	8.203304	12.00893	11.27359
3.590386	-1.15528	-2.95586	-1.18658	11.17941	20	3900	8.268732	12.27078	12.81917
3.016913	-0.81121	-3.77137	-1.43205	10.52817	296	1861.53	7.529154	2.712697	6.512461
3.046157	-0.8141	-3.2626	-1.36491	10.6159	295	1899.9	7.549557	2.957592	8.105831
2.99623	-0.76689	-4.01207	-1.30721	10.69094	295	2008.5	7.605144	2.515109	8.985994

3.035108	-0.79864	-4.30081	-1.37161	10.73455	294	2038.73	7.620082	3.962507	9.87801
3.339835	-0.80018	-4.24028	-1.50621	10.74682	293	1984.89	7.593319	3.228956	11.00913
3.392439	-0.78411	-4.7513	-1.49402	10.75937	292	2037.4	7.61943	5.077196	11.36991
3.53313	-0.83496	-4.59476	-1.49196	10.82419	291	2135.5	7.666456	7.497577	12.10037
3.552406	-0.88624	-5.03598	-1.48265	10.90883	290	2302.38	7.741699	9.390167	12.21712
3.540574	-0.8898	-5.29758	-1.46473	10.96853	289	2401	7.78364	9.575477	12.57451
3.545753	-0.87158	-5.00312	-1.42724	10.97898	287	2423	7.792762	10.0172	15.34899
2.973266	-0.60105	-4.33636	-1.25646	10.24956	143	630.15	6.445958	3.858563	8.083128
2.963044	-0.56037	-4.42994	-1.25609	10.37365	142	637	6.456769	3.662385	8.32609
3.031917	-0.59185	-4.49367	-1.23396	10.46707	140	653.8	6.482801	4.298095	9.487537
3.093268	-0.65579	-4.621	-1.22996	10.53314	139	667.81	6.504004	4.166419	10.85587
3.281837	-0.64571	-4.50441	-1.1359	10.5487	137	651.96	6.479983	5.268347	10.46962
3.287116	-0.6344	-4.82015	-1.06734	10.60309	135	667.63	6.503734	4.134854	10.03127
3.329648	-0.70091	-4.7811	-1.07552	10.66639	133	702.98	6.555328	4.085931	9.46678
3.346389	-0.79772	-4.9083	-1.08845	10.71275	131	750.57	6.620833	5.723243	9.524505
3.298047	-0.54861	-5.05864	-1.09237	10.76962	129	780	6.659294	4.169209	10.36257
3.554836	-0.60471	-4.27797	-1.08842	10.83094	126	805	6.690842	11.42522	18.16404
2.98084	-0.62168	-4.17534	-1.26856	10.16942	86	801.87	6.686946	3.770006	9.212735
2.959676	-0.6358	-3.92213	-1.24512	10.28712	85	827.9	6.718893	2.741781	10.27227
2.900258	-0.63587	-3.68124	-1.25758	10.37561	83	845.2	6.739573	3.23861	9.573666
2.908766	-0.60183	-4.20224	-1.26525	10.41823	82	859.42	6.756258	3.280997	9.300241
3.181344	-0.6025	-4.31088	-1.06729	10.39693	80	868.97	6.767309	2.96108	8.479652
3.204903	-0.61483	-4.22712	-1.03455	10.43485	79	896.62	6.798632	1.358655	7.366355
3.331505	-0.63963	-4.54017	-0.97571	10.48813	77	928.57	6.833646	3.716034	7.617259
3.337342	-0.66494	-4.81109	-1.01047	10.55056	76	973.88	6.881288	3.657677	8.102668
3.38896	-0.43198	-4.94825	-0.99423	10.62512	74	996	6.903747	4.992696	9.978739
3.463772	-0.50964	-3.92929	-0.91702	10.65566	72	1014	6.921658	6.842078	9.090223
3.348222	-0.48917	-4.92862	-1.63143	11.3609	3717	1339.62	7.200141	5.337428	10.12465
3.384875	-0.42377	-5.06814	-1.6277	11.40326	3785	1353.4	7.210375	7.02878	13.56685
3.439808	-0.41009	-4.81777	-1.63391	11.46945	3862	1410.6	7.25177	8.226184	16.43663
3.434089	-0.33478	-4.6159	-1.6356	11.5408	3892	1369.03	7.221858	9.257085	17.57209
3.634284	-0.36279	-4.80441	-1.46846	11.60081	3878	1405.55	7.248184	11.66561	18.95862
3.650192	-0.40294	-4.98071	-1.46316	11.70659	3892	1486.02	7.303857	11.73767	17.94109
3.685666	-0.40013	-5.32432	-1.473	11.80177	3890	1526.77	7.33091	13.28633	18.30444
3.711239	-0.39739	-5.13598	-1.4614	11.88976	3904	1566.66	7.356701	15.79807	18.72687
3.725016	-0.40589	-5.32068	-1.53566	11.94015	3914	1569	7.358194	16.60069	18.64553
3.753067	-0.39697	-5.21379	-1.5705	11.96275	3925	1576	7.362645	18.21066	22.21465
3.084477	-0.50506	-4.44053	-2.06049	11.02621	784	4281.62	8.362086	2.059974	7.874832
3.108551	-0.50161	-4.40337	-2.03359	11.10545	793	4580.9	8.42965	3.009677	11.27601
3.109264	-0.47976	-4.21014	-2.02952	11.19503	800	4956.6	8.508475	2.6325	12.49217
3.122064	-0.46249	-4.29893	-2.03496	11.27354	809	5012.54	8.519698	3.693096	13.07302
3.364751	-0.43251	-4.31494	-1.99543	11.3606	812	5114.7	8.539874	4.478977	12.66119
3.397557	-0.43677	-4.61545	-2.04757	11.43667	819	5458.95	8.605012	4.746468	10.97252
3.472006	-0.42424	-4.78775	-2.09	11.53471	823	5807.89	8.666973	7.773775	11.42795
3.515166	-0.41116	-4.92302	-2.07889	11.61284	825	6128.27	8.720668	9.395016	12.4068
3.548078	-0.40906	-4.96631	-2.06005	11.66693	827	6264	8.742575	10.58603	12.71099
3.613547	-0.41299	-5.03036	-2.01675	11.70629	828	6374	8.759982	13.02201	16.8913
2.976959	-0.32493	-4.89374	-2.11504	10.96523	538	3116.91	8.044598	2.507668	7.294318
2.994718	-0.32141	-4.51731	-2.11158	11.02022	549	3210.6	8.074213	3.661136	10.14185
2.979756	-0.32463	-4.5605	-2.0659	11.08376	559	3453.1	8.147028	4.108578	11.19826
2.985876	-0.34578	-4.43559	-2.04861	11.1356	569	3506.39	8.162342	4.410373	11.90776
3.334008	-0.35167	-4.5946	-1.87893	11.20199	578	3553.9	8.175801	4.498029	12.18164
3.346912	-0.36048	-4.2854	-1.91331	11.26937	587	3873.19	8.261834	4.181616	10.41939
3.437483	-0.35818	-4.75105	-1.94003	11.35758	596	4192.63	8.341084	6.832981	11.11447

3.509251	-0.35576	-4.79468	-1.9053	11.44282	606	4532.82	8.4191	9.752431	12.39657
3.536365	-0.34111	-4.98016	-1.82668	11.50055	616	4706	8.456594	11.06018	13.62793
3.569656	-0.35973	-4.68042	-1.85885	11.52028	625	4830	8.482602	12.95375	16.39416
3.053589	-0.53992	-4.10887	-1.59541	10.21475	428	1221.19	7.107581	2.703216	8.545966
3.099718	-0.48382	-4.01723	-1.53268	10.33192	428	1361.1	7.216048	3.582875	11.21163
3.185718	-0.48582	-3.70573	-1.55441	10.44593	429	1528.1	7.33178	4.843879	11.67594
3.173208	-0.44016	-3.96139	-1.5745	10.53423	430	1585.18	7.368454	4.971332	11.9172
3.358094	-0.40705	-3.99266	-1.51486	10.58891	431	1639.79	7.402324	5.347335	11.95927
3.467907	-0.37882	-3.96662	-1.56095	10.68478	432	1794.98	7.492749	7.234854	13.71151
3.542767	-0.38653	-4.07354	-1.56518	10.80145	434	1921.48	7.560851	8.714305	13.06124
3.602172	-0.39459	-4.45105	-1.64384	10.93423	435	2135.07	7.666255	11.2709	15.09304
3.690369	-0.48965	-4.29871	-1.60631	11.01141	436	2301	7.741099	14.41293	17.11035
3.696364	-0.54399	-4.34848	-1.62818	11.0415	437	2428	7.794823	15.89292	19.45722
3.0261	-0.58211	-4.50326	-2.09816	10.77746	308	1515.86	7.323738	3.965375	12.75258
3.059441	-0.571	-4.50805	-2.04683	10.87727	313	1579.5	7.364863	3.618018	14.06077
3.165636	-0.58112	-4.37635	-1.9924	10.97259	316	1700.7	7.438795	5.166642	16.57195
3.159964	-0.59342	-4.85955	-2.02061	11.06208	321	1855.79	7.526066	4.993221	16.76216
3.429084	-0.61607	-4.76011	-1.90244	11.12209	324	1851.86	7.523946	6.658013	17.5284
3.464279	-0.61311	-5.05093	-1.93521	11.21214	327	1968.58	7.585068	5.564775	13.05443
3.491681	-0.58587	-5.01604	-1.97753	11.33569	331	2112.72	7.655732	6.52113	13.19284
3.513924	-0.56001	-4.80642	-2.08012	11.45867	334	2313.82	7.746655	9.122797	13.61545
3.595707	-0.59686	-4.89004	-2.12051	11.53978	337	2402	7.784057	11.59219	15.72966
3.73556	-0.65355	-4.37309	-2.12379	11.56273	339	2483	7.817223	15.2977	19.11568
3.024583	-0.96231	-3.8718	-1.51963	10.16308	268	835.1	6.727551	4.417706	8.152619
2.989342	-0.9958	-3.70174	-1.44505	10.262	268	867.7	6.765846	3.285084	10.16965
3.302144	-0.89086	-4.08906	-1.41603	10.37199	268	947.1	6.853405	4.931929	13.96762
3.325514	-0.8726	-4.21608	-1.39508	10.46276	268	1018.52	6.926106	6.770525	13.50727
3.401749	-0.758	-4.26629	-1.33579	10.53039	268	1087.26	6.991416	4.260282	13.4545
3.529546	-0.68915	-4.07233	-1.3819	10.62011	269	1182.5	7.075386	5.133865	12.62604
3.623786	-0.67171	-4.15981	-1.37473	10.7117	270	1293.98	7.165478	5.894446	13.72149
3.66739	-0.65684	-4.14683	-1.38834	10.82669	270	1428.77	7.264569	11.24178	14.70058
3.760773	-0.51486	-4.01318	-1.35125	10.90852	270	1536	7.336937	14.06964	16.68235
3.788408	-0.54586	-4.44701	-1.35264	10.95195	270	1627	7.394493	13.82997	19.9849
3.110505	-0.55419	-4.15282	-2.05537	10.61106	611	3635.26	8.198436	3.557678	7.507765
3.121925	-0.51711	-4.06253	-1.98449	10.69982	614	3794.6	8.241334	3.3491	10.93795
3.181957	-0.49962	-4.02232	-1.95701	10.79288	616	4083.1	8.314611	4.590765	13.11111
3.17898	-0.50426	-4.12123	-1.95648	10.85771	620	4223.49	8.348417	5.794807	12.79543
3.457426	-0.50081	-4.37901	-1.90236	10.93676	624	5117.05	8.540334	6.647517	13.08844
3.499886	-0.46605	-4.32094	-1.90386	10.98934	630	5390.75	8.59244	7.602741	11.86385
3.568077	-0.54353	-4.19588	-1.9178	11.05078	634	5430.16	8.599724	10.12064	12.40211
3.62774	-0.63519	-4.27747	-1.88681	11.1017	637	6083.87	8.713396	14.55027	13.4932
3.686821	-0.70011	-4.61408	-1.88223	11.15484	639	6219	8.735364	17.30871	15.30716
3.736015	-0.801	-4.61898	-1.869	11.18199	642	6940	8.845057	19.90097	19.83592
3.058778	-0.70104	-5.08245	-1.82364	10.23642	567	2659.14	7.885758	3.352304	10.08377
3.033622	-0.73595	-4.92901	-1.75526	10.32538	572	2747.7	7.918519	3.790602	13.22864
3.043879	-0.71277	-4.69863	-1.73458	10.40771	574	2899.2	7.97219	4.664292	14.08334
3.04903	-0.69079	-4.76357	-1.7466	10.49077	578	2919.57	7.979192	5.788364	14.07037
3.360534	-0.63476	-4.83126	-1.69636	10.5542	582	2879.62	7.965414	9.17733	15.58962
3.418797	-0.59157	-4.7173	-1.68638	10.62925	586	2989.15	8.002745	9.466684	13.00302
3.549974	-0.6121	-4.24702	-1.69674	10.73036	589	3166.17	8.060278	15.94789	14.04122
3.646698	-0.63408	-7.43442	-1.68961	10.83396	591	3417.68	8.136717	20.0659	15.57824
3.733778	-0.66009	-8.77954	-1.6649	10.90331	594	3364	8.120886	23.71079	16.8082
3.774477	-0.71644	-9.10977	-1.65363	10.90945	596	3392	8.129175	25.00243	16.99441
3.071852	-0.94419	-4.3407	-1.82511	10.45504	310	1450.76	7.279843	4.680132	7.958461

2.999256	-0.85034	-4.37106	-1.79318	10.57513	311	1507.9	7.318473	4.432537	9.522799
2.964368	-0.74685	-4.60944	-1.75874	10.68819	312	1629.8	7.396213	3.509095	9.418819
3.034509	-0.7286	-4.49125	-1.74463	10.7921	313	1656.54	7.412487	3.577082	10.6447
3.326418	-0.7015	-4.81178	-1.59894	10.8594	315	1665.16	7.417676	5.313616	10.83308
3.355084	-0.63982	-4.27351	-1.64727	10.94807	317	1763.11	7.474834	6.614683	11.37711
3.373499	-0.60783	-4.45081	-1.70014	11.05357	318	1869	7.533159	8.825868	11.12084
3.460143	-0.57376	-4.37657	-1.75605	11.1718	319	2071.43	7.635994	12.14459	13.14588
3.471037	-0.50417	-4.36368	-1.74044	11.24781	319	2214	7.702556	12.10389	13.35525
3.577981	-0.54673	-4.38493	-1.62844	11.20758	309	2144	7.670429	15.21985	16.19851
3.258885	-0.82011	-5.00116	-1.68128	10.26695	311	1293.44	7.165061	3.654774	9.950605
3.158612	-0.71386	-4.71349	-1.63873	10.37982	311	1346.5	7.205264	3.609754	11.41608
3.19324	-0.72737	-4.61178	-1.6133	10.48296	312	1423.1	7.260593	3.313265	13.02815
3.074648	-0.65519	-4.7967	-1.64061	10.57595	312	1430.88	7.266045	2.939767	12.9438
3.23675	-0.63061	-3.9719	-1.60577	10.67255	312	1447.63	7.277683	2.857193	11.69587
3.296415	-0.6077	-5.03669	-1.5825	10.74949	313	1495.65	7.310316	2.363243	11.20676
3.392213	-0.56161	-5.03861	-1.59422	10.84017	313	1581.51	7.366136	4.164251	11.01747
3.48445	-0.51365	-4.60894	-1.58046	10.91077	313	1745.24	7.464647	6.488132	12.42965
3.532739	-0.54182	-4.57524	-1.60249	11.00383	313	1864	7.53048	8.459126	13.11007
3.622631	-0.58992	-4.56605	-1.59817	11.04351	314	1929	7.564757	10.92276	16.0089
3.362741	-0.56484	-5.07248	-2.06771	10.8213	617	4399.02	8.389137	5.409737	12.33306
3.392551	-0.52608	-5.38949	-2.04335	10.8649	634	4619.4	8.43802	5.974071	17.30336
3.381932	-0.50917	-5.17963	-2.00568	10.93362	647	4830.1	8.482622	6.798041	17.41815
3.388008	-0.49181	-5.41574	-2.00801	11.00058	659	5235.23	8.563166	7.401009	18.04288
3.581387	-0.46429	-5.54286	-1.7623	11.07467	670	5310.69	8.577477	7.205245	17.45424
3.64231	-0.43046	-5.40974	-1.81002	11.15154	683	5610.13	8.632329	8.682393	16.77757
3.716468	-0.39526	-5.52281	-1.80742	11.24135	697	5958.97	8.692653	12.90505	17.22226
3.771023	-0.36607	-6.48315	-1.8491	11.30989	709	6323.35	8.752005	16.01767	18.72781
3.793226	-0.37877	-6.60678	-1.83143	11.37316	717	6696	8.809265	17.76614	19.37888
3.819914	-0.38814	-6.25267	-1.84957	11.391	724	6926	8.843038	21.06412	23.36016
3.05492	-0.9661	-4.15539	-1.39789	10.00938	196	1112.21	7.014104	4.556201	7.844176
3.138325	-0.88347	-4.08322	-1.33145	10.09332	198	1153.9	7.050903	5.124749	10.31221
3.159075	-0.84928	-4.04577	-1.35574	10.18173	199	1237.7	7.12101	5.315316	12.42687
3.200317	-0.81049	-4.21661	-1.3622	10.26127	201	1307.99	7.176247	6.451817	13.29658
3.38059	-0.8083	-4.03695	-1.29194	10.33819	203	1334.32	7.196177	9.179646	14.75211
3.431641	-0.7913	-4.36851	-1.28881	10.41451	205	1359.65	7.214983	10.62311	12.9276
3.521071	-0.80386	-4.57095	-1.2877	10.50345	207	1444.95	7.27583	9.443344	13.2805
3.506367	-0.81729	-4.14494	-1.30722	10.59255	208	1703.04	7.44017	9.2189	14.10685
3.523899	-0.87534	-3.76448	-1.28914	10.66378	210	1907	7.553287	12.00351	14.05313
3.566725	-0.97647	-3.28045	-1.27919	10.69732	211	2029	7.615298	14.05041	16.33766
2.922	-0.59744	-4.4769	-1.15171	10.24046	287	185.28	5.221868	0.297146	6.006049
2.915862	-0.70193	-4.13361	-1.1183	10.34152	294	210.3	5.348535	4.258764	7.979372
2.902001	-0.67055	-4.76336	-1.12541	10.43567	297	232	5.446737	2.974617	7.448524
3.071857	-0.39355	-5.09657	-1.14301	10.52315	302	251.88	5.528953	2.73233	10.81788
3.447585	-0.72205	-5.1252	-1.10288	10.58921	305	272.36	5.607125	4.466372	17.15789
3.441621	-0.91512	-4.9052	-1.08906	10.66916	309	287.31	5.660562	2.929118	14.62322
3.490554	-0.90645	-4.42044	-1.13613	10.75002	314	304.95	5.720148	3.947752	14.58387
3.403557	-0.91662	-3.41865	-1.06592	10.82502	317	326.78	5.789287	6.91316	11.87871
3.401869	-0.91165	-3.19263	-1.05368	10.89542	321	355	5.872118	8.568246	10.82577
3.402744	-0.96154	-2.66863	-1.03671	10.92302	327	363	5.894403	7.97042	12.60711
3.117697	-0.63316	-3.66873	-1.37458	10.45921	357	717.03	6.575118	1.492945	6.8336
3.03721	-0.73953	-4.12779	-1.33666	10.57592	361	723.5	6.584101	2.456935	9.123281
2.985191	-0.67038	-4.3198	-1.44791	10.68114	366	813.3	6.7011	1.926081	8.648377
3.093444	-0.68495	-4.46406	-1.4874	10.78541	369	867.24	6.765316	3.015174	12.31429
3.341867	-0.63768	-4.7484	-1.44222	10.86819	373	875.37	6.774647	2.087452	10.12779

3.400827	-0.58139	-4.8282	-1.5049	10.97382	378	924.89	6.829675	2.593286	11.04519
3.457386	-0.56121	-4.50367	-1.53203	11.06931	382	996.55	6.904299	6.231504	11.67923
3.510827	-0.53866	-6.3547	-1.55904	11.134	384	1118.79	7.020003	10.37417	14.41267
3.538853	-0.55945	-4.62889	-1.58299	11.21636	387	1160	7.056175	12.05102	16.15347
3.547891	-0.54619	-5.90878	-1.63253	11.26823	390	1186	7.078341	12.68223	14.81212
3.045123	-0.76401	-5.01234	-1.50473	10.17107	164	1751.44	7.468194	4.500456	9.118111
3.040632	-0.70218	-4.8991	-1.47902	10.29644	164	1830.7	7.512454	5.67485	11.40273
3.115293	-0.71969	-4.73026	-1.44991	10.39666	165	1949	7.575072	6.039234	12.08249
3.10826	-0.89892	-4.60764	-1.44712	10.47906	166	2014.79	7.60827	6.632665	12.94843
3.317537	-0.79647	-4.94501	-1.39796	10.52272	167	1992.4	7.597095	6.053782	11.33372
3.386029	-0.80654	-4.73719	-1.42014	10.60403	168	2101.02	7.650178	7.353179	11.6957
3.50698	-0.78834	-4.81209	-1.47237	10.7328	169	2205.18	7.698565	12.99653	12.54217
3.512794	-0.76984	-5.49084	-1.48602	10.8524	169	2459.49	7.807709	13.78633	13.88445
3.54693	-0.77334	-5.45089	-1.49971	10.92628	170	2636	7.877018	14.55188	15.38462
3.586786	-0.78853	-5.25992	-1.46562	10.96835	170	2865	7.960324	17.60699	17.58955
3.118579	-1.39403	-4.46046	-0.91488	9.681843	200	944.13	6.850264	2.137333	8.463278
3.015079	-1.33569	-4.58349	-0.89472	9.849401	204	1046.7	6.953398	2.152105	10.94431
3.080114	-1.23239	-4.28608	-0.95028	10.00284	206	1126.3	7.026693	3.546236	10.9976
2.75737	-1.1932	-3.98588	-0.95136	10.13066	209	1173.74	7.067951	1.334898	8.523337
3.192664	-1.23506	-4.3394	-0.98422	10.25931	210	1174.21	7.068351	1.267489	9.420434
3.259143	-1.15682	-4.60114	-1.01763	10.36056	213	1241.78	7.124301	1.768224	9.791246
3.370887	-1.19057	-4.13971	-1.08169	10.49094	216	1384.89	7.233376	5.715052	10.58229
3.380401	-1.22769	-3.71843	-1.11597	10.60339	217	1482.12	7.301229	6.459719	10.7519
3.380405	-1.24636	-3.86054	-1.03637	10.68572	218	1541	7.340187	6.701444	11.16858
3.423125	-1.28931	-3.75966	-1.13805	10.74408	219	1586	7.36897	10.08766	14.13367
3.038157	-1.34264	-4.38067	-1.17886	9.935616	117	1204.07	7.093463	4.89046	10.87113
3.033906	-1.50018	-4.42864	-1.1334	10.08548	118	1315.9	7.182276	4.416901	11.01436
3.136844	-1.53817	-4.17548	-1.1413	10.22792	118	1459.8	7.286055	4.10296	13.24897
3.031969	-1.60549	-4.52591	-1.15183	10.31616	118	1529.38	7.332618	6.144481	12.45026
3.446131	-1.70088	-4.6658	-1.15509	10.37714	118	1438.61	7.271433	6.979233	14.70196
3.549146	-1.63075	-4.72091	-1.18219	10.46456	119	1410.52	7.251714	8.82076	15.47691
3.574474	-1.57936	-4.86473	-1.17428	10.58299	119	1538.1	7.338303	11.99219	15.43141
3.562004	-1.52749	-4.89996	-1.23463	10.70203	119	1679.08	7.426001	14.04908	14.54376
3.574405	-1.57561	-4.44104	-1.23266	10.80615	120	1812	7.502186	13.79529	14.90825
3.651652	-1.59835	-5.0461	-1.25875	10.8599	120	2025	7.613325	18.38263	17.37453
3.187673	-0.76673	-4.37475	-1.42411	10.38798	183	982.47	6.89007	7.731731	11.59586
3.142937	-0.75317	-4.36065	-1.44807	10.53084	184	1066.7	6.972325	6.912257	12.49308
2.963784	-0.7003	-4.27309	-1.46781	10.64318	185	1152.2	7.049428	4.657974	9.205785
3.135055	-0.69526	-4.11048	-1.47974	10.72788	186	1226.01	7.11152	5.855867	10.29518
3.329185	-0.66113	-4.31019	-1.40859	10.75051	187	1221.73	7.108023	3.46024	9.400716
3.409529	-0.73872	-4.09444	-1.46766	10.80651	188	1357.06	7.213076	4.676289	11.89636
3.452978	-0.75143	-4.22359	-1.49131	10.91901	190	1494.75	7.309714	8.087309	11.80862
3.53144	-0.76343	-4.83453	-1.50746	11.02051	191	1594.17	7.374108	10.84841	12.74714
3.574728	-0.73357	-4.58309	-1.5064	11.0899	192	1912	7.555905	13.41852	13.39171
3.659303	-0.83583	-4.61343	-1.47798	11.09539	192	1741	7.462215	17.83711	16.51335
3.035631	-1.86716	-4.39223	-0.98922	9.844056	56	923.45	6.828117	0.233683	4.892824
2.929821	-1.77034	-3.79379	-0.96263	9.958969	56	994.6	6.90234	0.491883	8.433907
2.93219	-1.63915	-3.53031	-0.95709	10.07099	56	1073.2	6.9784	2.687172	9.171907
2.928582	-1.58048	-3.81605	-0.94188	10.15518	56	1095.48	6.998948	3.73019	9.678815
3.312269	-1.70096	-3.9793	-0.79585	10.16377	55	1098.72	7.001901	2.418581	9.271163
3.322312	-1.53388	-4.07313	-0.78525	10.21815	55	1065.15	6.970871	2.71862	9.120488
3.315247	-1.5395	-4.40864	-0.79762	10.2786	55	1164.37	7.059936	4.376113	9.550421
3.428639	-1.54395	-2.3685	-0.7647	10.37904	55	1289.52	7.162025	7.440765	11.61655
3.444044	-1.41785	-4.25036	-0.7913	10.4547	55	1288	7.160846	10.45027	11.98938

3.448632	-1.43824	-4.17547	-0.77068	10.48704	55	1376	7.226936	11.91029	12.44984
2.969541	-2.18528	-3.9571	-0.34817	10.09547	8	560.68	6.32915	1.73725	8.688843
3.012031	-1.98461	-4.14983	-0.27669	10.19761	8	602.2	6.400589	3.036678	10.95998
2.992316	-2.03454	-3.8434	-0.33299	10.30912	8	676.3	6.516637	2.513815	10.63434
2.953032	-2.07558	-4.1205	-0.31574	10.38028	8	723.21	6.5837	2.502858	9.631316
3.217227	-2.34314	-4.0539	-0.28311	10.45975	8	658	6.489205	2.149845	9.689235
3.33788	-2.36495	-3.69163	-0.3927	10.5705	8	637.51	6.45757	0.603836	10.55884
3.372607	-2.13852	-4.09398	-0.47668	10.65044	8	687.01	6.532349	4.558893	10.31725
3.471955	-1.96201	-4.85301	-0.51166	10.75479	8	738.34	6.604404	7.306702	12.71518
3.58664	-1.95405	-4.64793	-0.45624	10.8193	8	716	6.57368	8.956721	16.02189
3.610685	-1.70419	-5.37057	-0.44266	10.83654	9	742	6.609349	11.92705	16.09182
3.012271	-1.2286	-3.51616	-1.00672	10.31431	98	724.54	6.585537	4.132499	5.440752
2.981173	-1.12106	-3.64437	-0.90236	10.39234	99	741.8	6.60908	2.731279	8.335575
2.979895	-1.08936	-3.47043	-0.92557	10.46695	100	811.2	6.698514	5.027048	6.517655
2.982199	-1.0193	-3.44918	-0.90535	10.51366	102	848.75	6.743765	5.409152	8.244687
3.259345	-0.93908	-3.39055	-0.81785	10.54207	103	878.33	6.778022	2.569541	8.262379
3.283757	-1.15774	-3.31361	-0.79619	10.60507	105	886.91	6.787744	4.083074	11.20563
3.331128	-1.24057	-3.63543	-0.84641	10.73025	106	978.3	6.885817	6.547175	9.642174
3.378046	-1.31089	-3.9992	-0.90568	10.81203	107	1064.85	6.970589	9.766212	9.483644
3.803694	-1.29438	-3.89548	-0.9579	10.86927	108	1084	6.988413	17.23705	18.53761
3.871872	-1.31736	-3.979	-0.98124	10.91547	109	1038	6.945051	15.58985	20.74068
3.121687	-0.86755	-3.89638	-1.05057	10.29634	14	839.1	6.73233	4.916193	10.59212
3.117888	-0.86349	-3.36917	-1.00242	10.40738	14	1151.5	7.04882	5.382411	11.51889
3.127877	-0.93404	-3.27086	-1.00661	10.51835	14	1539.8	7.339408	5.251584	11.56678
3.129083	-0.99172	-3.16166	-1.02689	10.60145	14	1900.24	7.549736	4.069467	11.41442
3.370903	-1.11458	-3.47312	-0.89447	10.58456	15	2160.34	7.678021	4.783334	12.75835
3.417208	-1.11541	-3.42716	-0.84469	10.59713	15	2316.46	7.747796	5.754195	12.24741
3.455853	-1.20679	-3.36554	-0.87821	10.72494	15	2542.85	7.841041	6.501554	12.25618
3.475849	-1.30738	-4.44641	-0.93825	10.84424	15	2686.48	7.895987	8.827453	12.37393
3.532336	-0.22324	-4.28499	-0.93923	10.88822	16	2868	7.96137	12.06212	13.54871
3.60327	-1.20381	-4.49552	-0.92842	10.88942	16	3099	8.038835	14.66047	17.43899

G	lnE	lnS	lnG	east	middle	west	_est_m0	lnESG_Gc	lnE_Gcre
73.13416	1.904565	2.669028	4.292295	1	0	0	1	-2.54415	-1.41043
76.15558	1.867505	2.815118	4.332778	1	0	0	1	-2.65188	-1.42172
75.20895	1.963079	2.842179	4.32027	1	0	0	1	-2.5873	-1.45789
77.08038	2.217129	2.925565	4.344849	1	0	0	1	-2.10493	-1.31889
91.44076	2.236031	2.933723	4.515691	1	0	0	1	-1.15625	-0.70395
93.06931	2.422955	2.950077	4.533344	1	0	0	1	-1.54843	-1.00937
99.79939	2.801062	3.0221	4.603162	1	0	0	1	-1.29514	-0.94957
102.7314	2.970995	3.084942	4.632118	1	0	0	1	-1.09945	-0.84331
101.5833	3.014638	3.086617	4.62088	1	0	0	1	-0.82381	-0.64145
103.5424	3.060889	3.211936	4.639981	1	0	0	1	-1.29664	-1.02368
63.08398	1.333432	2.385278	4.144467	1	0	0	1	-2.88146	-1.17986
68.05767	1.495451	2.472746	4.220356	1	0	0	1	-2.93421	-1.31439
60.7436	1.154187	2.522123	4.106662	1	0	0	1	-2.53649	-0.90395
60.20971	1.514364	2.669652	4.097834	1	0	0	1	-2.50237	-1.16103
75.01749	1.381137	2.68323	4.317721	1	0	0	1	-2.52966	-1.01725
84.71266	1.705543	2.812907	4.439265	1	0	0	1	-0.89049	-0.42486
87.14818	2.335263	2.841121	4.46761	1	0	0	1	-1.34305	-0.86056
90.43262	2.530198	2.817145	4.504605	1	0	0	1	-3.00822	-2.06377
92.56318	2.761916	2.909735	4.527892	1	0	0	1	-2.00777	-1.4807
92.44734	2.919944	2.971775	4.526639	1	0	0	1	-2.5223	-1.95449
52.39474	0.653784	2.061035	3.958806	1	0	0	1	-2.47376	-0.53581
49.02107	0.872596	2.357356	3.89225	1	0	0	1	-2.64365	-0.76513
48.35748	0.960032	2.352432	3.878621	1	0	0	1	-2.65215	-0.84616
49.05428	1.188888	2.370009	3.892927	1	0	0	1	-2.46309	-0.96442
65.13116	1.301875	2.402052	4.176403	1	0	0	1	-2.60126	-1.0332
71.29102	1.792951	2.447966	4.26677	1	0	0	1	-2.6933	-1.42438
74.17356	2.193152	2.523963	4.306408	1	0	0	1	-2.66761	-1.68925
75.41514	2.6639	2.548921	4.323008	1	0	0	1	-2.64747	-1.996
75.91544	2.855178	2.629164	4.32962	1	0	0	1	-2.76076	-2.20359
84.57685	3.005196	2.886282	4.437661	1	0	0	1	-3.48591	-2.86692
58.21694	1.863822	2.291143	4.064177	0	1	0	1	-2.37466	-1.37685
53.9459	2.085249	2.404078	3.987982	0	1	0	1	-2.14747	-1.40192
55.39986	2.0191	2.459557	4.014577	0	1	0	1	-1.77624	-1.11536
54.36493	2.091523	2.385072	3.995719	0	1	0	1	-1.64761	-1.07763
77.06222	2.058894	2.420989	4.344613	0	1	0	1	-1.6483	-0.98027
75.31673	2.028236	2.423	4.321702	0	1	0	1	-1.43289	-0.84275
76.92313	2.04425	2.400559	4.342807	0	1	0	1	-1.45722	-0.85994
76.0451	2.118939	2.407575	4.331326	0	1	0	1	-1.47153	-0.90067
75.44842	2.441434	2.426393	4.323449	0	1	0	1	-1.67337	-1.17041
80.00584	2.611385	2.534414	4.3821	0	1	0	1	-1.29769	-0.96237
43.57867	0.201683	1.799879	3.774568	0	1	0	1	-3.09962	-0.22072
43.22155	0.274094	2.105566	3.766339	0	1	0	1	-2.95178	-0.28203
41.4795	0.246301	2.112698	3.725199	0	1	0	1	-2.97505	-0.25842
43.64833	0.426613	2.233474	3.776165	0	1	0	1	-3.42973	-0.50428
73.08672	0.97125	2.344963	4.291646	0	1	0	1	-4.00324	-1.15736
75.36721	1.132298	2.457258	4.322372	0	1	0	1	-4.02758	-1.33947
79.27086	1.79568	2.500949	4.37287	0	1	0	1	-3.98127	-2.05262
80.51195	2.394548	2.451861	4.388406	0	1	0	1	-3.92561	-2.65642
80.52913	2.48565	2.422463	4.388619	0	1	0	1	-4.00499	-2.80768
86.66289	2.507221	2.550942	4.462026	0	1	0	1	-4.14791	-2.89655
51.94195	0.997944	1.873717	3.950127	1	0	0	1	-2.44735	-0.80954
51.9253	1.084375	2.092584	3.949806	1	0	0	1	-2.47987	-0.88279
49.09288	0.922316	2.195667	3.893714	1	0	0	1	-2.29779	-0.70732

49.16922	1.376877	2.290311	3.895268	1	0	0	1	-2.42397	-1.09964
70.80875	1.172159	2.398725	4.259983	1	0	0	1	-2.67248	-0.93794
72.60902	1.624759	2.43097	4.285089	1	0	0	1	-2.66003	-1.27398
82.91309	2.01458	2.493236	4.417793	1	0	0	1	-2.95004	-1.6821
82.90419	2.239663	2.502839	4.417686	1	0	0	1	-3.14829	-1.98488
81.13531	2.259205	2.531672	4.396118	1	0	0	1	-3.15039	-2.01023
85.04064	2.304304	2.73105	4.443129	1	0	0	1	-3.09042	-2.00839
49.74641	1.350295	2.089779	3.906938	0	1	0	1	-1.78708	-0.81159
48.90664	1.298115	2.119394	3.889913	0	1	0	1	-1.66041	-0.72743
50.66369	1.458172	2.249979	3.92521	0	1	0	1	-1.79443	-0.86301
53.59483	1.427057	2.384706	3.981453	0	1	0	1	-2.02852	-0.93584
66.52541	1.661717	2.348478	4.197584	0	1	0	1	-2.11913	-1.07299
65.98511	1.419452	2.305707	4.189429	0	1	0	1	-2.08534	-0.9005
70.07609	1.40755	2.247789	4.249582	0	1	0	1	-2.33377	-0.98656
69.79755	1.744536	2.253868	4.245599	0	1	0	1	-2.66949	-1.39166
66.50127	1.427726	2.338201	4.197221	0	1	0	1	-1.80933	-0.78326
88.26879	2.435823	2.899444	4.480387	0	1	0	1	-2.14964	-1.47296
48.32972	1.327077	2.220587	3.878047	0	1	0	1	-1.85314	-0.82502
47.68811	1.008608	2.329448	3.864682	0	1	0	1	-1.88176	-0.64127
44.50134	1.175144	2.259016	3.795519	0	1	0	1	-1.84419	-0.74724
44.99736	1.188147	2.23004	3.806604	0	1	0	1	-1.75058	-0.71506
62.35811	1.085554	2.13767	4.132894	0	1	0	1	-1.91675	-0.65404
65.08438	0.306496	1.996923	4.175684	0	1	0	1	-1.97048	-0.18844
72.44184	1.312657	2.030416	4.282784	0	1	0	1	-2.13095	-0.83962
72.50682	1.296828	2.092193	4.28368	0	1	0	1	-2.21913	-0.86231
73.76955	1.607976	2.300457	4.300946	0	1	0	1	-1.46395	-0.69461
70.18488	1.923091	2.2072	4.251133	0	1	0	1	-1.76526	-0.98007
71.08125	1.674744	2.314973	4.263824	1	0	0	1	-1.63786	-0.81924
69.39618	1.950013	2.607629	4.239832	1	0	0	1	-1.43439	-0.82635
70.35055	2.107322	2.799512	4.25349	1	0	0	1	-1.41064	-0.8642
67.70274	2.225389	2.866312	4.215127	1	0	0	1	-1.14967	-0.74502
84.23576	2.456645	2.942259	4.433619	1	0	0	1	-1.31849	-0.89125
85.59224	2.462803	2.887093	4.449594	1	0	0	1	-1.47082	-0.99237
87.84529	2.586736	2.907144	4.475577	1	0	0	1	-1.47473	-1.03502
88.01181	2.759887	2.929959	4.477471	1	0	0	1	-1.4748	-1.09674
88.99089	2.809444	2.925606	4.488534	1	0	0	1	-1.51196	-1.14034
91.55006	2.902007	3.100752	4.516886	1	0	0	1	-1.48985	-1.152
57.39951	0.722694	2.063672	4.050036	1	0	0	1	-1.55786	-0.36501
54.68616	1.101833	2.422677	4.001611	1	0	0	1	-1.55929	-0.55269
55.06371	0.967934	2.525102	4.008491	1	0	0	1	-1.4917	-0.46438
54.26656	1.306465	2.570551	3.993908	1	0	0	1	-1.44392	-0.60423
71.74561	1.499395	2.538542	4.273127	1	0	0	1	-1.45529	-0.64851
73.79107	1.557401	2.395394	4.301238	1	0	0	1	-1.48394	-0.68022
77.23366	2.050756	2.436062	4.346835	1	0	0	1	-1.47297	-0.87002
78.89314	2.240179	2.518245	4.368094	1	0	0	1	-1.44528	-0.92106
80.76955	2.359535	2.542467	4.3916	1	0	0	1	-1.45138	-0.9652
84.64816	2.566641	2.826799	4.438503	1	0	0	1	-1.49234	-1.05999
50.51218	0.919353	1.987096	3.922215	1	0	0	1	-0.9673	-0.29872
47.51858	1.297774	2.31667	3.861121	1	0	0	1	-0.96254	-0.41712
45.20974	1.413077	2.415758	3.811312	1	0	0	1	-0.96732	-0.45873
44.95863	1.483959	2.47719	3.805743	1	0	0	1	-1.03246	-0.51312
68.62998	1.503639	2.49993	4.22873	1	0	0	1	-1.17246	-0.52878
70.48746	1.430698	2.343668	4.255435	1	0	0	1	-1.2065	-0.51574
75.21362	1.921761	2.408248	4.320332	1	0	0	1	-1.23124	-0.68834

77.95374	2.277517	2.517419	4.356115	1	0	0	1	-1.24847	-0.81026
78.17304	2.403351	2.612121	4.358925	1	0	0	1	-1.20629	-0.81981
83.4306	2.561386	2.796925	4.424015	1	0	0	1	-1.28412	-0.92141
53.97885	0.994442	2.145459	3.988592	0	1	0	1	-1.64868	-0.53692
53.22979	1.276166	2.416952	3.974618	0	1	0	1	-1.49971	-0.61744
57.46926	1.577716	2.45753	4.05125	0	1	0	1	-1.54768	-0.76648
56.19819	1.603688	2.477982	4.028884	0	1	0	1	-1.39673	-0.70588
69.78609	1.676598	2.481507	4.245435	0	1	0	1	-1.36691	-0.68246
75.78548	1.97891	2.618236	4.327907	0	1	0	1	-1.31372	-0.74966
81.73554	2.164966	2.569649	4.403489	0	1	0	1	-1.3694	-0.83683
83.49444	2.422224	2.714234	4.42478	0	1	0	1	-1.42138	-0.95579
88.47394	2.668126	2.839684	4.482708	0	1	0	1	-1.80701	-1.30646
90.49438	2.765874	2.968218	4.505288	0	1	0	1	-2.01079	-1.50461
49.01007	1.3776	2.545734	3.892026	1	0	0	1	-1.76154	-0.80192
50.38921	1.285926	2.643389	3.919777	1	0	0	1	-1.74695	-0.73427
53.89384	1.642223	2.807712	3.987016	1	0	0	1	-1.83962	-0.95433
53.44275	1.608081	2.819124	3.978611	1	0	0	1	-1.87518	-0.95426
72.74701	1.895821	2.863822	4.286988	1	0	0	1	-2.11255	-1.16796
77.07401	1.716457	2.569127	4.344766	1	0	0	1	-2.12399	-1.05238
78.6394	1.875048	2.579674	4.364873	1	0	0	1	-2.04565	-1.09853
77.83601	2.210776	2.611205	4.354604	1	0	0	1	-1.96782	-1.23805
81.83295	2.450332	2.755548	4.40468	1	0	0	1	-2.14613	-1.4625
92.57454	2.727703	2.950509	4.528014	1	0	0	1	-2.44136	-1.78268
51.12358	1.485621	2.098339	3.934246	0	1	0	1	-2.9106	-1.42963
47.79963	1.189392	2.319408	3.867018	0	1	0	1	-2.9768	-1.1844
64.7923	1.59573	2.636742	4.171187	0	1	0	1	-2.94173	-1.42157
65.02535	1.912579	2.603228	4.174777	0	1	0	1	-2.90185	-1.66892
74.14084	1.449335	2.599314	4.305966	0	1	0	1	-2.57854	-1.0986
84.3791	1.635859	2.535761	4.43532	0	1	0	1	-2.43237	-1.12734
92.61693	1.774011	2.618963	4.528472	0	1	0	1	-2.43415	-1.19163
91.31099	2.419637	2.687887	4.514271	0	1	0	1	-2.40887	-1.5893
97.98634	2.644019	2.814351	4.584828	0	1	0	1	-1.93629	-1.36131
100.7716	2.626838	2.994977	4.612856	0	1	0	1	-2.06793	-1.43388
58.23312	1.269108	2.015938	4.064454	1	0	0	1	-1.72382	-0.70333
55.63934	1.208692	2.392239	4.01889	1	0	0	1	-1.61437	-0.62502
56.54995	1.524047	2.57346	4.035124	1	0	0	1	-1.58978	-0.76145
55.38634	1.756962	2.549088	4.014333	1	0	0	1	-1.60303	-0.88597
76.80408	1.894243	2.571729	4.341258	1	0	0	1	-1.73152	-0.94866
79.69437	2.028509	2.473496	4.378199	1	0	0	1	-1.63111	-0.94538
83.64139	2.314576	2.517867	4.426538	1	0	0	1	-1.93935	-1.25803
84.66144	2.677609	2.602186	4.43866	1	0	0	1	-2.30431	-1.7008
86.9588	2.85121	2.72832	4.465435	1	0	0	1	-2.58117	-1.99616
92.62166	2.990768	2.987494	4.528523	1	0	0	1	-2.99254	-2.3956
55.32067	1.209648	2.310927	4.013146	0	1	0	1	-2.14432	-0.84801
48.8663	1.332525	2.582384	3.889088	0	1	0	1	-2.23259	-0.98067
47.92512	1.539936	2.644992	3.86964	0	1	0	1	-2.16958	-1.09762
47.29851	1.75585	2.644071	3.856479	0	1	0	1	-2.10625	-1.21293
65.32114	2.216736	2.746605	4.179316	0	1	0	1	-2.13312	-1.40709
68.98468	2.247779	2.565182	4.233884	0	1	0	1	-2.02245	-1.32971
74.29819	2.769326	2.641997	4.308086	0	1	0	1	-2.17295	-1.69512
79.24287	2.999022	2.745875	4.372518	0	1	0	1	-2.31229	-1.90161
84.82605	3.16593	2.821867	4.440603	0	1	0	1	-2.46462	-2.0898
86.02288	3.218973	2.832884	4.454613	0	1	0	1	-2.70418	-2.30619
51.99351	1.543326	2.074236	3.951119	0	1	0	1	-2.90042	-1.4572

46.16026	1.488972	2.253689	3.832119	0	1	0	1	-2.55038	-1.26613
45.12493	1.255358	2.24271	3.809435	0	1	0	1	-2.21395	-0.93757
48.0508	1.274547	2.365062	3.872259	0	1	0	1	-2.21094	-0.92863
67.22239	1.670273	2.382605	4.208006	0	1	0	1	-2.33347	-1.17169
67.80654	1.889292	2.431603	4.216659	0	1	0	1	-2.14664	-1.2088
67.4511	2.177687	2.40882	4.211403	0	1	0	1	-2.05051	-1.32366
70.0302	2.496884	2.576108	4.248927	0	1	0	1	-1.98531	-1.43262
70.90536	2.493527	2.591909	4.261346	0	1	0	1	-1.75001	-1.25717
79.7625	2.722601	2.784919	4.379054	0	1	0	1	-1.9562	-1.48854
65.3849	1.296034	2.297633	4.180291	0	1	0	1	-2.67263	-1.06289
57.55712	1.28364	2.435023	4.052778	0	1	0	1	-2.25479	-0.91633
60.69571	1.197934	2.567113	4.105873	0	1	0	1	-2.32266	-0.87134
52.16762	1.07833	2.560617	3.954462	0	1	0	1	-2.01447	-0.70651
63.00195	1.04984	2.459236	4.143166	0	1	0	1	-2.04113	-0.66204
67.32858	0.860035	2.416517	4.209585	0	1	0	1	-2.00324	-0.52265
73.84978	1.426536	2.399482	4.302033	0	1	0	1	-1.90512	-0.80116
78.72417	1.869975	2.520085	4.36595	0	1	0	1	-1.78979	-0.96051
80.9093	2.135246	2.57338	4.393329	0	1	0	1	-1.91411	-1.15692
88.73096	2.390849	2.773145	4.485609	0	1	0	1	-2.13707	-1.41042
71.79646	1.6882	2.512284	4.273835	1	0	0	1	-1.89941	-0.95356
69.38967	1.787428	2.8509	4.239738	1	0	0	1	-1.78477	-0.94034
67.48701	1.916635	2.857513	4.211935	1	0	0	1	-1.72197	-0.97589
66.74048	2.001616	2.892751	4.200812	1	0	0	1	-1.66627	-0.98442
85.473	1.974809	2.859583	4.448201	1	0	0	1	-1.66279	-0.91688
88.89195	2.161297	2.820043	4.487422	1	0	0	1	-1.56788	-0.93036
93.03522	2.557619	2.846203	4.532978	1	0	0	1	-1.46896	-1.01091
95.33307	2.773692	2.930009	4.557377	1	0	0	1	-1.38046	-1.01537
95.85957	2.877295	2.964184	4.562884	1	0	0	1	-1.43676	-1.08983
99.48092	3.047571	3.151032	4.599966	1	0	0	1	-1.48266	-1.18288
52.79859	1.516489	2.059771	3.966485	1	0	0	1	-2.95137	-1.46508
54.87537	1.634081	2.333328	4.005064	1	0	0	1	-2.77261	-1.44366
54.42401	1.670593	2.519861	3.996805	1	0	0	1	-2.68293	-1.4188
55.4572	1.864362	2.587507	4.015612	1	0	0	1	-2.59383	-1.51105
65.66148	2.216989	2.691386	4.184513	1	0	0	1	-2.73252	-1.79199
69.08827	2.363032	2.559365	4.235385	1	0	0	1	-2.71547	-1.86987
78.57074	2.24531	2.586297	4.363999	1	0	0	1	-2.83045	-1.80492
76.49438	2.221256	2.646661	4.337217	1	0	0	1	-2.8657	-1.8154
75.53635	2.485199	2.642845	4.324614	1	0	0	1	-3.0846	-2.17538
81.40886	2.642652	2.793473	4.399484	1	0	0	1	-3.48281	-2.58048
49.3185	-1.21353	1.792767	3.898299	1	0	0	1	-1.74573	0.725018
43.06481	1.448979	2.07686	3.762706	1	0	0	1	-2.04674	-1.01709
44.11274	1.090115	2.008016	3.786749	1	0	0	1	-1.94594	-0.73098
51.08791	1.005155	2.381201	3.933548	1	0	0	1	-1.20894	-0.39558
74.71586	1.496576	2.842458	4.313693	1	0	0	1	-2.48931	-1.0806
75.99652	1.074701	2.682611	4.330688	1	0	0	1	-3.14951	-0.98348
79.70831	1.373146	2.679916	4.378374	1	0	0	1	-3.16401	-1.24469
71.26744	1.933427	2.474748	4.266439	1	0	0	1	-3.11977	-1.77222
70.51452	2.148063	2.381929	4.255819	1	0	0	1	-3.10131	-1.95828
77.74661	2.075737	2.534261	4.353455	1	0	0	1	-3.27189	-1.99591
59.32003	0.400751	1.921852	4.082947	0	0	1	1	-1.97401	-0.25374
50.85052	0.898915	2.210829	3.92889	0	0	1	1	-2.24611	-0.66478
48.6903	0.655487	2.157372	3.88548	0	0	1	1	-2.00121	-0.43943
52.6119	1.103658	2.510761	3.962942	0	0	1	1	-2.11886	-0.75595
72.43716	0.735944	2.315283	4.28272	0	0	1	1	-2.13103	-0.46929

76.15768	0.952926	2.401995	4.332806	0	0	1	1	-1.9772	-0.55402
77.79951	1.829618	2.457812	4.354135	0	0	1	1	-1.94032	-1.0268
76.16894	2.339319	2.668107	4.332954	0	0	1	1	-1.89114	-1.2601
75.55347	2.489149	2.782135	4.324841	0	0	1	1	-1.97981	-1.39255
71.12273	2.540202	2.695446	4.264407	0	0	1	1	-1.93782	-1.38743
51.44444	1.504179	2.210263	3.940502	0	0	1	1	-2.3265	-1.1492
47.74141	1.736044	2.433853	3.865799	0	0	1	1	-2.13507	-1.21902
51.76981	1.798277	2.491757	3.946807	0	0	1	1	-2.24206	-1.29421
49.93801	1.892007	2.560974	3.910782	0	0	1	1	-2.79409	-1.70077
66.7108	1.800683	2.427782	4.200367	0	0	1	1	-2.64232	-1.43419
69.44759	1.995133	2.459221	4.240572	0	0	1	1	-2.73096	-1.60915
74.34848	2.564683	2.529097	4.308763	0	0	1	1	-2.76471	-2.02185
72.80656	2.623677	2.630769	4.287806	0	0	1	1	-2.7043	-2.01982
74.03515	2.67772	2.733368	4.30454	0	0	1	1	-2.74299	-2.07079
73.46777	2.868296	2.867305	4.296847	0	0	1	1	-2.8283	-2.26175
57.11456	0.759559	2.135736	4.045059	0	0	1	1	-4.34739	-1.05885
47.97557	0.766446	2.39282	3.870692	0	0	1	1	-4.02721	-1.02373
50.6332	1.265887	2.397677	3.924608	0	0	1	1	-3.7959	-1.56007
37.3386	0.288855	2.142808	3.620028	0	0	1	1	-3.29011	-0.34466
62.23238	0.237038	2.242881	4.130876	0	0	1	1	-3.94314	-0.29276
66.37349	0.569976	2.281489	4.195298	0	0	1	1	-3.77024	-0.65936
70.86017	1.743103	2.359182	4.260708	0	0	1	1	-4.01328	-2.07529
70.78159	1.865586	2.375082	4.259599	0	0	1	1	-4.1501	-2.29037
70.12612	1.902323	2.413104	4.250295	0	0	1	1	-4.2132	-2.37098
74.31883	2.311313	2.64856	4.308364	0	0	1	1	-4.41345	-2.97999
47.63288	1.587286	2.38611	3.863523	0	0	1	1	-4.07915	-2.13116
47.48759	1.485438	2.399199	3.860469	0	0	1	1	-4.55142	-2.22843
52.46021	1.411709	2.58392	3.960055	0	0	1	1	-4.825	-2.17145
44.40788	1.815554	2.521741	3.793417	0	0	1	1	-4.86781	-2.91486
73.14103	1.942939	2.687981	4.292389	0	0	1	1	-5.86144	-3.3047
79.8858	2.177108	2.739349	4.380598	0	0	1	1	-5.78778	-3.55033
79.44019	2.484256	2.736405	4.375004	0	0	1	1	-5.64537	-3.92353
76.95107	2.642557	2.677162	4.34317	0	0	1	1	-5.44093	-4.03649
78.15676	2.624327	2.701915	4.358716	0	0	1	1	-5.63186	-4.13491
78.65904	2.911406	2.855005	4.365123	0	0	1	1	-5.83661	-4.65344
56.44687	2.045333	2.450648	4.0333	0	0	1	1	-2.44409	-1.56823
52.94381	1.933296	2.525175	3.969231	0	0	1	1	-2.36718	-1.45611
45.57671	1.538581	2.219832	3.819397	0	0	1	1	-2.07553	-1.07747
54.33722	1.767444	2.331676	3.995209	0	0	1	1	-2.17967	-1.22883
71.54392	1.241338	2.240786	4.270311	0	0	1	1	-2.20102	-0.82068
74.0183	1.542505	2.476232	4.304312	0	0	1	1	-2.5187	-1.13949
74.7259	2.090296	2.46883	4.313827	0	0	1	1	-2.59467	-1.57071
78.75635	2.384019	2.545307	4.366359	0	0	1	1	-2.696	-1.82003
80.07683	2.596636	2.594635	4.382987	0	0	1	1	-2.62232	-1.90482
89.59436	2.881281	2.804169	4.495292	0	0	1	1	-3.05854	-2.40825
57.1807	-1.45379	1.58777	4.046216	0	0	1	1	-5.66801	2.714459
47.14333	-0.70951	2.13226	3.853193	0	0	1	1	-5.18678	1.256083
44.35332	0.988489	2.216145	3.792188	0	0	1	1	-4.80631	-1.62029
42.60667	1.316459	2.269939	3.752011	0	0	1	1	-4.62857	-2.08064
70.49286	0.883181	2.226909	4.255511	0	0	1	1	-5.63403	-1.50225
71.17284	1.000124	2.210523	4.265111	0	0	1	1	-5.09603	-1.53407
68.50861	1.476161	2.256585	4.226959	0	0	1	1	-5.10384	-2.27256
73.28826	2.006974	2.452431	4.2944	0	0	1	1	-5.29365	-3.09867
71.34986	2.346628	2.484021	4.267595	0	0	1	1	-4.88313	-3.32716

76.40972	2.477403	2.521708	4.33611	0	0	1	1	-4.95995	-3.56309
50.46449	0.552303	2.16204	3.92127	0	0	1	1	-6.48928	-1.20694
49.67931	1.110764	2.394251	3.905589	0	0	1	1	-5.9777	-2.20443
48.65643	0.921802	2.364089	3.884784	0	0	1	1	-6.08799	-1.87544
47.58789	0.917433	2.26502	3.862578	0	0	1	1	-6.12925	-1.90421
64.65158	0.765396	2.271015	4.169013	0	0	1	1	-7.5384	-1.79343
73.14997	-0.50445	2.356963	4.292512	0	0	1	1	-7.89393	1.193006
72.42637	1.51708	2.333817	4.28257	0	0	1	1	-7.21239	-3.24431
76.41247	1.988792	2.542796	4.336146	0	0	1	1	-6.81199	-3.90202
83.18446	2.192404	2.773956	4.421061	0	0	1	1	-7.00847	-4.28406
85.97892	2.478809	2.778311	4.454102	0	0	1	1	-6.15328	-4.22435
51.31102	1.418882	1.693917	3.937906	0	0	1	1	-3.70088	-1.74324
47.96172	1.00477	2.120533	3.870403	0	0	1	1	-3.34209	-1.12641
47.40851	1.614833	1.874515	3.858802	0	0	1	1	-3.24618	-1.75913
45.44392	1.688092	2.109569	3.816479	0	0	1	1	-3.03976	-1.72067
67.11293	0.943727	2.111712	4.206377	0	0	1	1	-3.06079	-0.88624
64.5988	1.40685	2.416416	4.168196	0	0	1	1	-3.80175	-1.62877
67.57214	1.879034	2.266147	4.213196	0	0	1	1	-4.13249	-2.33107
68.54218	2.278929	2.249568	4.227449	0	0	1	1	-4.42825	-2.98742
98.6228	2.847061	2.919801	4.591302	0	0	1	1	-4.92342	-3.68518
115.2034	2.74662	3.032097	4.746699	0	0	1	1	-5.10063	-3.61827
52.43571	1.592535	2.36011	3.959588	0	0	1	1	-2.70824	-1.38161
50.7908	1.683136	2.443988	3.927715	0	0	1	1	-2.69225	-1.45336
52.47932	1.65853	2.448137	3.960419	0	0	1	1	-2.92155	-1.54913
54.01165	1.403512	2.434878	3.9892	0	0	1	1	-3.10319	-1.3919
71.02515	1.565138	2.546186	4.263034	0	0	1	1	-3.75713	-1.74447
73.29227	1.749929	2.505314	4.294455	0	0	1	1	-3.81158	-1.95188
76.13297	1.872041	2.50603	4.332481	0	0	1	1	-4.17048	-2.25916
75.61248	2.177866	2.515592	4.325621	0	0	1	1	-4.54426	-2.8473
76.84019	2.49007	2.606291	4.341728	0	0	1	1	-0.78855	-0.55588
85.0541	2.685155	2.858708	4.443287	0	0	1	1	-4.33766	-3.23242

lnS_Gcre(	lnG_Gcre(	high	low	credithi{	creditlov	lnPopu	_cat	_est_m4	_est_m2
-1.97655	-3.17867	1	0	1	0	7.117206	1	1	1
-2.14313	-3.29851	1	0	1	0	7.143618	1	1	1
-2.11075	-3.20846	1	0	1	0	7.166266	1	1	1
-1.74031	-2.58459	1	0	1	0	7.187657	1	1	1
-0.9236	-1.42164	1	0	1	0	7.195187	1	1	1
-1.22896	-1.88853	1	0	1	0	7.198184	1	1	1
-1.0245	-1.56048	1	0	1	0	7.198184	1	1	1
-0.87565	-1.31481	1	0	1	0	7.197435	1	1	1
-0.65677	-0.98323	1	0	1	0	7.195937	1	1	1
-1.0742	-1.55179	1	0	1	0	7.195937	1	1	1
-2.11056	-3.66714	1	0	1	0	7.027315	1	1	1
-2.17337	-3.70939	1	0	1	0	7.05445	1	1	1
-1.9753	-3.21629	1	0	1	0	7.077498	1	1	1
-2.04676	-3.14171	1	0	1	0	7.09091	1	1	1
-1.97628	-3.18013	1	0	1	0	7.097549	1	1	1
-0.70072	-1.10585	1	0	1	0	7.100027	1	1	1
-1.04698	-1.64635	1	0	1	0	7.077498	1	1	1
-2.29782	-3.67421	1	0	1	0	7.057898	1	1	1
-1.55995	-2.42746	1	0	1	0	7.059618	1	1	1
-1.98918	-3.02995	1	0	1	0	7.060476	1	1	1
-1.68912	-3.24443	0	1	0	1	5.955837	1	1	0
-2.06704	-3.41291	0	1	0	1	5.961005	1	1	0
-2.07341	-3.41858	0	1	0	1	5.963579	1	1	0
-1.92254	-3.15792	0	1	0	1	5.968708	1	1	0
-1.90632	-3.31449	0	1	0	1	5.971262	1	1	0
-1.94474	-3.38965	0	1	0	1	5.976351	1	1	0
-1.94405	-3.31696	0	1	0	1	5.981414	1	1	0
-1.90985	-3.23913	0	1	0	1	5.983936	1	1	0
-2.02916	-3.34155	0	1	0	1	5.986452	1	1	0
-2.75347	-4.23347	0	1	0	1	5.988961	1	1	0
-1.69252	-3.00231	1	0	1	0	5.42495	1	1	1
-1.61627	-2.68114	1	0	1	0	5.420535	1	1	1
-1.35867	-2.21767	1	0	1	0	5.420535	1	1	1
-1.22888	-2.05874	1	0	1	0	5.416101	1	1	1
-1.15266	-2.06853	1	0	1	0	5.416101	1	1	1
-1.00678	-1.7957	1	0	1	0	5.411646	1	1	1
-1.00983	-1.82686	1	0	1	0	5.411646	1	1	1
-1.02335	-1.84105	1	0	1	0	5.407172	1	1	1
-1.1632	-2.07263	1	0	1	0	5.407172	1	1	1
-0.934	-1.61493	1	0	1	0	5.407172	1	1	1
-1.96981	-4.13094	0	1	0	1	3.044523	1	1	0
-2.16655	-3.87542	0	1	0	1	3.044523	1	1	0
-2.21665	-3.90849	0	1	0	1	3.044523	1	1	0
-2.64007	-4.4636	0	1	0	1	2.995732	1	1	0
-2.7943	-5.114	0	1	0	1	2.995732	1	1	0
-2.90686	-5.11323	0	1	0	1	2.995732	1	1	0
-2.85881	-4.99858	0	1	0	1	2.995732	1	1	0
-2.72	-4.86832	0	1	0	1	2.995732	1	1	0
-2.73631	-4.95719	0	1	0	1	2.995732	1	1	0
-2.94706	-5.1549	0	1	0	1	2.995732	1	1	0
-1.51998	-3.20438	0	1	0	1	5.69036	1	1	0
-1.70357	-3.21553	0	1	0	1	5.686975	1	1	0
-1.68385	-2.98607	0	1	0	1	5.686975	1	1	0

-1.82915	-3.11094	0	1	0	1	5.68358	1	1	0
-1.91942	-3.40876	0	1	0	1	5.680172	1	1	0
-1.90614	-3.35996	0	1	0	1	5.676754	1	1	0
-2.08176	-3.6887	0	1	0	1	5.673323	1	1	0
-2.21812	-3.91514	0	1	0	1	5.669881	1	1	0
-2.25267	-3.91165	0	1	0	1	5.666427	1	1	0
-2.38034	-3.87255	0	1	0	1	5.659482	1	1	0
-1.25606	-2.34826	0	1	1	0	4.962845	1	1	0
-1.18765	-2.1798	0	1	1	0	4.955827	1	1	0
-1.33164	-2.32312	0	1	1	0	4.941642	1	1	0
-1.56386	-2.61098	0	1	1	0	4.934474	1	1	0
-1.51644	-2.71044	0	1	1	0	4.919981	1	1	0
-1.46274	-2.65776	0	1	1	0	4.905275	1	1	0
-1.57549	-2.97856	0	1	1	0	4.890349	1	1	0
-1.79796	-3.38681	0	1	1	0	4.875197	1	1	0
-1.28276	-2.30263	0	1	1	0	4.859812	1	1	0
-1.75332	-2.70933	0	1	1	0	4.836282	1	1	0
-1.3805	-2.41091	0	1	1	0	4.454347	1	1	0
-1.48106	-2.45716	0	1	1	0	4.442651	1	1	0
-1.43644	-2.41346	0	1	1	0	4.41884	1	1	0
-1.34211	-2.29093	0	1	1	0	4.406719	1	1	0
-1.28794	-2.49006	0	1	1	0	4.382027	1	1	0
-1.22777	-2.56735	0	1	1	0	4.369448	1	1	0
-1.29872	-2.73942	0	1	1	0	4.343805	1	1	0
-1.39118	-2.84838	0	1	1	0	4.330733	1	1	0
-0.99375	-1.85791	0	1	1	0	4.304065	1	1	0
-1.12487	-2.16653	0	1	1	0	4.276666	1	1	0
-1.13242	-2.08574	1	0	1	0	8.220673	1	1	1
-1.10502	-1.79669	1	0	1	0	8.238801	1	1	1
-1.14806	-1.74432	1	0	1	0	8.258941	1	1	1
-0.95959	-1.41114	1	0	1	0	8.266679	1	1	1
-1.06743	-1.60848	1	0	1	0	8.263075	1	1	1
-1.16334	-1.79293	1	0	1	0	8.266679	1	1	1
-1.16322	-1.79079	1	0	1	0	8.266165	1	1	1
-1.16433	-1.77929	1	0	1	0	8.269757	1	1	1
-1.18748	-1.82187	1	0	1	0	8.272315	1	1	1
-1.2309	-1.79306	1	0	1	0	8.275122	1	1	1
-1.04229	-2.04553	1	0	1	0	6.664409	1	1	1
-1.21524	-2.00725	1	0	1	0	6.675823	1	1	1
-1.21144	-1.92312	1	0	1	0	6.684612	1	1	1
-1.18886	-1.84714	1	0	1	0	6.695799	1	1	1
-1.09795	-1.84818	1	0	1	0	6.699501	1	1	1
-1.04623	-1.87864	1	0	1	0	6.708084	1	1	1
-1.03348	-1.84411	1	0	1	0	6.712956	1	1	1
-1.03539	-1.79596	1	0	1	0	6.715384	1	1	1
-1.04003	-1.79644	1	0	1	0	6.717805	1	1	1
-1.16743	-1.83304	1	0	1	0	6.719013	1	1	1
-0.64566	-1.27444	0	1	1	0	6.287858	1	1	0
-0.74461	-1.24102	0	1	1	0	6.308098	1	1	0
-0.78423	-1.23728	0	1	1	0	6.326149	1	1	0
-0.85657	-1.31595	0	1	1	0	6.343881	1	1	0
-0.87914	-1.48711	0	1	1	0	6.359574	1	1	0
-0.84485	-1.53401	0	1	1	0	6.375025	1	1	0
-0.86259	-1.54747	0	1	1	0	6.390241	1	1	0

-0.89561	-1.54975	0	1	1	0	6.40688	1	1	0
-0.89102	-1.48687	0	1	1	0	6.423247	1	1	0
-1.00614	-1.59146	0	1	1	0	6.437752	1	1	0
-1.15837	-2.15351	1	0	1	0	6.059123	1	1	1
-1.16937	-1.923	1	0	1	0	6.059123	1	1	1
-1.19391	-1.96817	1	0	1	0	6.061457	1	1	1
-1.09071	-1.77336	1	0	1	0	6.063785	1	1	1
-1.0101	-1.7281	1	0	1	0	6.066108	1	1	1
-0.99185	-1.63951	1	0	1	0	6.068426	1	1	1
-0.99326	-1.7021	1	0	1	0	6.073044	1	1	1
-1.07101	-1.74598	1	0	1	0	6.075346	1	1	1
-1.39046	-2.19498	1	0	1	0	6.077642	1	1	1
-1.61469	-2.45084	1	0	1	0	6.079933	1	1	1
-1.48191	-2.2656	1	0	1	0	5.7301	1	1	1
-1.50939	-2.23821	1	0	1	0	5.746203	1	1	1
-1.63162	-2.31694	1	0	1	0	5.755742	1	1	1
-1.67292	-2.36097	1	0	1	0	5.771441	1	1	1
-1.76431	-2.64108	1	0	1	0	5.780744	1	1	1
-1.57516	-2.66383	1	0	1	0	5.78996	1	1	1
-1.51134	-2.55723	1	0	1	0	5.802118	1	1	1
-1.46229	-2.43861	1	0	1	0	5.811141	1	1	1
-1.64467	-2.62897	1	0	1	0	5.820083	1	1	1
-1.9283	-2.95927	1	0	1	0	5.826	1	1	1
-2.01926	-3.78598	1	0	0	1	5.590987	1	1	1
-2.30968	-3.8508	1	0	0	1	5.590987	1	1	1
-2.34896	-3.71593	1	0	0	1	5.590987	1	1	1
-2.27159	-3.64292	1	0	0	1	5.590987	1	1	1
-1.97029	-3.26394	1	0	0	1	5.590987	1	1	1
-1.74751	-3.05658	1	0	0	1	5.594711	1	1	1
-1.7592	-3.04184	1	0	0	1	5.598422	1	1	1
-1.7655	-2.96513	1	0	0	1	5.598422	1	1	1
-1.44901	-2.36057	1	0	0	1	5.598422	1	1	1
-1.63483	-2.51796	1	0	0	1	5.598422	1	1	1
-1.11722	-2.25249	1	0	1	0	6.415097	1	1	1
-1.23705	-2.0782	1	0	1	0	6.419995	1	1	1
-1.28576	-2.01604	1	0	1	0	6.423247	1	1	1
-1.2854	-2.02427	1	0	1	0	6.429719	1	1	1
-1.28795	-2.17415	1	0	1	0	6.436151	1	1	1
-1.15276	-2.04044	1	0	1	0	6.44572	1	1	1
-1.36853	-2.40594	1	0	1	0	6.452049	1	1	1
-1.65289	-2.8194	1	0	1	0	6.456769	1	1	1
-1.91012	-3.12629	1	0	1	0	6.459905	1	1	1
-2.39298	-3.62734	1	0	1	0	6.464588	1	1	1
-1.62005	-2.81337	1	0	1	0	6.340359	1	1	1
-1.9005	-2.86217	1	0	1	0	6.349139	1	1	1
-1.88526	-2.75815	1	0	1	0	6.352629	1	1	1
-1.82651	-2.66403	1	0	1	0	6.359574	1	1	1
-1.74343	-2.65285	1	0	1	0	6.36647	1	1	1
-1.51748	-2.50462	1	0	1	0	6.37332	1	1	1
-1.61718	-2.637	1	0	1	0	6.378426	1	1	1
-1.74109	-2.77251	1	0	1	0	6.381816	1	1	1
-1.86268	-2.93119	1	0	1	0	6.386879	1	1	1
-2.02958	-3.19145	1	0	1	0	6.390241	1	1	1
-1.95848	-3.73061	0	1	0	1	5.736572	1	1	0

-1.9164	-3.2586	0	1	0	1	5.739793	1	1	0
-1.67497	-2.84509	0	1	0	1	5.743003	1	1	0
-1.72318	-2.82133	0	1	0	1	5.746203	1	1	0
-1.67139	-2.9519	0	1	0	1	5.752573	1	1	0
-1.55578	-2.69789	0	1	0	1	5.758902	1	1	0
-1.46415	-2.55981	0	1	0	1	5.762052	1	1	0
-1.47808	-2.43788	0	1	0	1	5.765191	1	1	0
-1.30677	-2.14846	0	1	0	1	5.765191	1	1	0
-1.52261	-2.39417	0	1	0	1	5.733341	1	1	0
-1.8843	-3.42828	0	1	1	0	5.739793	1	1	0
-1.73826	-2.8931	0	1	1	0	5.739793	1	1	0
-1.86723	-2.98648	0	1	1	0	5.743003	1	1	0
-1.67768	-2.59091	0	1	1	0	5.743003	1	1	0
-1.55082	-2.61273	0	1	1	0	5.743003	1	1	0
-1.46853	-2.55818	0	1	1	0	5.746203	1	1	0
-1.34759	-2.41609	0	1	1	0	5.746203	1	1	0
-1.29444	-2.24257	0	1	1	0	5.746203	1	1	0
-1.39431	-2.3804	0	1	1	0	5.746203	1	1	0
-1.63594	-2.64616	0	1	1	0	5.749393	1	1	0
-1.41904	-2.41403	1	0	1	0	6.424869	1	1	1
-1.49982	-2.23046	1	0	1	0	6.452049	1	1	1
-1.45495	-2.14458	1	0	1	0	6.472346	1	1	1
-1.4227	-2.06602	1	0	1	0	6.490724	1	1	1
-1.32766	-2.06523	1	0	1	0	6.507277	1	1	1
-1.21393	-1.93167	1	0	1	0	6.526495	1	1	1
-1.12498	-1.79169	1	0	1	0	6.546785	1	1	1
-1.07259	-1.66832	1	0	1	0	6.563856	1	1	1
-1.12274	-1.72828	1	0	1	0	6.575076	1	1	1
-1.22304	-1.78543	1	0	1	0	6.584791	1	1	1
-1.98995	-3.83203	1	0	0	1	5.278115	1	1	1
-2.06142	-3.53835	1	0	0	1	5.288267	1	1	1
-2.14006	-3.3944	1	0	0	1	5.293305	1	1	1
-2.09715	-3.25462	1	0	0	1	5.303305	1	1	1
-2.17544	-3.38233	1	0	0	1	5.313206	1	1	1
-2.02523	-3.35147	1	0	0	1	5.32301	1	1	1
-2.07903	-3.50805	1	0	0	1	5.332719	1	1	1
-2.16308	-3.54475	1	0	0	1	5.337538	1	1	1
-2.31338	-3.78549	1	0	0	1	5.347107	1	1	1
-2.72775	-4.29597	1	0	0	1	5.351858	1	1	1
-1.07108	-2.32902	0	1	0	1	5.659482	1	1	0
-1.45781	-2.64116	0	1	0	1	5.68358	1	1	0
-1.34647	-2.5392	0	1	0	1	5.693732	1	1	0
-0.93713	-1.54806	0	1	0	1	5.710427	1	1	0
-2.05238	-3.11468	0	1	0	1	5.720312	1	1	0
-2.45492	-3.96311	0	1	0	1	5.733341	1	1	0
-2.42921	-3.96877	0	1	0	1	5.749393	1	1	0
-2.26841	-3.91071	0	1	0	1	5.758902	1	1	0
-2.17148	-3.87981	0	1	0	1	5.771441	1	1	0
-2.4368	-4.18604	0	1	0	1	5.78996	1	1	0
-1.21684	-2.58517	0	1	1	0	5.877736	1	1	0
-1.63498	-2.90554	0	1	1	0	5.888878	1	1	0
-1.44626	-2.60475	0	1	1	0	5.902633	1	1	0
-1.71975	-2.71443	0	1	1	0	5.910797	1	1	0
-1.4764	-2.73099	0	1	1	0	5.921578	1	1	0

-1.39649	-2.51904	0	1	1	0	5.934894	1	1	0
-1.37935	-2.44359	0	1	1	0	5.945421	1	1	0
-1.4372	-2.33399	0	1	1	0	5.950643	1	1	0
-1.55646	-2.41952	0	1	1	0	5.958425	1	1	0
-1.47222	-2.32918	0	1	1	0	5.966147	1	1	0
-1.68866	-3.01057	0	1	0	1	5.099866	1	1	0
-1.709	-2.71448	0	1	0	1	5.099866	1	1	0
-1.7933	-2.84049	0	1	0	1	5.105946	1	1	0
-2.30212	-3.5155	0	1	0	1	5.111988	1	1	0
-1.93366	-3.34547	0	1	0	1	5.117994	1	1	0
-1.98346	-3.42019	0	1	0	1	5.123964	1	1	0
-1.9938	-3.39679	0	1	0	1	5.129899	1	1	0
-2.02528	-3.30094	0	1	0	1	5.129899	1	1	0
-2.11383	-3.32888	0	1	0	1	5.135798	1	1	0
-2.26097	-3.38821	0	1	0	1	5.135798	1	1	0
-2.97728	-5.63893	0	1	0	1	5.298317	0	1	0
-3.19606	-5.17004	0	1	0	1	5.31812	0	1	0
-2.95487	-4.83665	0	1	0	1	5.327876	0	1	0
-2.55681	-4.31943	0	1	0	1	5.342334	1	1	0
-2.7701	-5.10189	0	1	0	1	5.347107	0	1	0
-2.63927	-4.85321	0	1	0	1	5.361292	1	1	0
-2.80877	-5.07267	0	1	0	1	5.375278	1	1	0
-2.91588	-5.22948	0	1	0	1	5.379897	1	1	0
-3.00759	-5.29739	0	1	0	1	5.384495	0	1	0
-3.4148	-5.5548	0	1	0	1	5.389072	0	1	0
-3.20369	-5.18732	1	0	0	1	4.762174	0	1	1
-3.59924	-5.79141	1	0	0	1	4.770685	0	1	1
-3.97451	-6.09123	1	0	0	1	4.770685	0	1	1
-4.04864	-6.09031	1	0	0	1	4.770685	0	1	1
-4.57192	-7.30083	1	0	0	1	4.770685	0	1	1
-4.4672	-7.14368	1	0	0	1	4.779123	0	1	1
-4.32176	-6.90969	1	0	0	1	4.779123	0	1	1
-4.08934	-6.63416	1	0	0	1	4.779123	0	1	1
-4.25715	-6.86762	1	0	0	1	4.787492	0	1	1
-4.56329	-6.97698	1	0	0	1	4.787492	0	1	1
-1.87899	-3.09247	1	0	0	1	5.209486	1	1	1
-1.9019	-2.98952	1	0	0	1	5.214936	1	1	1
-1.55454	-2.67472	1	0	0	1	5.220356	1	1	1
-1.62112	-2.7777	1	0	0	1	5.225747	1	1	1
-1.48145	-2.82322	1	0	0	1	5.231109	1	1	1
-1.82925	-3.1797	1	0	0	1	5.236442	1	1	1
-1.85515	-3.24154	1	0	0	1	5.247024	1	1	1
-1.94316	-3.3334	1	0	0	1	5.252274	1	1	1
-1.90335	-3.21524	1	0	0	1	5.257495	1	1	1
-2.3438	-3.75728	1	0	0	1	5.257495	1	1	1
-2.96462	-7.55493	0	1	0	1	4.025352	0	1	0
-3.77483	-6.82146	0	1	0	1	4.025352	0	1	0
-3.6326	-6.21598	0	1	0	1	4.025352	0	1	0
-3.5876	-5.92999	0	1	0	1	4.025352	0	1	0
-3.78788	-7.23844	0	1	0	1	4.007333	0	1	0
-3.39068	-6.54217	0	1	0	1	4.007333	0	1	0
-3.47402	-6.50742	0	1	0	1	4.007333	0	1	0
-3.78643	-6.63034	0	1	0	1	4.007333	0	1	0
-3.52197	-6.0508	0	1	0	1	4.007333	0	1	0

-3.62681	-6.23636	0	1	0	1	4.007333	0	1	0
-4.72466	-8.56907	0	1	0	1	2.079442	0	1	0
-4.75165	-7.75106	0	1	0	1	2.079442	0	1	0
-4.80984	-7.90375	0	1	0	1	2.079442	0	1	0
-4.70123	-8.01709	0	1	0	1	2.079442	0	1	0
-5.3213	-9.76856	0	1	0	1	2.079442	0	1	0
-5.57411	-10.1516	0	1	0	1	2.079442	0	1	0
-4.99092	-9.15837	0	1	0	1	2.079442	0	1	0
-4.98898	-8.50754	0	1	0	1	2.079442	0	1	0
-5.42044	-8.63896	0	1	0	1	2.079442	0	1	0
-4.73476	-7.59062	0	1	0	1	2.197225	0	1	0
-2.08115	-4.83811	0	1	0	1	4.584968	1	1	0
-2.37725	-4.33897	0	1	0	1	4.59512	1	1	0
-2.04202	-4.20362	0	1	0	1	4.60517	1	1	0
-2.15029	-3.89014	0	1	0	1	4.624973	1	1	0
-1.98307	-3.95013	0	1	0	1	4.634729	1	1	0
-2.79759	-4.8257	0	1	0	1	4.65396	1	1	0
-2.81131	-5.22675	0	1	0	1	4.663439	0	1	0
-2.94894	-5.54172	0	1	0	1	4.672829	0	1	0
-3.77933	-5.94289	0	1	0	1	4.682131	0	1	0
-3.99435	-6.25309	0	1	0	1	4.691348	0	1	0
-2.04753	-3.43516	1	0	0	1	2.639057	1	1	1
-2.11035	-3.39152	1	0	0	1	2.639057	1	1	1
-2.28665	-3.69918	1	0	0	1	2.639057	1	1	1
-2.41473	-3.95619	1	0	0	1	2.639057	1	1	1
-2.83792	-4.75148	1	0	0	1	2.70805	1	1	1
-2.79445	-4.79007	1	0	0	1	2.70805	1	1	1
-3.02425	-5.22839	1	0	0	1	2.70805	1	1	1
-3.28884	-5.65524	1	0	0	1	2.70805	0	1	1
-0.58183	-0.96924	1	0	0	1	2.772589	1	1	1
-3.44135	-5.34888	1	0	0	1	2.772589	1	1	1

_est_m3	_est_firs	dyear1	dyear2	dyear3	dyear4	dyear5	dyear6	dyear7	dyear8
0	1	1	0	0	0	0	0	0	0
0	1	0	1	0	0	0	0	0	0
0	1	0	0	1	0	0	0	0	0
0	1	0	0	0	1	0	0	0	0
0	1	0	0	0	0	1	0	0	0
0	1	0	0	0	0	0	1	0	0
0	1	0	0	0	0	0	0	1	0
0	1	0	0	0	0	0	0	0	1
0	1	0	0	0	0	0	0	0	0
0	1	0	0	0	0	0	0	0	0
0	1	1	0	0	0	0	0	0	0
0	1	0	1	0	0	0	0	0	0
0	1	0	0	1	0	0	0	0	0
0	1	0	0	0	1	0	0	0	0
0	1	0	0	0	0	1	0	0	0
0	1	0	0	0	0	0	1	0	0
0	1	0	0	0	0	0	0	1	0
0	1	0	0	0	0	0	0	0	1
0	1	0	0	0	0	0	0	0	0
0	1	0	0	0	0	0	0	0	0
1	1	1	0	0	0	0	0	0	0
1	1	0	1	0	0	0	0	0	0
1	1	0	0	1	0	0	0	0	0
1	1	0	0	0	1	0	0	0	0
1	1	0	0	0	0	1	0	0	0
1	1	0	0	0	0	0	1	0	0
1	1	0	0	0	0	0	0	1	0
1	1	0	0	0	0	0	0	0	1
1	1	0	0	0	0	0	0	0	0
1	1	0	0	0	0	0	0	0	0
0	1	1	0	0	0	0	0	0	0
0	1	0	1	0	0	0	0	0	0
0	1	0	0	1	0	0	0	0	0
0	1	0	0	0	1	0	0	0	0
0	1	0	0	0	0	1	0	0	0
0	1	0	0	0	0	0	1	0	0
0	1	0	0	0	0	0	0	1	0
0	1	0	0	0	0	0	0	0	1
0	1	0	0	0	0	0	0	0	0
0	1	0	0	0	0	0	0	0	0
1	1	1	0	0	0	0	0	0	0
1	1	0	1	0	0	0	0	0	0
1	1	0	0	1	0	0	0	0	0
1	1	0	0	0	1	0	0	0	0
1	1	0	0	0	0	1	0	0	0
1	1	0	0	0	0	0	1	0	0
1	1	0	0	0	0	0	0	1	0
1	1	0	0	0	0	0	0	0	1
1	1	0	0	0	0	0	0	0	0
1	1	0	0	0	0	0	0	0	0
1	1	1	0	0	0	0	0	0	0
1	1	0	1	0	0	0	0	0	0
1	1	0	0	1	0	0	0	0	0

1	1	0	0	0	1	0	0	0	0
1	1	0	0	0	0	1	0	0	0
1	1	0	0	0	0	0	1	0	0
1	1	0	0	0	0	0	0	1	0
1	1	0	0	0	0	0	0	0	1
1	1	0	0	0	0	0	0	0	0
1	1	0	0	0	0	0	0	0	0
1	1	1	0	0	0	0	0	0	0
1	1	0	1	0	0	0	0	0	0
1	1	0	0	1	0	0	0	0	0
1	1	0	0	0	1	0	0	0	0
1	1	0	0	0	0	1	0	0	0
1	1	0	0	0	0	0	1	0	0
1	1	0	0	0	0	0	0	1	0
1	1	0	0	0	0	0	0	0	1
1	1	0	0	0	0	0	0	0	0
1	1	0	0	0	0	0	0	0	0
1	1	1	0	0	0	0	0	0	0
1	1	0	1	0	0	0	0	0	0
1	1	0	0	1	0	0	0	0	0
1	1	0	0	0	1	0	0	0	0
1	1	0	0	0	0	1	0	0	0
1	1	0	0	0	0	0	1	0	0
1	1	0	0	0	0	0	0	1	0
1	1	0	0	0	0	0	0	0	1
1	1	0	0	0	0	0	0	0	0
1	1	0	0	0	0	0	0	0	0
0	1	1	0	0	0	0	0	0	0
0	1	0	1	0	0	0	0	0	0
0	1	0	0	1	0	0	0	0	0
0	1	0	0	0	1	0	0	0	0
0	1	0	0	0	0	1	0	0	0
0	1	0	0	0	0	0	1	0	0
0	1	0	0	0	0	0	0	1	0
0	1	0	0	0	0	0	0	0	1
0	1	0	0	0	0	0	0	0	0
0	1	0	0	0	0	0	0	0	0
0	1	1	0	0	0	0	0	0	0
0	1	0	1	0	0	0	0	0	0
0	1	0	0	1	0	0	0	0	0
0	1	0	0	0	1	0	0	0	0
0	1	0	0	0	0	1	0	0	0
0	1	0	0	0	0	0	1	0	0
0	1	0	0	0	0	0	0	1	0
0	1	0	0	0	0	0	0	0	1
0	1	0	0	0	0	0	0	0	0
0	1	0	0	0	0	0	0	0	0
1	1	1	0	0	0	0	0	0	0
1	1	0	1	0	0	0	0	0	0
1	1	0	0	1	0	0	0	0	0
1	1	0	0	0	1	0	0	0	0
1	1	0	0	0	0	1	0	0	0
1	1	0	0	0	0	0	1	0	0
1	1	0	0	0	0	0	0	1	0

1	1	0	0	0	0	0	0	0	1
1	1	0	0	0	0	0	0	0	0
1	1	0	0	0	0	0	0	0	0
0	1	1	0	0	0	0	0	0	0
0	1	0	1	0	0	0	0	0	0
0	1	0	0	1	0	0	0	0	0
0	1	0	0	0	1	0	0	0	0
0	1	0	0	0	0	1	0	0	0
0	1	0	0	0	0	0	1	0	0
0	1	0	0	0	0	0	0	1	0
0	1	0	0	0	0	0	0	0	1
0	1	0	0	0	0	0	0	0	0
0	1	0	0	0	0	0	0	0	0
0	1	1	0	0	0	0	0	0	0
0	1	0	1	0	0	0	0	0	0
0	1	0	0	1	0	0	0	0	0
0	1	0	0	0	1	0	0	0	0
0	1	0	0	0	0	1	0	0	0
0	1	0	0	0	0	0	1	0	0
0	1	0	0	0	0	0	0	1	0
0	1	0	0	0	0	0	0	0	1
0	1	0	0	0	0	0	0	0	0
0	1	0	0	0	0	0	0	0	0
0	1	1	0	0	0	0	0	0	0
0	1	0	1	0	0	0	0	0	0
0	1	0	0	1	0	0	0	0	0
0	1	0	0	0	1	0	0	0	0
0	1	0	0	0	0	1	0	0	0
0	1	0	0	0	0	0	1	0	0
0	1	0	0	0	0	0	0	1	0
0	1	0	0	0	0	0	0	0	1
0	1	0	0	0	0	0	0	0	0
0	1	0	0	0	0	0	0	0	0
0	1	1	0	0	0	0	0	0	0
0	1	0	1	0	0	0	0	0	0
0	1	0	0	1	0	0	0	0	0
0	1	0	0	0	1	0	0	0	0
0	1	0	0	0	0	1	0	0	0
0	1	0	0	0	0	0	1	0	0
0	1	0	0	0	0	0	0	1	0
0	1	0	0	0	0	0	0	0	1
0	1	0	0	0	0	0	0	0	0
0	1	0	0	0	0	0	0	0	0
0	1	1	0	0	0	0	0	0	0
0	1	0	1	0	0	0	0	0	0
0	1	0	0	1	0	0	0	0	0
0	1	0	0	0	1	0	0	0	0
0	1	0	0	0	0	1	0	0	0
0	1	0	0	0	0	0	1	0	0
0	1	0	0	0	0	0	0	1	0
0	1	0	0	0	0	0	0	0	1
0	1	0	0	0	0	0	0	0	0
0	1	0	0	0	0	0	0	0	0
1	1	1	0	0	0	0	0	0	0

1	1	0	1	0	0	0	0	0	0
1	1	0	0	1	0	0	0	0	0
1	1	0	0	0	1	0	0	0	0
1	1	0	0	0	0	1	0	0	0
1	1	0	0	0	0	0	1	0	0
1	1	0	0	0	0	0	0	1	0
1	1	0	0	0	0	0	0	0	1
1	1	0	0	0	0	0	0	0	0
1	1	0	0	0	0	0	0	0	0
1	1	1	0	0	0	0	0	0	0
1	1	0	1	0	0	0	0	0	0
1	1	0	0	1	0	0	0	0	0
1	1	0	0	0	1	0	0	0	0
1	1	0	0	0	0	1	0	0	0
1	1	0	0	0	0	0	1	0	0
1	1	0	0	0	0	0	0	1	0
1	1	0	0	0	0	0	0	0	1
1	1	0	0	0	0	0	0	0	0
1	1	0	0	0	0	0	0	0	0
0	1	1	0	0	0	0	0	0	0
0	1	0	1	0	0	0	0	0	0
0	1	0	0	1	0	0	0	0	0
0	1	0	0	0	1	0	0	0	0
0	1	0	0	0	0	1	0	0	0
0	1	0	0	0	0	0	1	0	0
0	1	0	0	0	0	0	0	1	0
0	1	0	0	0	0	0	0	0	1
0	1	0	0	0	0	0	0	0	0
0	1	0	0	0	0	0	0	0	0
0	1	1	0	0	0	0	0	0	0
0	1	0	1	0	0	0	0	0	0
0	1	0	0	1	0	0	0	0	0
0	1	0	0	0	1	0	0	0	0
0	1	0	0	0	0	1	0	0	0
0	1	0	0	0	0	0	1	0	0
0	1	0	0	0	0	0	0	1	0
0	1	0	0	0	0	0	0	0	1
0	1	0	0	0	0	0	0	0	0
0	1	0	0	0	0	0	0	0	0
1	1	1	0	0	0	0	0	0	0
1	1	0	1	0	0	0	0	0	0
1	1	0	0	1	0	0	0	0	0
1	1	0	0	0	1	0	0	0	0
1	1	0	0	0	0	1	0	0	0
1	1	0	0	0	0	0	1	0	0
1	1	0	0	0	0	0	0	1	0
1	1	0	0	0	0	0	0	0	1
1	1	0	0	0	0	0	0	0	0
1	1	0	0	0	0	0	0	0	0
1	1	1	0	0	0	0	0	0	0
1	1	0	1	0	0	0	0	0	0
1	1	0	0	1	0	0	0	0	0
1	1	0	0	0	1	0	0	0	0
1	1	0	0	0	0	1	0	0	0

1	1	0	0	0	0	0	1	0	0
1	1	0	0	0	0	0	0	1	0
1	1	0	0	0	0	0	0	0	1
1	1	0	0	0	0	0	0	0	0
1	1	0	0	0	0	0	0	0	0
1	1	1	0	0	0	0	0	0	0
1	1	0	1	0	0	0	0	0	0
1	1	0	0	1	0	0	0	0	0
1	1	0	0	0	1	0	0	0	0
1	1	0	0	0	0	1	0	0	0
1	1	0	0	0	0	0	1	0	0
1	1	0	0	0	0	0	0	1	0
1	1	0	0	0	0	0	0	0	1
1	1	0	0	0	0	0	0	0	0
1	1	0	0	0	0	0	0	0	0
1	1	1	0	0	0	0	0	0	0
1	1	0	1	0	0	0	0	0	0
1	1	0	0	1	0	0	0	0	0
1	1	0	0	0	1	0	0	0	0
1	1	0	0	0	0	1	0	0	0
1	1	0	0	0	0	0	1	0	0
1	1	0	0	0	0	0	0	1	0
1	1	0	0	0	0	0	0	0	1
1	1	0	0	0	0	0	0	0	0
1	1	0	0	0	0	0	0	0	0
0	1	1	0	0	0	0	0	0	0
0	1	0	1	0	0	0	0	0	0
0	1	0	0	1	0	0	0	0	0
0	1	0	0	0	1	0	0	0	0
0	1	0	0	0	0	1	0	0	0
0	1	0	0	0	0	0	1	0	0
0	1	0	0	0	0	0	0	1	0
0	1	0	0	0	0	0	0	0	1
0	1	0	0	0	0	0	0	0	0
0	1	0	0	0	0	0	0	0	0
0	1	1	0	0	0	0	0	0	0
0	1	0	1	0	0	0	0	0	0
0	1	0	0	1	0	0	0	0	0
0	1	0	0	0	1	0	0	0	0
0	1	0	0	0	0	1	0	0	0
0	1	0	0	0	0	0	1	0	0
0	1	0	0	0	0	0	0	1	0
0	1	0	0	0	0	0	0	0	1
0	1	0	0	0	0	0	0	0	0
0	1	0	0	0	0	0	0	0	0
1	1	1	0	0	0	0	0	0	0
1	1	0	1	0	0	0	0	0	0
1	1	0	0	1	0	0	0	0	0
1	1	0	0	0	1	0	0	0	0
1	1	0	0	0	0	1	0	0	0
1	1	0	0	0	0	0	1	0	0
1	1	0	0	0	0	0	0	1	0
1	1	0	0	0	0	0	0	0	1
1	1	0	0	0	0	0	0	0	0

1	1	0	0	0	0	0	0	0	0
1	1	1	0	0	0	0	0	0	0
1	1	0	1	0	0	0	0	0	0
1	1	0	0	1	0	0	0	0	0
1	1	0	0	0	1	0	0	0	0
1	1	0	0	0	0	1	0	0	0
1	1	0	0	0	0	0	1	0	0
1	1	0	0	0	0	0	0	1	0
1	1	0	0	0	0	0	0	0	1
1	1	0	0	0	0	0	0	0	0
1	1	0	0	0	0	0	0	0	0
1	1	1	0	0	0	0	0	0	0
1	1	0	1	0	0	0	0	0	0
1	1	0	0	1	0	0	0	0	0
1	1	0	0	0	1	0	0	0	0
1	1	0	0	0	0	1	0	0	0
1	1	0	0	0	0	0	1	0	0
1	1	0	0	0	0	0	0	1	0
1	1	0	0	0	0	0	0	0	1
1	1	0	0	0	0	0	0	0	0
1	1	0	0	0	0	0	0	0	0
0	1	1	0	0	0	0	0	0	0
0	1	0	1	0	0	0	0	0	0
0	1	0	0	1	0	0	0	0	0
0	1	0	0	0	1	0	0	0	0
0	1	0	0	0	0	1	0	0	0
0	1	0	0	0	0	0	1	0	0
0	1	0	0	0	0	0	0	1	0
0	1	0	0	0	0	0	0	0	1
0	1	0	0	0	0	0	0	0	0
0	1	0	0	0	0	0	0	0	0

dyear9	dyear10	f_eff	_est_a	_est_b	_est_m1
0	0	0.229178	1	0	1
0	0	0.22425	1	0	1
0	0	0.230575	1	0	1
0	0	0.229041	1	0	1
0	0	0.192724	1	0	1
0	0	0.195372	1	0	1
0	0	0.207397	1	0	1
0	0	0.210742	1	0	1
1	0	0.207206	1	0	1
0	1	0.191105	1	0	1
0	0	0.461281	1	0	1
0	0	0.445604	1	0	1
0	0	0.426538	1	0	1
0	0	0.429442	1	0	1
0	0	0.386492	1	0	1
0	0	0.38172	1	0	1
0	0	0.402401	1	0	1
0	0	0.431296	1	0	1
1	0	0.462586	1	0	1
0	1	0.41025	1	0	1
0	0	0.723342	0	1	1
0	0	0.673655	0	1	1
0	0	0.615032	0	1	1
0	0	0.576019	0	1	1
0	0	0.53954	0	1	1
0	0	0.509113	0	1	1
0	0	0.506868	0	1	1
0	0	0.493013	0	1	1
1	0	0.479918	0	1	1
0	1	0.445191	0	1	1
0	0	0.520755	1	0	1
0	0	0.485773	1	0	1
0	0	0.459187	1	0	1
0	0	0.451641	1	0	1
0	0	0.417567	1	0	1
0	0	0.393344	1	0	1
0	0	0.445937	1	0	1
0	0	0.456109	1	0	1
1	0	0.441922	1	0	1
0	1	0.41969	1	0	1
0	0	0.784014	0	1	1
0	0	0.769144	0	1	1
0	0	0.749221	0	1	1
0	0	0.749694	0	1	1
0	0	0.7163	0	1	1
0	0	0.651493	0	1	1
0	0	0.649073	0	1	1
0	0	0.693888	0	1	1
1	0	0.727953	0	1	1
0	1	0.691149	0	1	1
0	0	0.530446	0	1	1
0	0	0.505576	0	1	1
0	0	0.48731	0	1	1

0	0	0.476199	0	1	1
0	0	0.423179	0	1	1
0	0	0.394496	0	1	1
0	0	0.399878	0	1	1
0	0	0.398376	0	1	1
1	0	0.396433	0	1	1
0	1	0.367879	0	1	1
0	0	0.711284	1	0	1
0	0	0.682979	1	0	1
0	0	0.63782	1	0	1
0	0	0.607712	1	0	1
0	0	0.541526	1	0	1
0	0	0.496432	1	0	1
0	0	0.506524	1	0	1
0	0	0.51324	1	0	1
1	0	0.485507	1	0	1
0	1	0.451921	1	0	1
0	0	0.693378	1	0	1
0	0	0.674715	1	0	1
0	0	0.653498	1	0	1
0	0	0.632092	1	0	1
0	0	0.550924	1	0	1
0	0	0.536318	1	0	1
0	0	0.521404	1	0	1
0	0	0.507327	1	0	1
1	0	0.488673	1	0	1
0	1	0.431292	1	0	1
0	0	0.35613	1	0	1
0	0	0.335229	1	0	1
0	0	0.335047	1	0	1
0	0	0.342026	1	0	1
0	0	0.259126	1	0	1
0	0	0.270444	1	0	1
0	0	0.292766	1	0	1
0	0	0.297342	1	0	1
1	0	0.286008	1	0	1
0	1	0.249981	1	0	1
0	0	0.743101	1	0	1
0	0	0.711458	1	0	1
0	0	0.693301	1	0	1
0	0	0.691632	1	0	1
0	0	0.660554	1	0	1
0	0	0.638701	1	0	1
0	0	0.660827	1	0	1
0	0	0.667112	1	0	1
1	0	0.645502	1	0	1
0	1	0.595709	1	0	1
0	0	0.523126	1	0	1
0	0	0.51564	1	0	1
0	0	0.506354	1	0	1
0	0	0.50508	1	0	1
0	0	0.481805	1	0	1
0	0	0.474771	1	0	1
0	0	0.488286	1	0	1

0	0	0.497824	1	0	1
1	0	0.475723	1	0	1
0	1	0.424932	1	0	1
0	0	0.833102	1	0	1
0	0	0.790199	1	0	1
0	0	0.76412	1	0	1
0	0	0.74844	1	0	1
0	0	0.684288	1	0	1
0	0	0.636615	1	0	1
0	0	0.643082	1	0	1
0	0	0.664287	1	0	1
1	0	0.677582	1	0	1
0	1	0.635441	1	0	1
0	0	0.850975	1	0	1
0	0	0.831453	1	0	1
0	0	0.802453	1	0	1
0	0	0.811187	1	0	1
0	0	0.753863	1	0	1
0	0	0.753884	1	0	1
0	0	0.790808	1	0	1
0	0	0.86593	1	0	1
1	0	0.849311	1	0	1
0	1	0.773382	1	0	1
0	0	0.808859	0	1	1
0	0	0.760596	0	1	1
0	0	0.730245	0	1	1
0	0	0.720195	0	1	1
0	0	0.670084	0	1	1
0	0	0.631797	0	1	1
0	0	0.621188	0	1	1
0	0	0.643697	0	1	1
1	0	0.633266	0	1	1
0	1	0.591219	0	1	1
0	0	0.842908	1	0	1
0	0	0.791089	1	0	1
0	0	0.76266	1	0	1
0	0	0.752239	1	0	1
0	0	0.74189	1	0	1
0	0	0.70446	1	0	1
0	0	0.711746	1	0	1
0	0	0.706788	1	0	1
1	0	0.673489	1	0	1
0	1	0.615113	1	0	1
0	0	0.98771	1	0	1
0	0	0.915112	1	0	1
0	0	0.841476	1	0	1
0	0	0.835646	1	0	1
0	0	0.778589	1	0	1
0	0	0.745667	1	0	1
0	0	0.758861	1	0	1
0	0	0.781866	1	0	1
1	0	0.772821	1	0	1
0	1	0.709772	1	0	1
0	0	0.8327	0	1	1

0	0	0.806634	0	1	1
0	0	0.777604	0	1	1
0	0	0.78117	0	1	1
0	0	0.74197	0	1	1
0	0	0.705362	0	1	1
0	0	0.720097	0	1	1
0	0	0.758914	0	1	1
1	0	0.760347	0	1	1
0	1	0.640336	0	1	1
0	0	0.972787	1	0	1
0	0	0.916192	1	0	1
0	0	0.876069	1	0	1
0	0	0.855422	1	0	1
0	0	0.78791	1	0	1
0	0	0.734663	1	0	1
0	0	0.723917	1	0	1
0	0	0.741504	1	0	1
1	0	0.757573	1	0	1
0	1	0.71734	1	0	1
0	0	0.57946	1	0	1
0	0	0.542417	1	0	1
0	0	0.522232	1	0	1
0	0	0.533095	1	0	1
0	0	0.465947	1	0	1
0	0	0.456896	1	0	1
0	0	0.471115	1	0	1
0	0	0.480388	1	0	1
1	0	0.464542	1	0	1
0	1	0.415305	1	0	1
0	0	0.761381	0	1	1
0	0	0.707948	0	1	1
0	0	0.676524	0	1	1
0	0	0.669399	0	1	1
0	0	0.64921	0	1	1
0	0	0.632572	0	1	1
0	0	0.637667	0	1	1
0	0	0.658877	0	1	1
1	0	0.671084	0	1	1
0	1	0.638124	0	1	1
0	0	0.546302	0	1	1
0	0	0.545899	0	1	1
0	0	0.523452	0	1	1
0	0	0.53657	0	1	1
0	0	0.489661	0	1	1
0	0	0.450456	0	1	1
0	0	0.445461	0	1	1
0	0	0.510973	0	1	1
1	0	0.547435	0	1	1
0	1	0.539759	0	1	1
0	0	0.629999	1	0	1
0	0	0.596964	1	0	1
0	0	0.571658	1	0	1
0	0	0.581229	1	0	1
0	0	0.557374	1	0	1

0	0	0.560416	1	0	1
0	0	0.575732	1	0	1
0	0	0.585263	1	0	1
1	0	0.597869	1	0	1
0	1	0.584338	1	0	1
0	0	0.601949	0	1	1
0	0	0.575379	0	1	1
0	0	0.551058	0	1	1
0	0	0.552523	0	1	1
0	0	0.51017	0	1	1
0	0	0.4954	0	1	1
0	0	0.518684	0	1	1
0	0	0.554355	0	1	1
1	0	0.566907	0	1	1
0	1	0.536816	0	1	1
0	0	0.57638	0	1	1
0	0	0.639678	0	1	1
0	0	0.60106	0	1	1
0	0	0.600994	0	1	1
0	0	0.54227	0	1	1
0	0	0.496083	0	1	1
0	0	0.521502	0	1	1
0	0	0.57995	0	1	1
1	0	0.617187	0	1	1
0	1	0.631638	0	1	1
0	0	0.617715	0	1	1
0	0	0.616246	0	1	1
0	0	0.617566	0	1	1
0	0	0.625289	0	1	1
0	0	0.597561	0	1	1
0	0	0.590382	0	1	1
0	0	0.616943	0	1	1
0	0	0.684188	0	1	1
1	0	0.707582	0	1	1
0	1	0.691627	0	1	1
0	0	0.633224	0	1	1
0	0	0.624176	0	1	1
0	0	0.621857	0	1	1
0	0	0.619056	0	1	1
0	0	0.552172	0	1	1
0	0	0.540221	0	1	1
0	0	0.568321	0	1	1
0	0	0.590175	0	1	1
1	0	0.579145	0	1	1
0	1	0.526083	0	1	1
0	0	0.569315	0	1	1
0	0	0.532407	0	1	1
0	0	0.498278	0	1	1
0	0	0.467001	0	1	1
0	0	0.402255	0	1	1
0	0	0.394385	0	1	1
0	0	0.412705	0	1	1
0	0	0.433873	0	1	1
1	0	0.44102	0	1	1

0	1	0.427753	0	1	1
0	0	0.484955	0	1	1
0	0	0.433192	0	1	1
0	0	0.417622	0	1	1
0	0	0.407897	0	1	1
0	0	0.385785	0	1	1
0	0	0.405408	0	1	1
0	0	0.423077	0	1	1
0	0	0.477526	0	1	1
1	0	0.503043	0	1	1
0	1	0.477535	0	1	1
0	0	0.651126	0	1	1
0	0	0.609657	0	1	1
0	0	0.601711	0	1	1
0	0	0.587756	0	1	1
0	0	0.536799	0	1	1
0	0	0.511142	0	1	1
0	0	0.547205	0	1	1
0	0	0.582279	0	1	1
1	0	0.580222	0	1	1
0	1	0.554396	0	1	1
0	0	0.628863	0	1	1
0	0	0.601076	0	1	1
0	0	0.595692	0	1	1
0	0	0.615361	0	1	1
0	0	0.543502	0	1	1
0	0	0.513707	0	1	1
0	0	0.52499	0	1	1
0	0	0.581941	0	1	1
1	0	0.583742	0	1	1
0	1	0.555926	0	1	1
