# Supplementary material for: Research on the impact of ESG performance on carbon emissions from the perspective of green credit
Source: Sci Rep. 2024 May 7;14:10478. doi: 10.1038/s41598-024-61353-3 (PMC11636868; doi:10.1038/s41598-024-61353-3)
Supplement: Supplementary file 2 — Supplementary Information 2. [file 41598_2024_61353_MOESM2_ESM.pdf]

```
use "..\20240426.dta"

***Table 1. Descriptive statistics of the variables****
logout,save(mytable) word replace:tabstat Ce ESG Gcredit Gov Regu Pgdp Popu Ele, stat(N mean sd min max) format(%10.4f)    column(stat)

***Table 2 and Table 3 ****
xthreg lnCe lnRegu lnGov lnPgdp lnPopu lnEle, rx(lnESG) qx(lnGcredit) thnum(3) grid(350) trim(0.13 0.13 0.13) bs(350 350 350)
est store m1
esttab m1 using    "table2.rtf" ,nogap replace scalar (`ss' F) b(%6.4f) se(%6.4f) star(* 0.1    ** 0.05 *** 0.01 )    r2 pr2

***Table 4 The threshold regression results ****
xthreg lnCe lnRegu lnGov lnPgdp lnPopu lnEle, rx(lnESG) qx(lnGcredit) thnum(1) grid(350) trim(0.13) bs(350)
_matplot  e(LR),yline(7.35,  lpattern(dash))  connect(direct)  msize(small)  mlabp(0)  mlabs(zero) ytitle("LR Statistics") xtitle("First Threshold") recast(line) name(R)
xthreg lnCe lnRegu lnGov lnPgdp lnPopu lnEle, rx(lnESG) qx(lnGcredit) thnum(1) grid(350) trim(0.13) bs(350)
  est store m1
xthreg lnCe lnRegu lnGov lnPgdp lnPopu lnEle, rx(lnE) qx(lnGcredit) thnum(1) grid(350) trim(0.13) bs(350)
  est store m2
xthreg lnCe lnRegu lnGov lnPgdp lnPopu lnEle, rx(lnS) qx(lnGcredit) thnum(1) grid(350) trim(0.13) bs(350)
  est store m3
xthreg lnCe lnRegu lnGov lnPgdp lnPopu lnEle, rx(lnG) qx(lnGcredit) thnum(1) grid(350) trim(0.13) bs(350)
  est store m4
esttab m1 m2 m3 m4    using    "table4.rtf" ,nogap replace scalar (`ss' F) b(%6.4f) se(%6.4f) star(* 0.1    ** 0.05    *** 0.01 )    r2 pr2

***Table 5    The fixed effects regression results ****
xtreg lnCe lnESG lnRegu lnGov lnPgdp lnPopu lnEle, fe
est store m1
xtreg lnCe lnRegu lnGov lnPgdp lnPopu lnEle lnGcredit, fe
est store m2
xtreg lnCe lnESG lnRegu lnGov lnPgdp lnPopu lnEle lnGcredit, fe
est store m3
xtreg lnCe lnESG lnRegu lnGov lnPgdp lnPopu lnEle lnGcredit lnESG_Gcredit , fe
est store m4
esttab m1 m2 m3 m4 using    "table5.rtf" ,nogap replace scalar (`ss' F) b(%6.4f) se(%6.4f) star(* 0.1
```

```
** 0.05    *** 0.01 )    r2 pr2

***Table 6    The threshold regression results of the moderating effect ****
xthreg lnCe lnRegu lnGov lnPgdp lnPopu lnEle lnGcredit, rx(lnESG_Gcredit) qx(lnGcredit) thnum(1) grid(350) trim(0.13) bs(350)
est store m1
xthreg lnCe lnRegu lnGov lnPgdp lnPopu lnEle lnGcredit, rx(lnE_Gcredit) qx(lnGcredit) thnum(1) grid(350) trim(0.13) bs(350)
est store m2
xthreg lnCe lnRegu lnGov lnPgdp lnPopu lnEle lnGcredit, rx(lnS_Gcredit) qx(lnGcredit) thnum(1) grid(350) trim(0.13) bs(350)
est store m3
xthreg lnCe lnRegu lnGov lnPgdp lnPopu lnEle lnGcredit, rx(lnG_Gcredit) qx(lnGcredit) thnum(1) grid(350) trim(0.13) bs(350)
est store m4
esttab m1 m2 m3 m4 using    "table6.rtf" ,nogap replace scalar (`ss' F) b(%6.4f) se(%6.4f) star(* 0.1    ** 0.05    *** 0.01 )    r2 pr2


***Table 7 endogeneity test ****
xtivreg2 lnCe lnESG lnESG_Gcredit lnRegu lnPgdp lnEle (lnGcredit = f_eff) dyear*, fe    first
est store m1
esttab m1 using    "table7.rtf" ,nogap replace scalar (`ss' F) b(%6.4f) se(%6.4f) star(* 0.1    ** 0.05    *** 0.01 )    r2 pr2

***Table 8 heterogeneity test ****
***Suest grouped by ESG***
reg lnCe lnESG lnRegu lnGov lnPgdp lnPopu lnEle lnGcredit lnESG_Gcredit    if high == 1
est store a
reg lnCe lnESG lnRegu lnGov lnPgdp lnPopu lnEle lnGcredit lnESG_Gcredit    if high == 0
est store b
suest a b
test [a_mean]lnGcredit =    [b_mean]lnGcredit

reg lnCe lnESG lnRegu lnGov lnPgdp lnPopu lnEle lnGcredit lnESG_Gcredit    if high == 1
est store a
reg lnCe lnESG lnRegu lnGov lnPgdp lnPopu lnEle lnGcredit lnESG_Gcredit    if high == 0
est store b
suest a b
test [a_mean]lnESG_Gcredit    =    [b_mean]lnESG_Gcredit

***Suest grouped by Gcredit ***
reg lnCe lnESG lnRegu lnGov lnPgdp lnPopu lnEle lnGcredit lnESG_Gcredit    if credithigh == 1
est store a
```

```
reg lnCe lnESG lnRegu lnGov lnPgdp lnPopu lnEle lnGcredit lnESG_Gcredit    if credithigh == 0
est store b
suest a b
test [a_mean]lnGcredit    =    [b_mean]lnGcredit

reg lnCe lnESG lnRegu lnGov lnPgdp lnPopu lnEle lnGcredit lnESG_Gcredit    if credithigh == 1
est store a
reg lnCe lnESG lnRegu lnGov lnPgdp lnPopu lnEle lnGcredit lnESG_Gcredit    if credithigh == 0
est store b
suest a b
test [a_mean]lnESG_Gcredit    =    [b_mean]lnESG_Gcredit

***Fisher's Combination grouped by ESG***
bdiff, group(high) model(xtreg lnCe lnESG lnRegu lnGov lnPgdp lnPopu lnEle lnGcredit lnESG_Gcredit, fe ) bs reps(1000)

***Fisher's Combination grouped by Gcredit***
bdiff, group(credithigh) model(xtreg lnCe lnESG lnRegu lnGov lnPgdp lnPopu lnEle lnGcredit lnESG_Gcredit, fe ) bs reps(1000)


***Table 9    The moderating effect in provinces with different ESG levels    ****
xtreg lnCe lnESG lnRegu lnGov lnPgdp lnPopu lnEle lnGcredit lnESG_Gcredit , fe
est store m1
xtreg lnCe lnESG lnRegu lnGov lnPgdp lnPopu lnEle lnGcredit lnESG_Gcredit , fe, if high == 1
est store m2
xtreg lnCe lnESG lnRegu lnGov lnPgdp lnPopu lnEle lnGcredit lnESG_Gcredit , fe, if low == 1
est store m3
esttab m1 m2 m3    using    "table9.rtf" ,nogap replace scalar (`ss' F) b(%6.4f) se(%6.4f) star(* 0.1 ** 0.05    *** 0.01 )    r2 pr2

***Table 10 The moderating effect in provinces with different green credit levels    ****
xtreg lnCe lnESG lnRegu lnGov lnPgdp lnPopu lnEle lnGcredit lnESG_Gcredit , fe
est store m1
xtreg lnCe lnESG lnRegu lnGov lnPgdp lnPopu lnEle lnGcredit lnESG_Gcredit , fe, if credithigh == 1
est store m2
xtreg lnCe lnESG lnRegu lnGov lnPgdp lnPopu lnEle lnGcredit lnESG_Gcredit , fe, if creditlow == 1
est store m3
esttab m1 m2 m3    using    "table10.rtf" ,nogap replace scalar (`ss' F) b(%6.4f) se(%6.4f) star(* 0.1 ** 0.05    *** 0.01 )    r2 pr2
```
